# Xylanins A–P, sixteen new guaiane-type dimers from the branches and leaves of *Xylopia vielana* with anti-proliferative activity against PANC-1 cell line

**DOI:** 10.1007/s13659-025-00574-z

**Published:** 2026-01-11

**Authors:** Xianglian Jiang, Ting Zhang, Fancheng Meng, Min Chen, Guowei Wang

**Affiliations:** College of Pharmaceutical Sciences, Southwest University, Chongqing, 400715 People’s Republic of China

**Keywords:** Structural identification, Sesquiterpenes, *Xylopia vielana* branches and leaves, Anti-proliferative activity

## Abstract

Sixteen previously undiscovered guaiane-type sesquiterpene dimers, xylanins A–P (**1–16**), along with six known analogues (**17–22**), were isolated from the branches and leaves of *Xylopia vielana* with chromatographic techniques. Their structures including absolute configurations were determined by high-resolution electrospray ionization mass spectrometry (HR-ESI–MS), nuclear magnetic resonance (NMR) data, electron circular dichroism (ECD) spectra, as well as X-ray crystallographic analysis. In cytotoxicity test, we found that five compounds (**6**, **7**, **8**, **9** and **12**) had cytotoxic activities in vitro against the human pancreatic cancer (PANC-1) and human prostate cancer (PC-3) cell lines. Of these compounds, compound **8** showed a relatively greater cytotoxic effect against PANC-1 cell lines with half maximal inhibitory concentration (IC_50_) value of 1.06 μM. Flow cytometry analysis showed that the apoptosis rate of PANC-1 cells increased with increasing concentrations of compound **8**, and also demonstrated that the cell cycle of PANC-1 cells was arrested at S phase by the treatment of compound **8**. By the invasion test, compound **8** was found to restrain the invasion of PANC-1 cells. In autophagy assay, we observed increased microtubule-associated protein 1 light chain 3 **(**LC3) by immunofluorescence in the compound **8**-treated group.

**Graphical Abstract:**

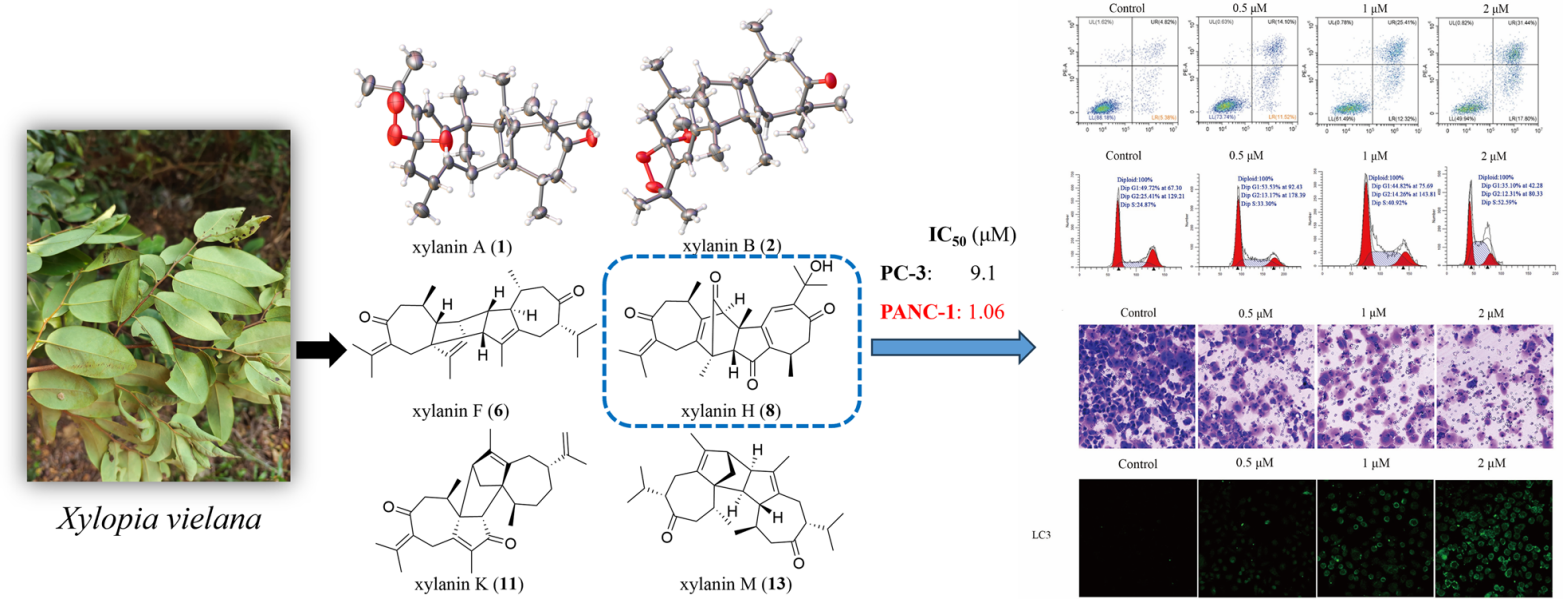

**Supplementary Information:**

The online version contains supplementary material available at 10.1007/s13659-025-00574-z.

## Introduction

**Fig. 1:**
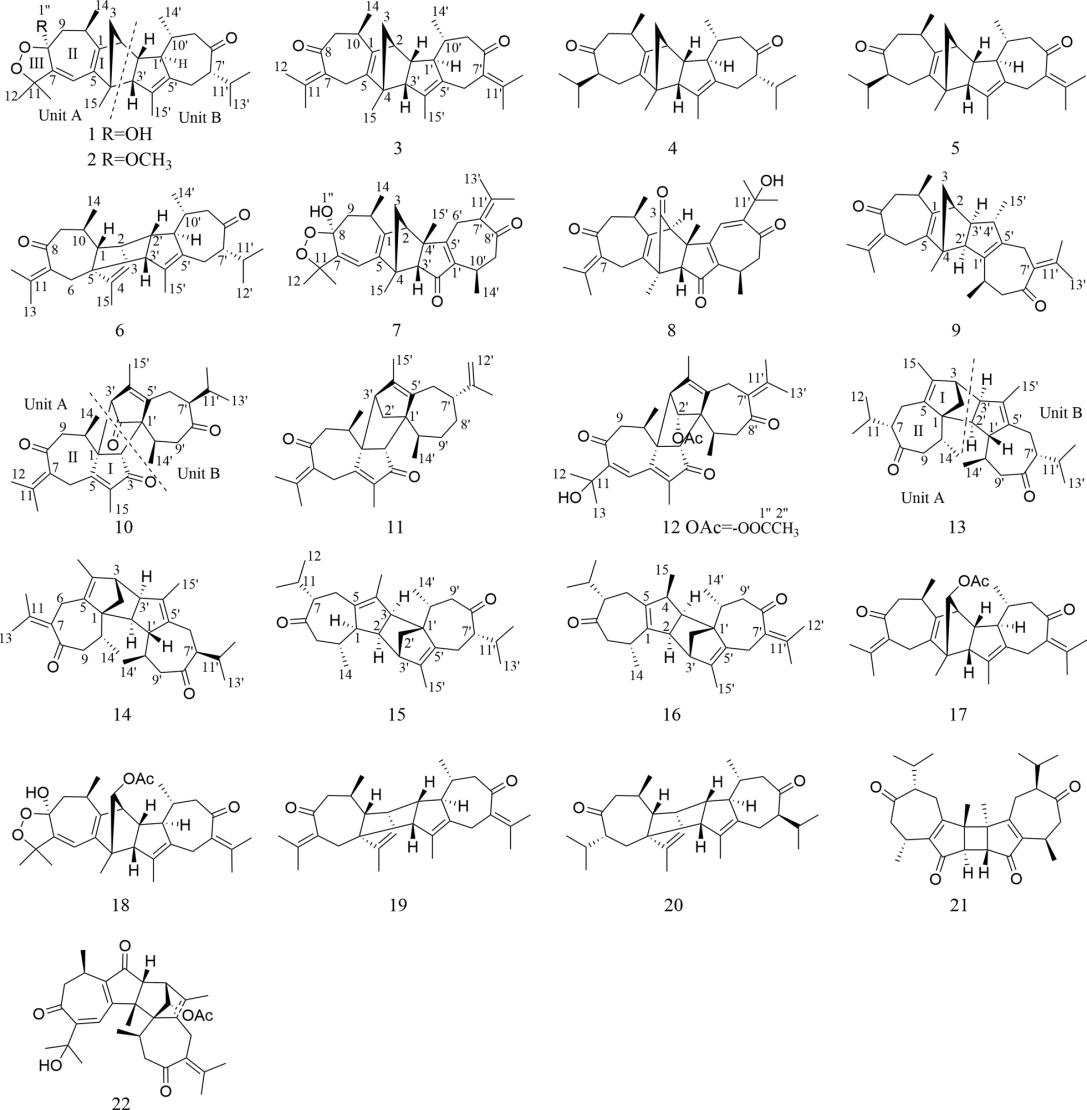
Structures of compounds **1–22**

*Xylopia vielana*, belonging to the genus *Xylopia* (Annonaceae), produced in southern Guangxi, Vietnam and Cambodia. So far, there are about 73 compounds, including sesquiterpene dimers and alkaloids with anti-inflammatory, antitumor and multidrug resistance reversal activities, found in *X. vielana* [1–13]. In order to understand the chemical substances of *X. vielana* and enrich the compound library of this genus, the chemical constituents of the branches and leaves of *X. vielana* were isolated and identified in this study. We isolated 16 new guaiane-type dimers (xylanins A–P, **1–16**, Fig. 1) and 6 known compounds (**17–22**) from *X. vielana* extracts. To find the potentially active compounds, all the new sesquiterpene dimers were tested for cytotoxic activities and five of them (**6**, **7**, **8**, **9** and **12**) showed cytotoxic activities in vitro against the PANC-1 and PC-3 cell lines. Within these five compounds, compound **8** had a relatively stronger cytotoxic effect against PANC-1 and PC-3 cell lines with IC_50_ values of 1.06 and 9.1 μM, respectively. Further flow cytometry analysis, transwell assay and immunofluorescence technique revealed that compound **8** had an inhibitory effect in PANC-1 cells. The details of isolation, structure identification and biological evaluation of these sesquiterpene dimers are reported here.

## Results and discussion

### Structure elucidation

**Fig. 2:**
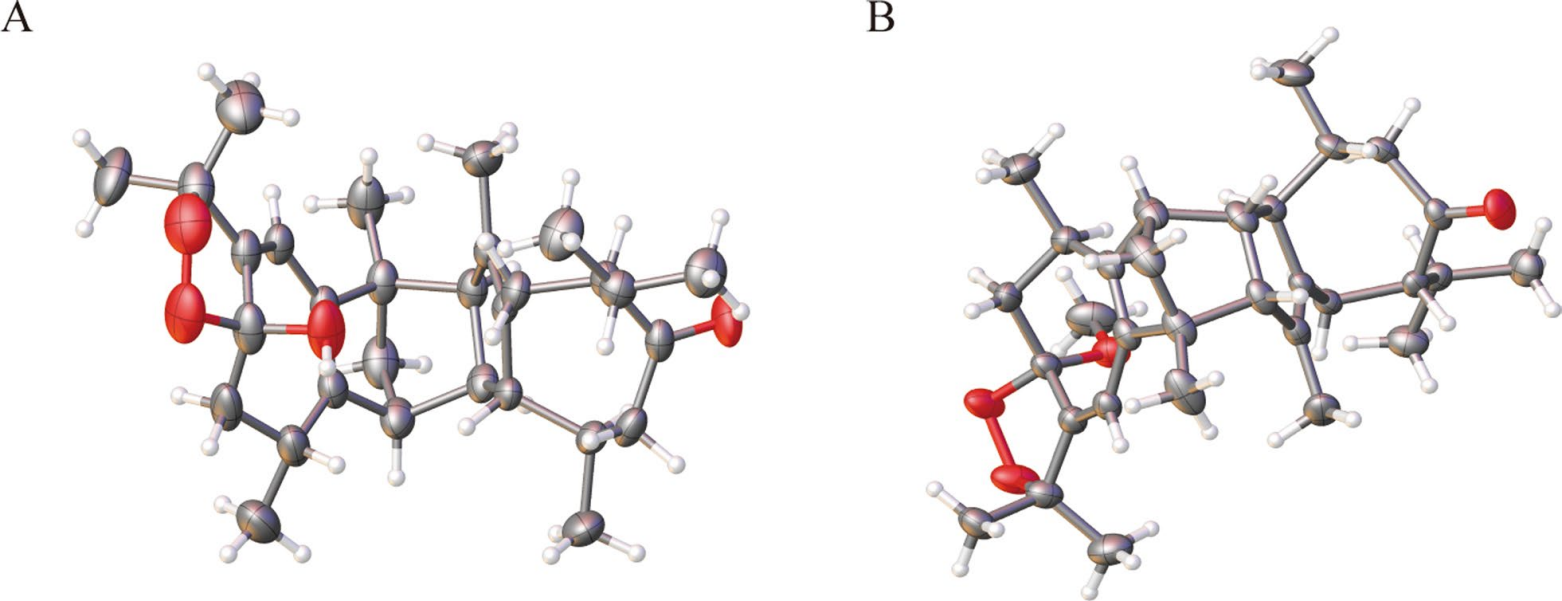
X-ray structures of **1** (**A**) and **2** (**B**)

**Fig. 3:**
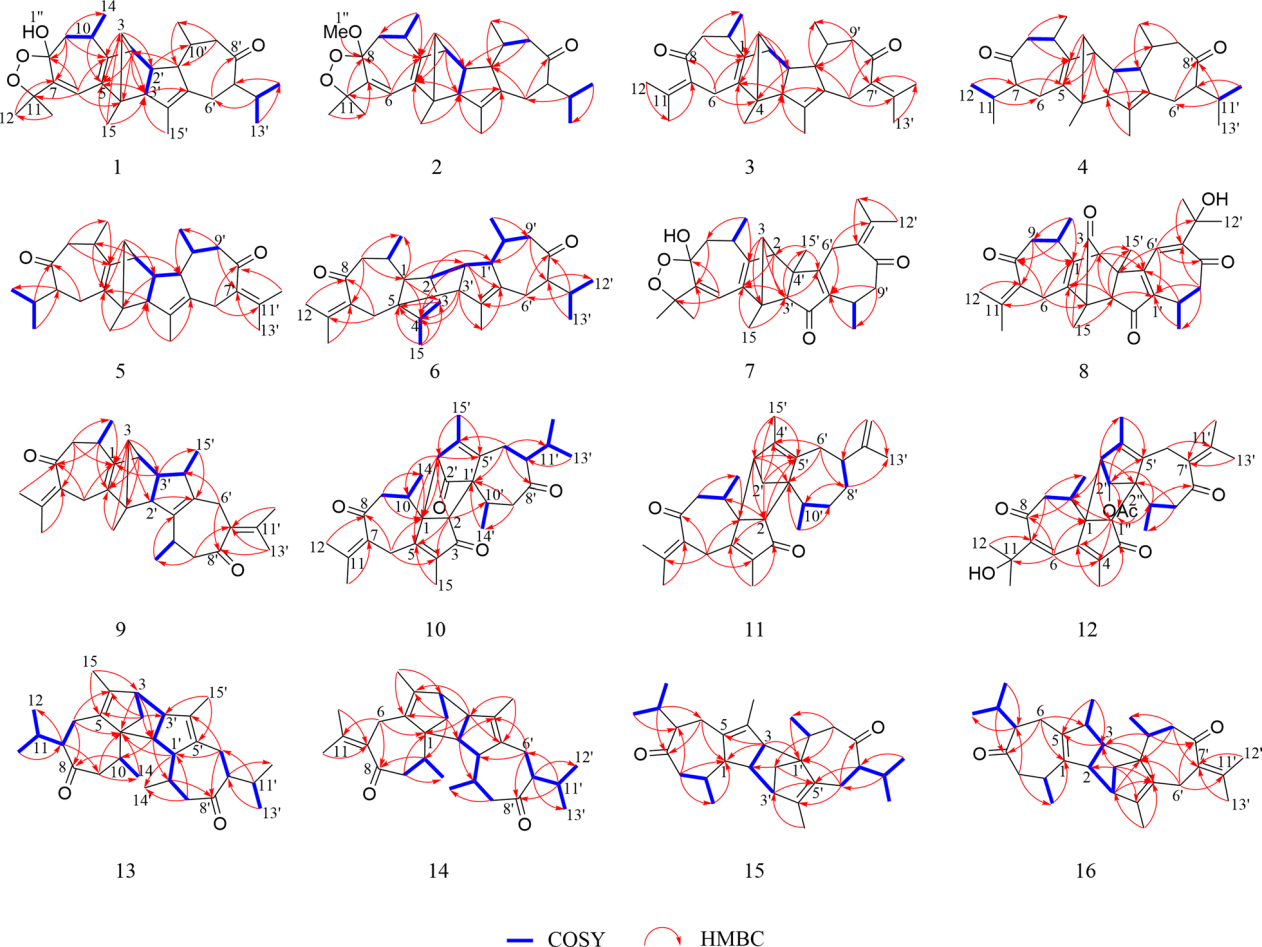
Key HMBC and ^1^H-^1^H COSY correlations of **1–16**

**Fig. 4:**
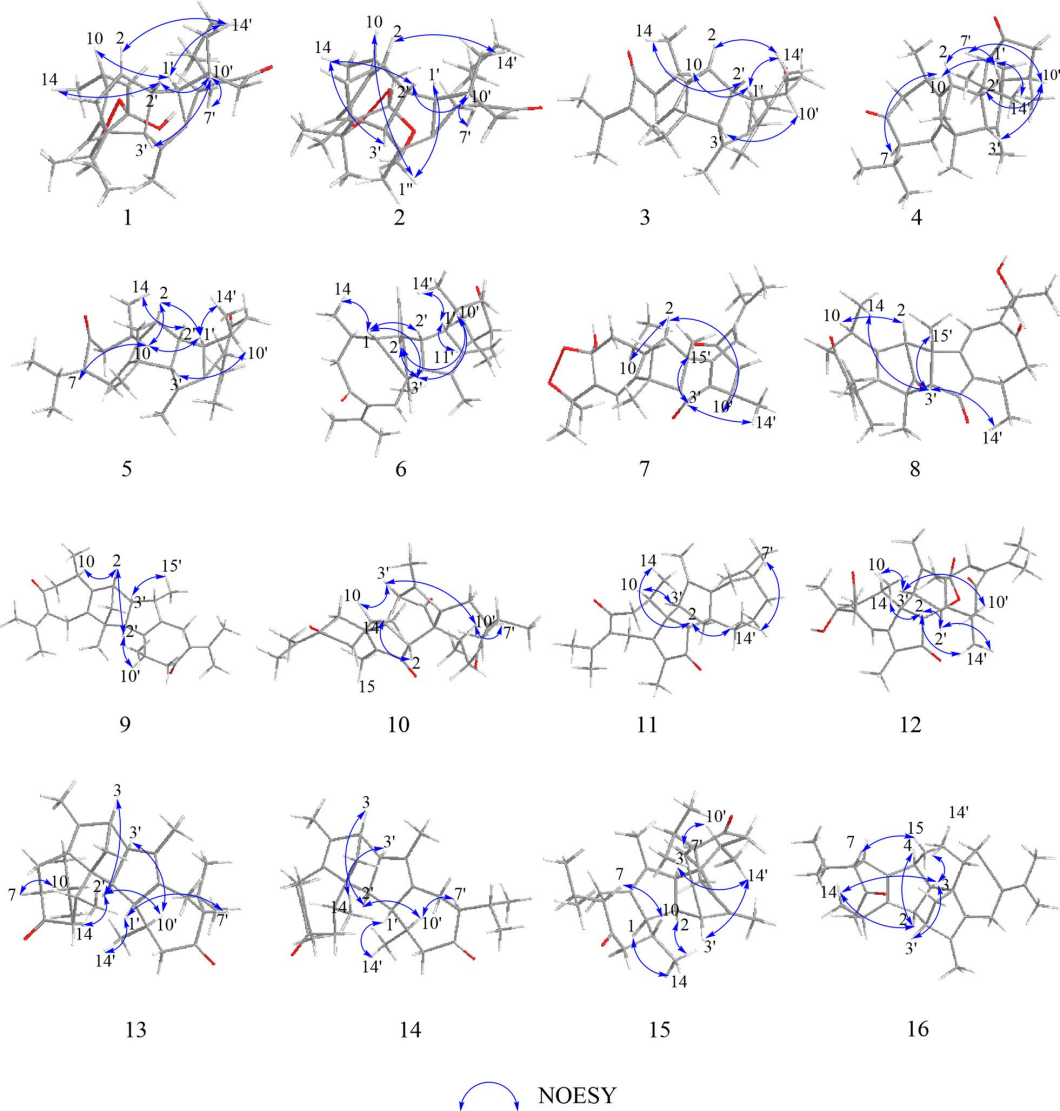
Key NOESY correlations of **1–16**

Xylanin A (**1**) was obtained as colorless needle crystals. The molecular formula of **1** was found to be C_30_H_42_O_4_ (HR-ESI–MS [M + Na] ^+^ at *m/z* 489.2977; calcd for 489.2975), indicating 10 degrees of unsaturation. The ^13^C NMR and DEPT data (Table 1) revealed the presence of 30 carbons, comprising eight methyls, four methylenes, eight methines and ten quaternary carbons. The 1D NMR data (Table 1) revealed the presence of two guaiane units (A and B), which was further confirmed by 2D NMR experiments (Fig. 3). In the HMBC spectrum, the five-membered ring (I) was deduced by the cross-peaks from H_2_-3 to C-1/C-5, from H_3_-15 to C-3/C-4/C-5, and from H-2 to C-5 in unit A. Moreover, the HMBC cross-peaks from H-6 to C-1/C-4/C-7/C-8, from H-10 to C-1/C-5/C-9/C-14, and from H_2_-9 to C-1/C-7/C-8/C-10/C-14 indicated that the seven-membered ring (II) was fused with the five-membered ring (I) via the double bond (C-1/C-5). Furthermore, another five-membered ring (III) was connected to the seven-membered ring (II) through a C-7/C-8 single bond. This was based on the key HMBC correlations from H-6 to C-7/C-8/C-11, from H_2_-9 to C-7/C-8, from H_3_-12 to C-7, and from H_3_-13 to C-11. The presence of a dioxygen bridge in the five-membered ring (III) was determined on the basis of the chemical shifts of C-8 (*δ*c 103.3) and C-11 (*δ*c 85.3). The above results were used to elucidate the structure of unit A, which was shown to be a guaiane-type sesquiterpene. Similarly, unit B was assigned as a guaiane-type based on the key HMBC cross-peaks. The linkage of unit A and unit B via two direct C–C bonds (C-2 to C-2′ and C-4 to C-3′) was deduced from the key HMBC cross-peaks from H_3_-15 to C-3′, from H-1′ to C-2, and from H-2 to C-2′/C-3′. Consequently, the planar structure of **1** was confirmed (Fig. 3). The relative configuration of **1** was identified by the key NOESY correlations (Fig. 4). The key NOESY correlations of H-1′/H_3_-14′, H-2/H_3_-14′ and H-10/H-1′ suggested that these protons shared the same spatial orientation and were assigned as *α*-oriented. The correlations of H-2′/H-10′, H_3_-14/H-2′, H-3′/H-10′ and H-10′/H-7′ indicated that H-2′, H-3′, H-10′ and H-7′ were *β*-oriented. Finally, the absolute configuration of **1** (Fig. 2) was determined by single-crystal X-ray diffraction analysis as 2*S*, 4*R*, 8*S*, 10*R*, 1′*R*, 2′*R*, 3′*R*, 7′*S*, 10′*R*.

**Table 1:** ^1^H (400 MHz) and ^13^C (100 MHz) NMR data for compounds **1–4** in CDCl_3_

Position	**1**		**2**		**3**		**4**	
	*δ*_H_ (*J* in Hz)	*δ*_C_	*δ*_H_ (*J* in Hz)	*δ*_C_	*δ*_H_ (*J* in Hz)	*δ*_C_	*δ*_H_ (*J* in Hz)	*δ*_C_
1		150.9		149.6		143.8		143.4
2	2.83 d (4.2)	48.4	2.81 d (2.9)	48.4	2.25–2.29 m	51.4	2.56 d (4.2)	47.1
3a	1.32 dd (1.6, 6.4)	56.1	1.28–1.30 m^a^	56.3	1.23–1.26 m	56.7	1.17–1.22 m	57.0
3b	1.24–1.26 m^a^		1.25–1.27 m		1.20 dd (0.9, 6.7)		1.17–1.22 m	
4		55.3		55.3		57.2		57.5
5		132.6		133.1		137.8		138.8
6a	5.56 s	112.8	5.53 s	113.2	3.15 d (17.1)	25.9	2.30–2.37 m	23.5
6b					2.78 d (17.1)		1.96–1.99 m	
7		153.6		151.4		135.4	1.90–1.96 m^a^	58.4
8		103.3		106.1		204.7		214.8
9a	2.08 dd (5.8, 7.7)	39.0	2.16 dd (4.6, 9.0)	36.4	2.63–2.68 m^a^	48.5	2.78 t (11.2)	48.5
9b	1.65–1.69 m		1.64–1.65 m		2.56 d (4.4)		2.25 dd (4.8, 6.4)	
10	2.74–2.76 m	33.6	2.51–2.57 m	33.0	2.45–2.49 m	35.5	2.45–2.50 m	35.6
11		85.3		85.4		139.7	1.87–1.90 m	30.6
12	1.42 s	24.9	1.37–1.40 m^a^	24.0	1.84 d (0.7)	22.5	0.85–0.89 m^a^	19.5
13	1.39–1.40 m^a^	27.7	1.37–1.40 m^a^	28.7	1.99 s	22.8	1.12 d (6.8)	20.8
14	1.24–1.26 m^a^	19.2	1.22 d (7.1)	19.5	1.10 d (6.8)	20.3	0.85–0.89 m^a^	21.2
15	1.39–1.40 m^a^	18.5	1.37–1.40 m^a^	18.6	1.34–1.36 m^a^	18.5	1.29 s	18.4
1′	1.73–1.77 m	57.8		58.5	1.58–1.61 m	58.1	1.42–1.46 m	58.2
2′	2.30 q (4.5)	52.1	2.27–2.31 m	51.8	2.51–2.53 m	47.2	2.18–2.22 m	51.7
3′	2.76–2.78 m	61.5	2.73 d (8.2)	61.4	2.63–2.68 m^a^	61.9	2.66–2.70 m	62.2
4′		136.5		135.4		136.3		136.2
5′		137.9		138.0		137.9		138.4
6a′	2.27 d (5.2)	26.5	2.43 dd (4.9, 8.8)	26.9	3.23 d (15.7)	28.4	2.42 dd (4.2, 8.8)	26.4
6b′	1.81–1.86 m		1.77–1.80 m		2.63–2.68 m^a^		1.84–1.87 m	
7′	1.90–1.97 m	58.9	1.91–1.95 m	58.9		133.8	1.90–1.96 m^a^	58.9
8′		216.1		216.5		206.4		216.5
9a′	2.67–2.70 m	48.3	2.65 t (11.3)	48.3	2.68–2.71 m	51.0	2.62–2.65 m	48.2
9b′	2.04 dd (1.8, 8.9)		2.05 d (10.5)		2.17 dd (1.9, 9.4)		1.99–2.02 m	
10′	1.24–1.26 m^a^	41.8	1.28–1.30 m^a^	41.7	1.34–1.36 m^a^	41.6	1.23–1.25 m	41.8
11′	1.77–1.80 m	31.8	1.80–1.83 m	31.9		139.2	1.77–1.83 m	31.8
12′	0.90 d (6.7)	19.7	0.91 d (6.7)	19.7	1.81 s	22.6	0.94 d (6.6)	19.7
13′	0.86 d (6.8)	20.6	0.88 d (6.8)	20.9	1.95 d (1.9)	23.0	0.85–0.89 m^a^	21.0
14′	1.05 d (6.5)	22.1	1.05 d (6.5)	22.1	1.01 d (6.5)	21.9	0.99 d (6.5)	22.0
15′	1.44 s	13.5	1.42 s	13.5	1.42 s	14.1	1.50 s	13.8
-OH								
-OCH_3_			3.36 s	49.3				

^a^Overlapped signals

**Fig. 5:**
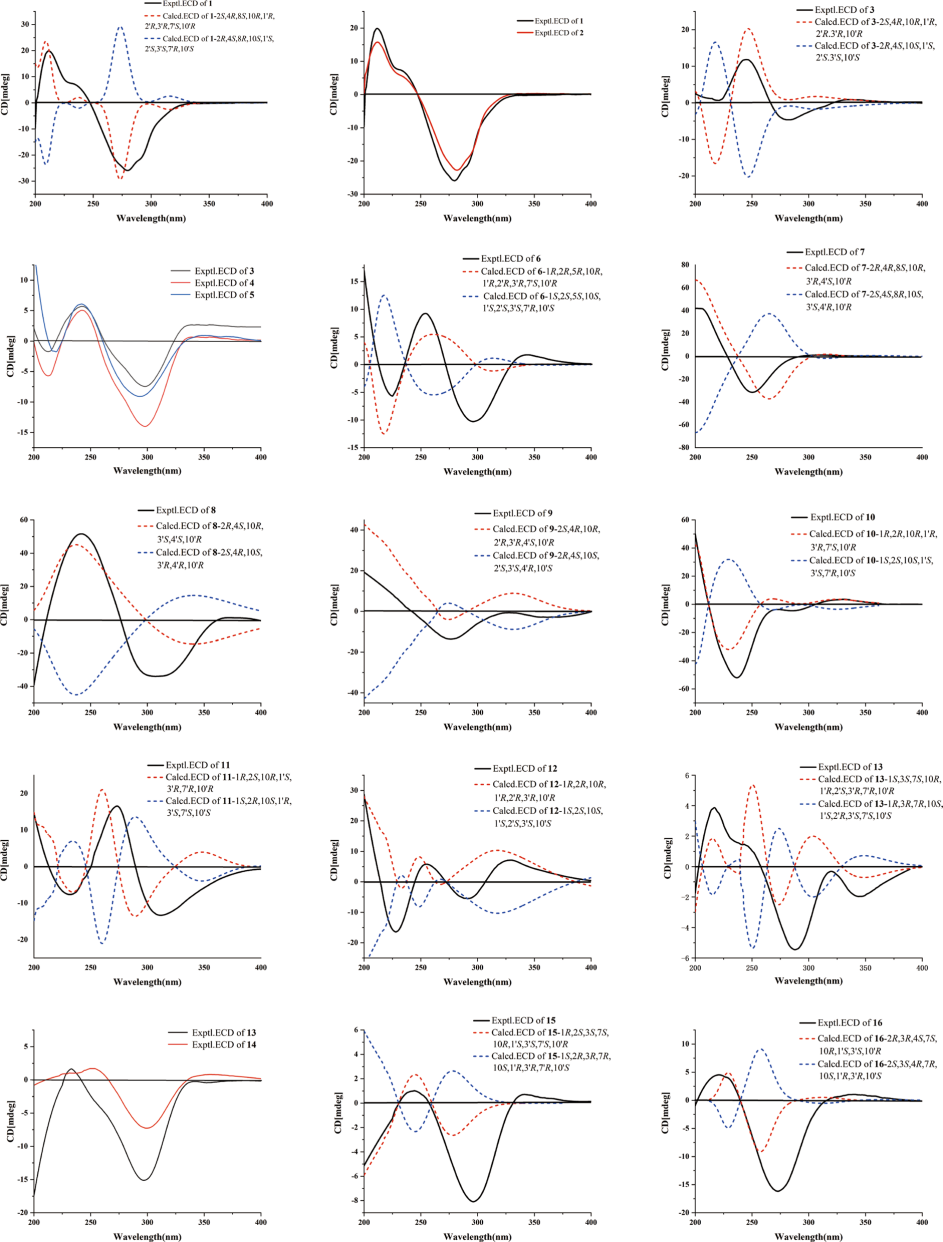
Calculated and experimental ECD spectra of **1–16**

Xylanin B (**2**) was obtained as colorless crystals. The molecular formula of **2** was found to be C_31_H_44_O_4_ (HR-ESI–MS [M + Na]^+^ at *m/z* 503.3130; calcd 503.3132), requiring 10 degrees of unsaturation. The ^13^C NMR and DEPT data (Table 1) of **2** exhibited 31 carbons signals that were almost identical to those of **1**. The only difference found between **1** and **2** was the replacement of a hydroxyl group at C-8 by a methoxy moiety (Fig. 1). The HMBC cross-peak from H_3_-1′′ [*δ*_H_ 3.36 (3H, s), *δ*_C_ 49.3] to C-8 (*δ*_C_ 106.1) suggested that the methoxy was assigned to C-8 (Fig. 3). The NOESY correlations of H-2/H_3_-14′, H-2′/H-10′, H_3_-14/H-2′ and H-10′/H-7′ indicated that the relative configurations of these chiral centers were identical to those of **1**. Furthermore, the NOESY correlation of H_3_-1′′/H-10 and H-1′/ H_3_-1′′ indicated that H-1′, H_3_-1′′ and H-10 were *α*-oriented. The correlation of H-3′/H_3_-14 suggested that H-3′ was *β*-oriented. Consequently, the absolute configuration of **2** was the same as that of **1** owing to the similar ECD effects (Fig. 5).

Xylanin C (**3**) was obtained as yellow oil. The molecular formula of **3** was found to be C_30_H_40_O_2_ (HRESIMS [M + H]^+^ at *m/z* 433.3101; calcd 433.3101), indicating 11 degrees of unsaturation. The comparison of the 1D NMR and 2D NMR data (Table 1) showed that **3** had the same skeleton as **1**. The primary differences observed were the absence of a five-membered ring (III) at unit A and the olefinic bond at C-6/C-7 in** 3**, which were supported by the changes of chemical shifts at C-6 (*δ*_C_ 25.9), C-7 (*δ*_C_ 135.4), C-8 (*δ*_C_ 204.7), and C-11 (*δ*_C_ 139.7) (Table 1). The analysis of the NMR data revealed that **3** possessed two additional double bonds compared to **1**. These were unambiguously assigned to the positions C-7 (*δ*_C_ 135.4)/C-11 (*δ*_C_ 139.7) and C-7′ (*δ*_C_ 133.8)/C-11′ (*δ*_C_ 139.2), based on a detailed comparison of their respective chemical shifts. The key NOESY correlations of H-2/H_3_-14′, H-1′/H_3_-14′ and H-1′/H-10 indicated that these protons shared the same spatial orientation and were assigned as *α*-oriented. Additionally, the correlations of H-2′/H_3_-14 and H-3′/H-10′ suggested that H-2′, H-3′ and H-10′ were *β*-oriented (Fig. 4). Furthermore, experimental and calculated ECD results were highly similar (Fig. 5). Above of all, the absolute configuration for **3** was determined as 2*S*, 4*R*, 10*R*, 1′*R*, 2′*R*, 3′*R*, 10′*R*.

Xylanin D (**4**) was obtained as colorless oil. Its molecular formula C_30_H_44_O_2_ was established by HR-ESI–MS: *m/z* 437.3417 [M + H] ^+^ (calcd for 437.3414), containing 9 degrees of unsaturation. The comparison of the 1D NMR and 2D NMR data (Table 1) indicated that **4** possessed the same structural framework as **3**. The main differences were the absence of olefinic bonds at C-7/C-11 and C-7′/C-11′ in **4**, and the chemical shifts of C-7 (*δ*_C_ 58.4), C-11 (*δ*_C_ 30.6), C-7′ (*δ*_C_ 58.9) and C-11′ (*δ*_C_ 31.8) shifted to higher field values (Table 1). The relative configuration of **4** matched that of **3**, as supported by their identical NOESY cross-peaks. The relative configurations of H-2, H-1′, H-7 and H-10 were assigned as *α-*orientation due to the NOESY cross-peaks of H-2/H-1′ and H-7/H-10. In addition, the NOESY correlation between H-10′ and H-7′ indicated that H-7′ was *β*-oriented. Consequently, the absolute configuration of **4** was assigned as 2*S*, 4*R*, 7*S*, 10*R*, 1′*R*, 2′*R*, 3′*R*, 7′*S*, 10′*R* owing to the similar ECD spectra of **4** and **3** (Fig. 5).

Xylanin E (**5**), obtained as white powder. The molecular formula of **5** was found to be C_30_H_42_O_2_ (HR-ESI–MS [M + H]^+^ at *m/z* 435.3254; calcd 435.3258), requiring 10 degrees of unsaturation. The 1D and 2D NMR data (Table 2) of **5** exhibited a high degree of similarity to those of **4** except for the appearance of a double bond at C-7′ (*δ*_C_ 133.1)/C-11′ (*δ*_C_ 140.2). The relative configuration of **5** was identical to **4** by analyzing their NOESY interactions. Furthermore, the small coupling constant between H-2′ and H-3′ (*J*_*3′*_ = 2.1 Hz) indicated that they adopted a coaxial configuration, thereby assigning the *β*-orientation to H-2′ and H-3′. Additionally, the observed NOESY correlations of H-2′/H_3_-14, H-3′/H-10′ and H-7/H-10 allowed for the assignment of a *β*-orientation of H_3_-14 and H-10′, and an *α*-orientation of H-7 (Fig. 4). The similar ECD spectra of **5** and **4** indicated that the absolute configuration of **5** was identical to **4** (Fig. 5). Accordingly, the structure of **5** was defined as 2*S*, 4*R*, 7*S*, 10*R*, 1′*R*, 2′*R*, 3′*R*, 10′*R*.

**Table 2:** ^1^H (400 MHz) and ^13^C (100 MHz) NMR data for compounds **5**—**8** in CDCl_3_

Position	**5**		**6**		**7**		**8**	
	*δ*_H_ (*J* in Hz)	*δ*_C_	*δ*_H_ (*J* in Hz)	*δ*_C_	*δ*_H_ (*J* in Hz)	*δ*_C_	*δ*_H_ (*J* in Hz)	*δ*_C_
1		143.4	1.27–1.29 m	73.1		150.9		140.6
2	2.28 d (4.8)	51.4	2.14–2.17 m	49.6	2.68 s	52.2	2.09 s	57.3
3a	1.23 dd (1.5, 8.8)	56.8	5.40 s	126.4	1.92 dd (1.0, 6.0)	56.9		199.8
3b	1.23 dd (1.5, 8.8)				1.66 dd (1.2, 5.7)			
4		5.1		142.9		56.1		59.8
5		138.4		61.0		132.3		135.7
6a	2.32–2.41 m	23.1	3.25 d (16.3)	30.8	5.47 s	112.1	3.25 d (19.3)	25.7
6b	2.03 t (4.4)		2.56–2.61 m^a^				3.01 d (17.3)	
7	1.98–2.01 m	58.3		134.9		153.6		132.0
8		214.6		206.2		103.2		202.1
9a	2.81 t (11.2)	48.0	2.40 t (11.1)	50.9	1.94–1.96 m	37.3	2.92–2.95 m	48.7
9b	2.26 d (4.8)		2.20 dd (2.8, 8.5)		1.57 d (10.4)		2.31 dd (4.0, 10.1)	
10	2.45–2.52 m	35.5	1.77–1.79 m	30.2	2.57–2.63 m	30.9	2.27–2.32 m	34.9
11	1.93 t (6.6)	30.4		140.5		85.5		146.9
12	0.91 d (3.9)	19.2	1.87 s	22.7	1.41 s	25.1	1.89 s	23.8
13	0.89 d (3.9)	20.7	1.94 s	23.3	1.35 s	28.0	2.05 s	23.9
14	1.14 d (6.5)	20.9	0.91–0.94 m^a^	22.6	1.21 d (5.8)	19.5	1.13 d (6.8)	19.9
15	1.29 s	18.1	1.46 s	14.8	1.50 s	18.2	1.49 s	11.4
1′	1.51–1.54 m	58.1	1.51–1.54 m	59.1		145.4		152.2
2′	2.56–2.59 m	46.7	2.84 dd (1.7, 6.2)	64.2		206.7		203.6
3′	2.64 d (2.1)	61.9	2.56–2.61 m^a^	48.7	2.15 s	63.2	2.87 s	57.2
4′		135.6		134.7		55.7		49.7
5′		138.1		139.3		171.5		159.2
6a′	3.33 d (15.5)	28.1	2.49 dd (5.0, 8.5)	26.3	3.42 d (13.8)	28.6	6.61 s	122.4
6b′	2.66–2.69 m		1.81–1.84 m^a^		3.09 d (14.0)			
7′		133.1	1.98–2.01 m	58.7		132.2		155.7
8′		105.8		216.3		205.1		203.7
9a′	2.70–2.73 m	50.7	2.64 d (11.4)	48.4	3.21 dd (2.7, 8.9)	46.8	2.88–2.92 m	45.4
9b′	2.17 dd (1.8, 9.7)		2.04 dd (1.8, 9.0)		2.33 dd (4.2, 7.6)		2.54 dd (4.6, 8.4)	
10′	1.33–1.37 m	40.9	1.30–1.33 m	41.6	2.45–2.50 m	27.3	2.73–2.79 m	26.2
11′		140.2	1.81–1.84 m^a^	31.8		143.9		73.6
12′	1.97 d (1.9)	22.8	0.91—0.94 m^a^	19.5	1.88 s	23.0	1.48 s	29.4
13′	1.88 d (0.8)	22.4	0.89 d (6.8)	20.9	2.0 s	23.6	1.51 s	30.1
14′	1.01 d (6.5)	21.7	1.04 d (6.5)	22.0	0.90 d (5.7)	18.8	0.90 d (7.2)	18.0
15′	1.50 s	14.0	1.67 s	14.7	1.53 s	24.1	1.35 s	21.8
-OH							3.46 s	

^a^Overlapped signals

Xylanin F (**6**) was obtained as yellow powder. Its molecular formula was deduced as C_30_H_42_O_2_ from HR-ESI–MS *m/z* 435.3264 [M + H] ^+^ (calcd. for 435.3258), accounting 10 degrees of unsaturation. The comparison of the 1D NMR and 2D NMR data (Table 2) revealed that the structure of **6** was similar to that of **4**, with the exception of the presence of a double bond at C-7 (*δ*_C_ 134.9)/C-11 (*δ*_C_ 140.5). Additionally, modifications were made to the linkage between unit A and unit B, and the position of the double bond in unit A was adjusted. The ^1^H-^1^H COSY correlations from H-2′ to H-2/H-1′ and the crucial HMBC cross-peaks from H-2 to C-1′, and from H-3′ to C-4/C-5 (Fig. 3) indicated that unit A and unit B were linked by two direct C–C bonds (C-2 to C-2′ and C-5 to C-3′). The HMBC correlations from H-1 to C-3 (*δ*_C_ 126.4)/C-4 (*δ*_C_ 142.9), from H-2′ to C-4 (*δ*_C_ 142.9), from H-3′ to C-4 (*δ*_C_ 142.9), and from H_3_-15 to C-3 (*δ*_C_ 126.4)/C-4 (*δ*_C_ 142.9) suggested that C-3/C-4 were a double bond in unit A (Fig. 3). The NOESY cross-peaks of H-1/H-2′, H-1/H_3_-14, H-3′/H-1, H-2/H-3′, H-10′/H-3′ and H-1′/H_3_-14′ were used to assign a *β*-orientation of H-1, H-2′, H_3_-14 H-3′, H-2 and H-10′, and an *α*-orientation of H-1′ (Fig. 4). Furthermore, the NOESY correlation between H-11′ and H-1′ confirmed the *α-*orientation of H-11′, which consequently required the *β*-orientation of H-7′. By comparing its experimental and calculated ECD spectra (Fig. 5), the absolute configuration of **6** was determined to be 1*R*, 2*R*, 5*R*, 10*R*, 1′*R*, 2′*R*, 3′*R*, 7′*S*, 10′*R*.

Xylanin G (**7**) was obtained as yellow oil. Its molecular formula C_30_H_38_O_5_ was established by HR-ESI–MS: *m/z* 501.2675 [M + Na] ^+^ (calcd for 501.2670), containing 12 degrees of unsaturation. The 1D NMR and 2D NMR data (Table 2) of **7** closely matched those of **1**, with the exception of the significant downfield shifts observed for C-2′ (*δ*_C_ 206.7), C-7′ (*δ*_C_ 132.2), C-11′ (*δ*_C_ 143.9), C-1′ (*δ*_C_ 145.4) and C-5′ (*δ*_C_ 171.5). Based on the aforementioned information, a carbonyl group was identified at C-2′, a double bond was observed between C-7′ and C-11′, and the position of the double bond in unit B was altered. The key HMBC cross-peaks from H-3′ to C-5′ (*δ*_C_ 171.5), from H_2_-6′ to C-1′ (*δ*_C_ 145.4)/C-5′ (*δ*_C_ 171.5), and from H_2_-9 to C-1′ (*δ*_C_ 145.4) confirmed the presence of a double bond between C-1′ and C-5′ in unit B (Fig. 3). Furthermore, the connection between unit A and unit B was different. The key HMBC cross-peaks from H-2 to C-3′/C-4′, from H_2_-3 to C-4/C-3′, from H_3_-15 to C-4/C-3′, and from H_3_-15′ to C-2 were used to infer that unit A and unit B were linked by two direct C–C bonds (C-2 to C-4′ and C-4 to C-3′). The key NOESY signals (Fig. 4) of H-2/H-10 and H-10′/H-2 confirmed that these protons were co-facial and were assigned as *α*-oriented. The relative configurations of H-3′ and H_3_-15′ were assigned as *β-*orientation due to the NOESY cross-peaks of H-3′/H_3_-15′ and H-3′/H_3_-14′. Thus, the absolute configuration of **7** was defined as 2*R*, 4*R*, 8*S*, 10*R*, 3′*R*, 4′*S*, 10′*R* for the calculated ECD spectrum matched well with the experimental result.

Xylanin H (**8**) was obtained as colorless oil. Its molecular formula was determined to be C_30_H_36_O_5_ based on the positive HR-ESI–MS at *m/z* 477.2625 [M + H] ^+^ (calcd for 477.2636), requiring 13 degrees of unsaturation. Its NMR data (Table 2) resembled that of **7**, with the presence of additional signals attributable to two carbonyl groups (*δ*_C_ 199.8 and 202.1) and a hydroxyl group [*δ*_H_ 3.46 (1H, s)]. Notably absent were the signals characteristic of a five-membered ring (III). The HMBC cross-peaks from H_3_-15 to C-3 (*δ*_C_ 199.8), from H-10 to C-8 (*δ*_C_ 202.1), from H_2_-6 to C-8 (*δ*_C_ 202.1), from H_3_-12′ to C-11′ (*δ*_C_ 73.6), and from H_3_-13′ to C-11′ (*δ*_C_ 73.6) revealed that the carbonyl groups were located at C-3 and C-8, and that there was a hydroxyl group at C-11′ (Fig. 3). In the NOESY spectrum, the *β*-orientation of H-3′, H_3_-14, H_3_-14′ and H_3_-15′ was determined by the key correlations of H-3′/H_3_-14, H-3′/H_3_-14′ and H_3_-15′/H-3′. Meanwhile, the *α*-orientation of H-2 was determined by the key correlation of H-2/H-10 (Fig. 4). Analyses of its experimental and calculated ECD spectra (Fig. 5) suggested that the absolute configuration of **8** was defined as 2*R*, 4*S*, 10*R*, 3′*S*, 4′*S*, 10′*R*.

Xylanin I (**9**) was obtained as white powder. Its molecular formula C_30_H_40_O_2_ was established by HR-ESI–MS: *m/z* 433.3103 [M + H] ^+^ (calcd for 433.3101), containing 11 degrees of unsaturation. The 1D and 2D NMR data (Table 3) of **9** were similar to those of** 8**, except for the absence of two carbonyls at C-3 (*δ*_C_ 52.3) and C-2′ (*δ*_C_ 58.7) and a shift in the position of a double bond in unit B. The significant HMBC correlations from H_2_-6′ to C-7′ (*δ*_C_ 134.0), from H_3_-12′ to C-7′ (*δ*_C_ 134.0)/C-11′ (*δ*_C_ 143.9), and from H_3_-13′ to C-7′ (*δ*_C_ 134.0)/C-11′ (*δ*_C_ 143.9) confirmed that C-7′/C-11′ were a double bond in unit B (Fig. 3). The two units were linked via direct C–C bonds (C-2 to C-3′ and C-4 to C-2′), as indicated by the key HMBC correlations from H_2_-3 to C-2′/C-3′, and from H-2′ to C-4 (Fig. 3). The *α*-orientation of H-2, H-2′, H-10, H-10′, H-3′ and H_3_-15′ was indicated by the NOESY cross-peaks of H-2/H-2′, H-10/H-2, H-2′/H-10′ and H-3′/H_3_-15′ (Fig. 4). The absolute configurations of **9** (2*S*, 4*R*, 10*R*, 2′*R*, 3′*R*, 4′*S*, 10′*R*) were finally determined by a similar ECD experiment (Fig. 5).

**Table 3:** ^1^H (400 MHz) and ^13^C (100 MHz) NMR data for compounds **9**—**12** in CDCl_3_

Position	**9**		**10**		**11**		**12**	
	*δ*_H_ (*J* in Hz)	*δ*_C_	*δ*_H_ (*J* in Hz)	*δ*_C_	*δ*_H_ (*J* in Hz)	*δ*_C_	*δ*_H_ (*J* in Hz)	*δ*_C_
1		143.7		63.3		65.2		58.4
2	2.52–2.55 m	46.4	3.22 s	60.6	2.47 s	55.6	2.63 s	54.0
3a	1.25–1.27 m	57.3		199.4		209.1		206.4
3b	1.18 dd (1.2, 7.7)							
4		56.8		134.7		138.4		161.3
5		138.4		134.9		169.0		145.6
6a	3.02–3.07 m	27.8	3.13 d (16.0)	26.3	3.62 d (11.2)	30.9	7.06 s	126.3
6b	2.55–2.60 m		2.70–2.75 m		2.91 d (11.0)			
7		135.7		132.4		130.1		153.0
8		205.3		209.0		203.9		206.1
9a	2.72–2.77 m	47.7	2.88–2.91 m	49.5	3.25 dd (1.3, 10.9)	47.6	3.24 dd (6.4, 7.8)	51.6
9b	2.35–2.39 m		2.44 d (13.2)		2.52 dd (5.6, 6.6)		2.59 s (5.0)	
10	2.39–2.42 m	34.7	2.91–2.94 m	27.0	2.26–2.30 m	34.9	2.47–2.51 m	34.5
11		139.8		133.7		146.0		73.5
12	1.95 s	22.9	1.65–1.68 m^a^	20.2	1.95 s	23.8	1.47 s	29.9
13	1.77 s	22.4	1.65–1.68 m^a^	21.8	2.03 s	23.9	1.50 s	29.5
14	1.02 d (6.5)	19.5	1.27 d (6.3)	16.2	0.86 d (5.7)	16.3	0.97 d (6.9)	18.8
15	1.36 s	18.6	1.65–1.68 m^a^	14.0	1.61 s	8.7	1.79 s	8.9
1′		139.5		58.2		65.0		64.2
2a′	2.98–3.02 m	58.7		203.8	1.63–1.65 m	48.1	4.94 s	85.2
2b′					1.56–1.57 m			
3′	2.26–2.31 m	52.5	2.62–2.66 m^a^	51.0	2.77 s	52.4	2.86 s	57.4
4′	1.97–2.01 m	45.7		145.7		135.8		132.3
5′		140.5		168.5		141.9		135.7
6a′	3.07–3.13 m	27.8	2.62–2.66 m^a^	30.5	2.16–2.20 m	31.8	2.99–3.04 m	25.7
6b′	2.94 d (16.6)		2.25–2.28 m		1.57–1.59 m		2.52–2.58 m	
7′		134.0	2.66–2.70 m	55.5	1.71–1.75 m	50.3		133.3
8a′		203.5		210.9	1.76–1.80 m	36.8		208.4
8b′					1.42–1.46 m			
9a′	2.68–2.72 m	48.5	2.83–2.88 m	50.0	1.50–1.53 m	34.3	2.43 dd (4.6, 12.1)	51.2
9b′	2.63 dd (4.4, 7.2)		2.34 d (6.0)		1.31–1.33 m		2.43 dd (4.6, 12.1)	
10′	2.44–2.50 m	33.3	2.28–2.31 m	33.6	2.34–2.38 m	33.8	3.04–3.08 m	27.4
11′		143.9	2.03–2.07 m	29.0		151.6		133.8
12′	2.05 s	23.7	0.98 d (6.7)	19.8	4.62 d (12.3)	108.3	1.68 s	21.7
13′	1.82 s	23.8	0.91 d (6.4)	21.7	1.68 s	20.7	1.67 s	20.3
14′	1.12 d (7.0)	20.4	0.81 d (6.6)	17.0	0.97 d (5.6)	18.4	1.14 d (6.8)	16.3
15′	0.98 d (7.0)	21.9	1.62 s	8.7	1.54 s	13.7	1.42 s	14.0
-OH							3.45 s	
-COOCH_3_								171.2
-COOCH_3_							2.02 s	21.4

^a^Overlapped signals

Xylanin J (**10**) was obtained as white powder. The molecular formula of **10** was found to be C_30_H_38_O_4_ (HR-ESI–MS [M + H]^+^ at *m/z* 463.2840; calcd 463.2843), requiring 12 degrees of unsaturation. The ^13^C NMR spectrum (Table 3), interpreted with the assistance of the HSQC spectrum, revealed the presence of 30 carbon atoms within 6 different groups, consisting of eight methyls, four methylenes, six methines, four carbonyls, six olefins, and two quaternary carbons. The 1D NMR data (Table 3) exhibited two guaiane units (A and B) in the structure of **10**, which was further confirmed by 2D NMR experiments (Fig. 3). In unit A, the HMBC cross-peaks from H-2 to C-1/C-3/C-4/C-5, and from H_3_-15 to C-4/C-5 suggested the appearance of a five-membered ring (I). Moreover, a seven-membered ring (II) was fused with five-membered ring (I) at C-1 and C-5 by analyses of the HMBC cross-peaks from H_2_-6 to C-1/C-4/C-5/C-8, from H_2_-9 to C-1/C-8/C-10/C-14, and from H_3_-14 to C-1/C-9/C-10. Following the analysis of the above spectra, it was determined that the unit A was to be designated a guaiane unit. Similarly, unit B was also assigned as a guaiane unit. The linkage of unit A and unit B via two direct C–C bonds (C-1 to C-3′ and C-2 to C-1′) was deduced from the key HMBC cross-peaks from H-2 to C-3′/C-10′, and from H-3′ to C-1/C-5/C-10 (Fig. 3). The key NOESY correlations of H-3′/H-10, H-7′/H-10′ and H-10′/H-3′ indicated that these protons shared the same spatial orientation and were assigned as *α*-oriented. The correlation of H-2/H_3_-14′ revealed that H-2 was *β*-oriented (Fig. 4). The absolute configuration of **10** was defined as 1*R*, 2*R*, 10*R*, 1′*R*, 3′*R*, 7′*S*, 10′*R* by a similar ECD experiment (Fig. 5).

Xylanin K (**11**) was obtained as yellow oil. Its molecular formula C_30_H_40_O_2_ was established by HR-ESI–MS: *m/z* 455.2916 [M + Na] ^+^ (calcd for 455.2921), containing 11 degrees of unsaturation. Comparison of the ^13^C NMR and DEPT data (Table 3) revealed that **11**, in contrast to **10**, lacked two carbonyl groups at C-2′ (*δ*_C_ 48.1)/C-8′ (*δ*_C_ 36.8), and exhibited an additional double bond at C-11′ (*δ*_C_ 151.6)/C-12′ (*δ*_C_ 108.3). These differences were further supported by the HMBC correlations from H_2_-2′ to C-2/C-4′/C-5′, from H_2_-8′ to C-10′, and from H_2_-12′ to C-7′/C-13′. In the NOESY spectrum of **11**, the key NOESY correlations of H-2/H_3_-14, H-2/H_3_-14′ and H-7′/H_3_-14′ indicated that H-2, H_3_-14, H_3_-14′ and H-7′ were *β*-oriented. The correlation of H-3′/H-10 suggested that H-3′ and H-10 were *α*-oriented (Fig. 4). Furthermore, the calculated ECD spectrum of **11** (Fig. 5) was similar with the experimental data closely. Therefore, the absolute configuration of **11** was assigned as 1*R*, 2*S*, 10*R*, 1′*S*, 3′*R*, 7′*R*, 10′*R*.

Xylanin L (**12**) was obtained as colorless oil. Its molecular formula C_32_H_40_O_6_ was established by HR-ESI–MS: *m/z* 543.2720 [M + Na] ^+^ (calcd for 543.2717), requiring 13 degrees of unsaturation. The comparison of the 1D NMR and 2D NMR data (Table 3) revealed that **12** shared a similar skeletal structure with **10**, with the exception of several significant differences. These included the presence of a hydroxyl group [*δ*_H_ 3.45 (1H, s)], an acetoxy group [*δ*_H_ 2.02 (3H, s), *δ*_C_ 21.4, -OOCCH_3_, *δ*_C_ 171.2, -OOCCH_3_], and a double bond at C-7′ (*δ*_C_ 133.3)/C-11′ (*δ*_C_ 133.8), as well as a shift in the position of a double bond in unit A. The chemical shift of C-11 (*δ*c 73.5) was consistent with the presence of a hydroxyl group. The key HMBC correlations from H-2′ [*δ*_H_ 4.94 (1H, s)] to C-1′′ (*δ*_C_ 171.2, -OOCCH_3_), and from H-2′′ [*δ*_H_ 2.02 (3H, s)] to C-1′′ (*δ*_C_ 171.2, -OOCCH_3_), along with the chemical shift of C-2′ (*δ*c 85.2), implied that C-2′ was connected to an acetoxy group. The key HMBC correlations from H_3_-12′ to C-7′ (*δ*c 133.3)/C-11′ (*δ*c 133.8), from H_3_-13′ to C-7′ (*δ*c 133.3)/C-11′ (*δ*c 133.8), from H-6 to C-7(*δ*c 153.0), and from H_2_-9 to C-7(*δ*c 153.0) could be speculated to that C-7′/C-11′ and C-6/C-7 were double bonds (Fig. 3). The linkage of unit A and unit B via two direct C–C bonds (C-1 to C-3′ and C-2 to C-1′) was similar to **10**. Furthermore, the key NOESY signals (Fig. 4) of H-2/H_3_-14, H-2/H_3_-14′, H-2′/H-2 and H-2′/H_3_-14′ confirmed that these protons were co-facial and were assigned as *β*-oriented. The correlation of H-3′/H-10 indicated that H-3′ and H-10 were *α*-oriented. The absolute configuration of **12** (1*R*, 2*R*, 10*R*, 1′*R*, 2′*R*, 3′*R*, 10′*R*) was identified from the similarity between the experimental and calculated ECD spectra (Fig. 5).

Xylanin M (**13**) was obtained as white amorphous powder. Its molecular formula was established to be C_30_H_44_O_2_ by HR-ESI–MS (*m/z* 437.3404 [M + H] ^+^; calcd 437.3414), corresponding to an index of hydrogen deficiency of 9. The 1D NMR data (Table 4) indicated the presence of two guaiane units (A and B) in the structure of **13**, which was further confirmed by 2D NMR experiments. In unit A, the key HMBC cross-peaks from H_3_-15 to C-3/C-4/C-5, and from H_2_-2 to C-1/C-3/C-4/C-5 established the presence of a five-membered ring (I). The presence of a seven-membered ring (II) fused with a five-membered ring (I) in unit A at C-1 and C-5 was revealed by the key HMBC cross-peaks from H_2_-6 to C-1/C-4/C-5/C-8, from H_2_-9 to C-1/C-8/C-10/C-14, and from H_3_-14 to C-1/C-8/C-9/C-10. As with unit A, unit B was also identified as a guaiane sesquiterpene with the aid of key HMBC cross-peaks. The ^1^H-^1^H COSY correlations from H-3′ to H-3/H-2′ and the crucial HMBC cross-peaks from H-3 to C-2′, from H-1′ to C-1, and from H-2′ to C-1/C-10 (Fig. 3) indicated that unit A and unit B were linked by two direct C–C bonds (C-3 to C-3′ and C-1 to C-2′). In the NOESY spectrum, H-3, H-2′, H-3′, H-10′ and H_3_-14 were determined to be *α*-oriented by the key correlations of H-3/H-2′, H-2′/H_3_-14, H-3′/H-10′ and H-2′/H-10′, while the *β*-orientation of H-7, H-10, H-1′, H_3_-14′ and H-7′ was determined by the key cross-peaks of H-7/H-10, H-1′/H_3_-14′ and H-7′/H-1′ (Fig. 4). The absolute configuration of **13** was established via ECD calculation which closely matched the experimental data (Fig. 5), allowing the assignment of the absolute configuration as 1*S*, 3*S*, 7*S*, 10*R*, 1′*R*, 2′*S*, 3′*R*, 7′*R*, 10′*R*.

**Table 4:** ^1^H (400 MHz) and ^13^C (100 MHz) NMR data for compounds **13**—**16** in CDCl_3_

Position	**13**		**14**		**15**		**16**	
	*δ*_H_ (*J* in Hz)	*δ*_C_	*δ*_H_ (*J* in Hz)	*δ*_C_	*δ*_H_ (*J* in Hz)	*δ*_C_	*δ*_H_ (*J* in Hz)	*δ*_C_
1		63.5		63.7	2.09–2.14 m	57.9		138.7
2a	1.36 dd (1.8, 6.1)	46.4	1.46 dd (0.6, 7.0)	55.8	2.26–2.30 m	52.5	2.26–2.30 m^a^	52.8
2b	1.05–1.08 m		1.16 dd (1.7, 6.0)	49.2				
3	2.69–2.73 m	49.6	2.45—2.47 m		2.44–2.47 m	49.4	3.28 d (8.4)	53.0
4		133.7		134.6		137.2	2.03–2.06 m	46.6
5		140.7		137.7		138.3		143.8
6a	2.55–2.60 m	27.3	3.30 d (15.3)	28.0	2.47—2.52 m^a^	24.7	2.38–2.43 m	25.5
6b	1.95–1.98 m		2.35 d (15.4)		1.58–1.61 m		1.88 dd (3.9, 11.6)	
7	2.23–2.28 m	59.4		133.0	1.64–1.68 m	58.0	1.99–2.02 m	59.5
8		215.9		206.6		216.0		214.5
9a	2.48–2.54 m	48.1	3.21–3.27 m	49.8	2.66 t (11.4)	48.5	2.84 t (10.6)	47.4
9b	2.04–2.10 m^a^		2.26 dd (1.4, 14)		2.05–2.09 m		2.30–2.37 m	
10	2.18–2.22 m	30.6	2.11–2.16 m	34.0	1.26–1.31 m	42.0	2.66–2.75 m	34.3
11	1.81–1.86 m	27.6		142.1	2.01–2.05 m	30.1	1.92 d (6.9)	30.8
12	0.94 d (6.6)	20.8	1.79–1.81 m^a^	22.2	0.90–0.95 m^a^	19.1	0.88 d (3.5)	19.2
13	0.79 d (6.6)	21.4	1.96 s	23.0	0.90–0.95 m^a^	19.5	0.89 d (3.5)	20.8
14	1.03 d (6.8)	18.0	1.10 d (7.2)	19.7	1.06 d (6.5)	22.0	1.15 d (6.6)	22.0
15	1.52 s	14.7	1.79–1.81 m^a^	15.9	1.72 s	15.4	0.94 d (6.9)	22.2
1′	1.54–1.58 m	58.0	1.69–1.73 m	57.9		63.2		64.2
2′	2.04–2.10 m^a^	48.9	2.28–2.32 m	52.7	1.40–1.45 m	55.9	1.40–1.43 m	56.3
					1.19 dd (1.7, 6.2)		1.12–1.13 m	
3′	3.07–3.13 m	58.8	3.13 d (7.4)	55.1	3.11 d (7.0)	55.7	2.46–2.50 m	48.0
4′		136.2		134.6		134.9		134.2
5′		136.6		140.0		139.5		138.2
6a′	2.44–2.48 m	26.7	2.48 dd (5.1, 8.7)	26.8	2.47–2.52 m^a^	26.8	3.22 d (15.3)	28.2
6b′	1.73–1.77 m		1.82–1.86 m		1.79–1.83 m		2.26–2.30 m^a^	
7′	1.98–2.02 m	59.2	2.00–2.05 m	58.1	1.97–2.01 m	58.5		132.8
8′		216.1		216.0		215.6		206.0
9a′	2.64 d (11.3)	48.3	2.67 t (11.4)	48.5	2.99 t (12.7)	49.1	3.14 dd (2.8, 12.4)	50.1
9b′	2.04–2.10 m^a^		2.08 dd (1.8, 9.1)		2.22–2.26 m		2.21–2.25 m	
10′	1.21–1.26 m	42.2	1.26–1.30 m	42.0	2.16–2.22 m	35.5	2.08–2.14 m	34.0
11′	1.79 s	31.8	1.86–1.90 m	31.8	1.84–1.89 m	31.8		142.9
12′	0.92 d (6.8)	19.7	0.93 d (7.1)	19.4	0.90–0.95 m^a^	20.9	1.96 s	23.1
13′	0.87 d (6.8)	21.0	0.91 d (7.1)	20.9	0.90–0.95 m^a^	21.0	1.79 s	22.4
14′	1.00 d (6.6)	22.1	1.07 d (6.5)	22.1	1.14 d (7.2)	19.9	1.09 d (7.2)	19.7
15′	1.50 s	13.4	1.68 s	15.7	1.63 s	15.7	1.76 s	15.5

^a^Overlapped signals

Xylanin N (**14**) was obtained as white powder. The molecular formula of **14** was found to be C_30_H_42_O_2_ (HR-ESI–MS [M + Na] ^+^ at *m/z* 457.3077; calcd 457.3077), requiring 10 degrees of unsaturation. The 1D and 2D NMR data (Table 4) of **14** were similar to those of **13**, with the exception of the appearance of a double bond at C-7 (*δ*c 133.0)/C-11 (*δ*c 142.1). The key HMBC correlations from H_2_-6 to C-7/C-11, from H_3_-12 to C-7/C-11, and from H_3_-13 to C-7/C-11 verified the aforementioned conclusion. The relative configuration of **14** was determined by analyzing its NOESY data. The key NOESY cross-peak of H-1′/H_3_-14′ indicated that H-1′ was *β*-oriented. In contrast, the key NOESY cross-peaks of H-2′/H-3, H-2′/H-10′, H-3′/H_3_-14 and H-7′/H-10′ suggested that H-2′, H-3, H-10′, H-3′ and H-7′ were *α*-oriented. Consequently, the ECD spectrum of **14** was in accordance with that of **13**. Therefore, the absolute configuration of **14** was established as 1*S*, 3*S*, 10*R*, 1′*R*, 2′*S*, 3′*R*, 7′*S*, 10′*R*.

Xylanin O (**15**) was obtained as white powder. Its molecular formula C_30_H_44_O_2_ was established by HR-ESI–MS: *m/z* 459.3225 [M + Na] ^+^ (calcd for 459.3234), containing 9 degrees of unsaturation. The comparison of the 1D NMR and 2D NMR data (Table 4) revealed that **15** closely resembled **14**, with the exception of the absence of a double bond at C-7 (*δ*c 58.0)/C-11 (*δ*c 30.1) and the alteration in the linkage between unit A and unit B. The linkage between unit A and unit B via two direct C–C bonds (C-2 to C-3′ and C-3 to C-1′) was due to the key ^1^H-^1^H COSY correlations from H-2 to H-3/H-3′, and the key HMBC correlations from H-3 to C-1′, and from H_2_-2′ to C-2/C-3′. The NOESY correlations of H-1/H_3_-14, H-2/H_3_-14, H-3/H_3_-14′ and H-3′/H_3_-14′ indicated the *α*-orientation of H-1, H-2, H-3 and H-3′. Similarly, the *α*-orientation of H-7 and H-7′ was defined by the correlations of H-7/H-10 and H-10′/H-7′. The overall pattern of the experimental spectrum was in reasonable agreement with the calculated ECD spectrum of **15** (Fig. 5), which indicated the 1*R*, 2*S*, 3*S*, 7*S*, 10*R*, 1′*S*, 3′*S*, 7′*S*, 10′*R* absolute configuration of **15**.

Xylanin P (**16**) was obtained as yellow powder. The molecular formula of **16** was found to be C_30_H_42_O_2_ (HR-ESI–MS [M + Na] ^+^ at *m/z* 457.3076 calcd for 457.3077), accounting 10 degrees of unsaturation. The comparison of the 1D NMR and 2D NMR data (Table 4) revealed that the structure of **16** was similar to that of **15**, except for the presence of a double bond at C-7′/C-11′ and a shift in the location of a double bond within unit A. The HMBC correlations from H_2_-6′ to C-7′ (*δ*_C_ 132.8)/C-11′ (*δ*_C_ 142.9), from H_3_-12′ to C-7′ (*δ*_C_ 132.8)/C-11′ (*δ*_C_ 142.9), from H_3_-13′ to C-7′ (*δ*_C_ 132.8)/C-11′ (*δ*_C_ 142.9), from H_3_-15 to C-5 (*δ*_C_ 143.8), from H-2 to C-1 (*δ*_C_ 138.7), from H-3 to C-5 (*δ*_C_ 143.8), and from H_2_-6 to C-1 (*δ*_C_ 138.7) could be speculated that C-7′/C-11′ and C-1/C-5 were double bonds (Fig. 3). The key NOESY signals (Fig. 4) of H-3/H_3_-14, H-3/H_3_-14′, H-2/H-4, H-2/H_3_-14 and H-3′/H-3 revealed that these protons were co-facial and were assigned as *α*-oriented. The correlation of H-7/H_3_-15 indicated that H-7 was *β*-oriented. The absolute configuration of **16** was established by comparison of the experimental and calculated ECD data. The calculated ECD spectrum of **16** (Fig. 5) was in accordance with the experimental data closely, pointing toward an absolute configuration of 2*R*, 3*R*, 4*S*, 7*S*, 10*R*, 1′*S*, 3′*S*, 10′*R* for **16**.

In addition, six known compounds, xylopins A and B (**17** and **18**) [9], xylopsides C and D (**19** and **20**) [6], xylopidimer D (**21**) [3] and vieloplain B (**22**) [10], were characterized by comparison of their spectroscopic data with the reported values.

### Cytotoxicity

In the bioactivity assessment, compounds **1**—**16** were tested for their cytotoxic effects in vitro against the PANC-1 and PC-3 cell lines. As shown in Table 5, compound **8** had a relatively stronger cytotoxic effect on the PANC-1 and PC-3 cell lines at IC_50_ values of 1.06 and 9.1 μM, respectively. Compounds **6**, **7**, **9** and **12** showed a moderate cytotoxic activity against PANC-1 and PC-3 cell lines with IC_50_ values ranged from 5.5—20.5 μM. Therefore, compound **8** was further studied for its induction of apoptosis, cell cycle analysis, cell invasion and autophagy according to its potent cytotoxic effect against PANC-1 cells.

**Table 5:** Cytotoxic effects of compounds **1**—**16** with IC_50_ values (μM)

Compounds	Cell lines		Compounds	Cell lines	
	PANC-1	PC-3		PANC-1	PC-3
**1**	> 30	> 30	**10**	> 30	> 30
**2**	> 30	> 30	**11**	> 30	> 30
**3**	> 30	> 30	**12**	20.5	18.8
**4**	> 30	> 30	**13**	> 30	> 30
**5**	> 30	> 30	**14**	> 30	> 30
**6**	11.9	6.7	**15**	> 30	> 30
**7**	5.5	7.3	**16**	> 30	> 30
**8**	1.06	9.1	Taxol	0.64	0.96
**9**	8.1	13.4			

### Effect of compound 8 on apoptosis and cell cycle of PANC-1 cells

**Fig. 6:**
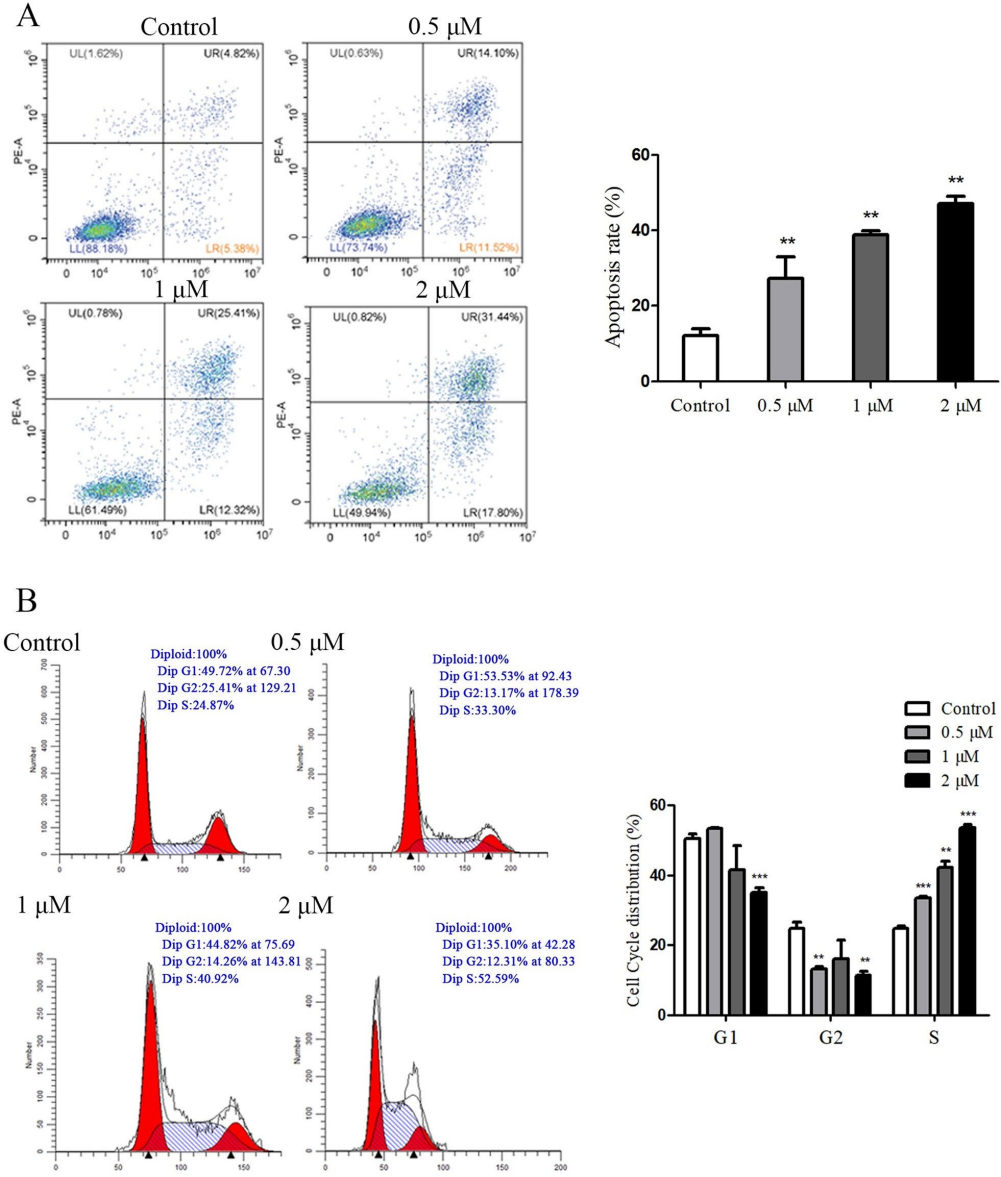
**A** Effect of compound **8** on apoptosis of PANC-1 cells. **B** Effect of compound **8** on the cell cycle distribution of PANC-1 cells

To determine if the decreased cell viability was linked to apoptosis, the Annexin fluorescein isothiocyanate (V-FITC)/propidium iodide (PI) double staining was performed to detect the apoptosis rate. We found that the apoptosis rate of PANC-1 cells increased with the concentration of compound **8** (Fig. 6A), and the apoptosis rate was 49.24% at a concentration of 2 μM.

To examine whether compound **8** inhibited cell growth by arresting the cell cycle in PANC-1 cells, the cell distribution at different phases of the cell cycle was analyzed by flow cytometry, in which PANC-1 cells were incubated in the presence of different concentrations of compound** 8** for 48 h. As shown in Fig. 6B, the percentage of cells in S phase increased corresponding to the increase of the concentration of compound** 8**. Compared to the control group, the compound** 8**-treated group displayed more S distribution and less G1 and G2 distributions, indicating that compound **8** could change the cell cycle by arresting the progression of the S phase.

### Effect of compound 8 on invasion and autophagy of PANC-1 cells

**Fig. 7:**
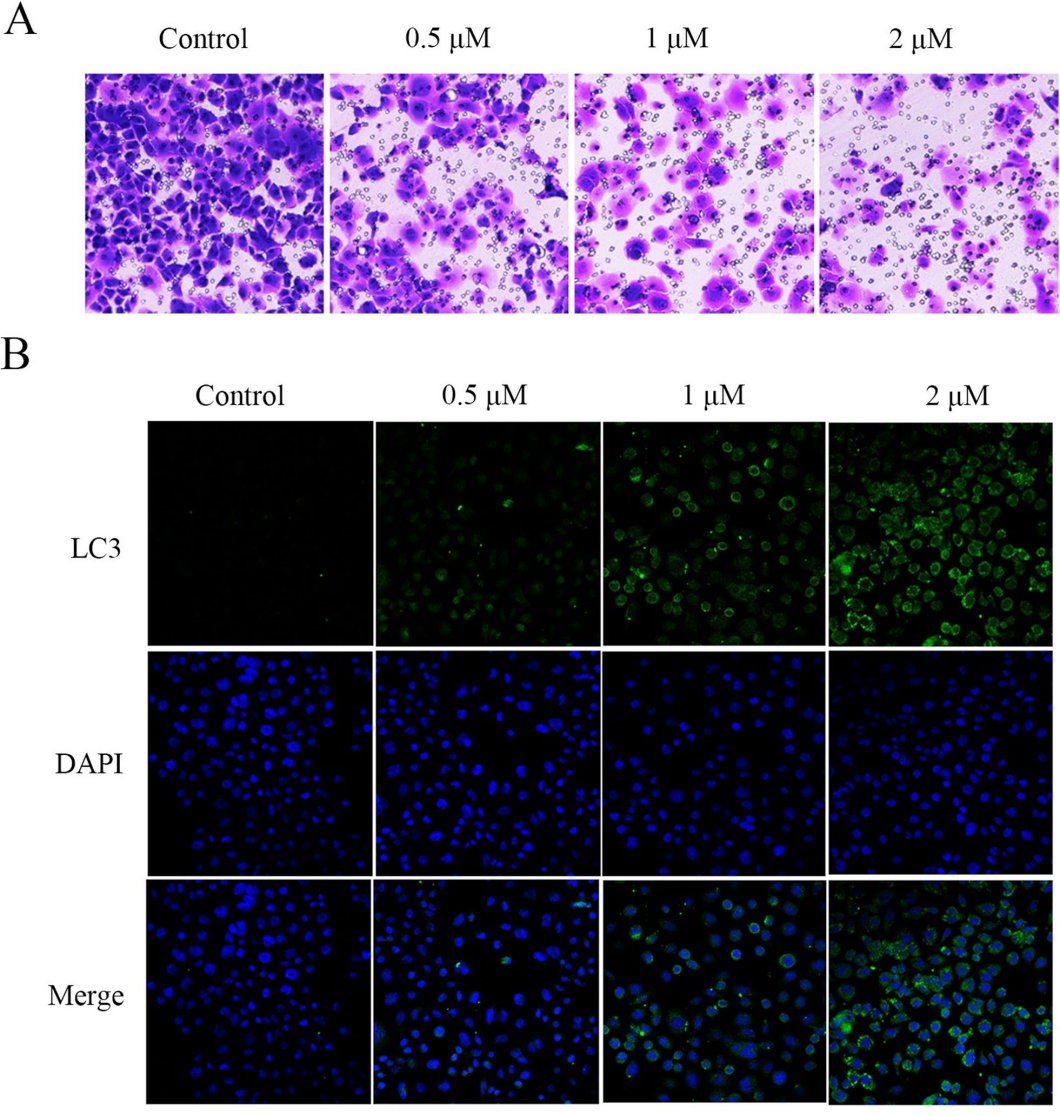
**A** Transwell assay of PANC-1 cells incubated with compound **8** (0.5, 1 and 2 μM) for 48 h. **B** Representative images of LC3 and DAPI immunofluorescence staining in the control group and compound **8**-treated group

We performed cell invasion by transwell test. Transwell assay (Fig. 7A) showed that PANC-1 cells had a strong invasive ability in the control group but compound **8** significantly inhibited PANC-1 cell invasion. Immunofluorescence staining showed that LC3 protein expression was increased in the compound **8**-treated group compared with the control group (Fig. 7B).

## Experimental section

### General experimental procedures

Optical rotation data were acquired on a WZZ-3 polarimeter (Shanghai, China). NMR data were obtained using a Bruker AV-400 spectrometer (Fallanden, Switzerland). HR-ESI–MS analyses were measured on a Bruker micOTOF II and SolariX 7.0 spectrometer (Bruker, Karlsruhe, Germany). High performance liquid chromatography (HPLC) analysis was performed on a SHIMADZU chromatography equipped with LC-20AD pumps, SPD-20A detector and a 250 mm × 10 mm YMC-Pack ODS-A column (YMC, Kyoto, Japan) and a 250 mm × 4.6 mm SHIMADZU-GL column (Shimadzu, Japan).

### Plant material

The branches and leaves of *X. vielana* were collected from Dongxing city, Guangxi province, P. R. China, in September 2018. The identification of the plant material was authenticated by Prof. Chen Min, Southwest University, China. A voucher specimen (No. KMZF2018M001A) has been deposited in School of Pharmacy, Southwest University.

### Extraction and isolation

The sun-dried (5 kg) plant branches and leaves were crushed into small pieces. The plant material was soaked with 95% EtOH (30 L) for 24 h and then extracted three times at 60 °C under reflux. The combined EtOH extracts were concentrated under vacuum to yield a crude residue (163.2 g), which was suspended in water, and subsequently partitioned with petroleum ether, dichloromethane and ethyl acetate. The dichloromethane portion (55.1 g) was subjected to silica gel column chromatography eluting with a step-wise gradient of ethyl acetate (PE)/petroleum ether (EA) (*v/v*, 100:1, 80:1, 60:1, 40:1, 20:1, 10:1, 5:1, 2:1, 1:1) to obtain thirteen fractions (A–M) (monitored by thin layer chromatography).

The Fr. E (3.089 g) was chromatographed on ODS column with a step gradient mixture of MeOH/H_2_O to obtain fractions E1-E12. The Fr. E4 (204 mg) was separated by semi-preparative HPLC (MeOH/H_2_O, 80:20) to yield compounds **2** (*t*_*R*_ 33 min, 43 mg), **12** (*t*_*R*_ 29 min, 23 mg) and **19** (*t*_*R*_ 25 min, 8 mg). Compounds **3** (*t*_*R*_ 34 min, 33 mg), **13** (*t*_*R*_ 38 min, 19 mg) and **21** (*t*_*R*_ 43 min, 5 mg) were obtained from Fr. E6 (287 mg) by semi-preparative HPLC (MeOH/H_2_O, 85:15). The Fr. E9 (136 mg) was separated by semi-preparative HPLC (MeOH/H_2_O, 88:12) to yield compounds **22** (*t*_*R*_ 43 min, 4.5 mg). The Fr. E8 (1.598 g) was subjected to silica gel column using a gradient of PE/EA (*v/v*, 100: 1, 50: 1, 20: 1, 10: 1, 5: 1, 2: 1, 1: 1, 1: 2) to yield seven subfractions E8a-E8g. The Fr. E8c (141 mg) was separated by semi-preparative HPLC (MeOH/H_2_O, 80:20) to yield compounds **1** (*t*_*R*_ 35 min, 56 mg), **6** (*t*_*R*_ 26 min, 8 mg) and **18** (*t*_*R*_ 43 min, 6 mg). The Fr. E8d (390 mg) was separated by semi-preparative HPLC (MeOH/H_2_O, 85:15) to yield compounds **11** (*t*_*R*_ 35 min, 12 mg), **14** (*t*_*R*_ 38 min, 13 mg) and **20** (*t*_*R*_ 29 min, 9 mg).

The Fr. H (1.705 g) was chromatographed on ODS column with a step gradient mixture of MeOH–H_2_O to obtain fractions H1-H7. The Fr. H3 (177 mg) was separated by semi-preparative HPLC (MeOH/H_2_O, 78:22) to yield compounds **5** (*t*_*R*_ 37.5 min, 35 mg), **7** (*t*_*R*_ 31 min, 8 mg), **9** (*t*_*R*_ 43 min, 7 mg) and **17** (*t*_*R*_ 28 min, 11 mg). Compounds **4** (*t*_*R*_ 24 min, 29 mg), **8** (*t*_*R*_ 40.5 min, 21 mg) and **16** (*t*_*R*_ 36 min, 6 mg) were obtained from Fr. H6 (459 mg) by semi-preparative HPLC (MeOH/H_2_O, 90:10). The Fr. H7 (159 mg) was separated by semi-preparative HPLC (MeOH/H_2_O, 90:10) to yield compounds **10** (*t*_*R*_ 42 min, 26 mg) and **15** (*t*_*R*_ 35 min, 11 mg).

### Spectroscopic data of compounds

Xylanin A (**1**): colorless needle crystals; ${[\alpha]^{20}_{D}}$ − 69.9 (c 0.3 CH_2_Cl_2_); mp 190–191 °C; UV (MeOH) λ_max_ (log ε) 221 (0.46) nm; for ^1^H NMR and ^13^C NMR spectroscopic data, see Table 1; HR-ESI–MS (positive): *m/z* 489.2977 [M + Na]^+^ (calcd for C_30_H_42_O_4_Na, 489.2975).

Xylanin B (**2**): colorless crystals; ${[\alpha]^{20}_{D}}$ − 56.9 (c 0.3 CH_2_Cl_2_); mp 178–179 °C; UV (MeOH) λ_max_ (log ε) 232 (0.29) nm; for ^1^H NMR and ^13^C NMR spectroscopic data, see Table 1; HR-ESI–MS (positive): *m/z* 503.3130 [M + Na]^+^ (calcd for C_31_H_44_O_4_Na, 503.3132).

Xylanin C (**3**): yellow oil; ${[\alpha]^{20}_{D}}$ + 38.1 (c 0.3 CH_2_Cl_2_); UV (MeOH) λ_max_ (log ε) 243 (1.23) nm; ECD (1.2 × 10^−5^ M, MeOH) λ (Δ ε) 244 (+ 12.86), 282 (− 5.13); for ^1^H NMR and ^13^C NMR spectroscopic data, see Table 1; HR-ESI–MS (positive): *m/z* 433.3101 [M + H]^+^ (calcd for C_30_H_41_O_2_, 433.3101).

Xylanin D (**4**): colorless oil; ${[\alpha]^{20}_{D}}$ − 96.0 (c 0.3 CH_2_Cl_2_); UV (MeOH) λ_max_ (log ε) 231 (0.85) nm; ECD (1.2 × 10^−5^ M, MeOH) λ (Δ ε) 245 (+ 5.76), 298 (− 14.12); for ^1^H NMR and ^13^C NMR spectroscopic data, see Table 1; HR-ESI–MS (positive): *m/z* 437.3417 [M + H]^+^ (calcd for C_30_H_45_O_2_, 437.3414).

Xylanin E (**5**): white powder; ${[\alpha]^{20}_{D}}$–28.1 (c 0.3 CH_2_Cl_2_); UV (MeOH) λ_max_ (log ε) 245 (0.85) nm; ECD (1.2 × 10^−5^ M, MeOH) λ (Δ ε) 245 (+ 6.30), 291 (− 9.26); for ^1^H NMR and ^13^C NMR spectroscopic data, see Table 2; HR-ESI–MS (positive): *m/z* 435.3254 [M + H]^+^ (calcd for C_30_H_43_O_2_, 435.3258).

Xylanin F (**6**): yellow powder; ${[\alpha]^{20}_{D}}$ + 41.9 (c 0.3 CH_2_Cl_2_); UV (MeOH) λ_max_ (log ε) 241 (0.29) nm; ECD (1.2 × 10^−5^ M, MeOH) λ (Δ ε) 221 (− 6.89), 255 (+ 10.53), 295 (− 10.28), 341 (+ 1.73); for ^1^H NMR and ^13^C NMR spectroscopic data, see Table 2; HR-ESI–MS (positive): *m/z* 435.3264 [M + H]^+^ (calcd for C_30_H_43_O_2_, 435.3258).

Xylanin G (**7**): yellow oil; ${[\alpha]^{20}_{D}}$–145.3 (c 0.3 CH_2_Cl_2_); UV (MeOH) λ_max_ (log ε) 216 (0.63) nm; ECD (1.2 × 10^−5^ M, MeOH) λ (Δ ε) 249 (− 50.98), 312 (+ 1.13); for ^1^H NMR and ^13^C NMR spectroscopic data, see Table 2; HR-ESI–MS (positive): *m/z* 501.2675 [M + Na]^+^ (calcd for C_30_H_38_O_5_Na, 501.2670).

Xylanin H (**8**): colorless oil; ${[\alpha]^{20}_{D}}$ –51.3 (c 0.3 CH_2_Cl_2_); UV (MeOH) λ_max_ (log ε) 249 (1.47) nm; ECD (1.2 × 10^−5^ M, MeOH) λ (Δ ε) 243 (+ 57.18), 312 (− 34.33); for ^1^H NMR and ^13^C NMR spectroscopic data, see Table 2; HR-ESI–MS (positive): *m/z* 477.2625 [M + H]^+^ (calcd for C_30_H_37_O_5_, 477.2636).

Xylanin I (**9**): white powder; ${[\alpha]^{20}_{D}}$ –91.7 (c 0.3 CH_2_Cl_2_); UV (MeOH) λ_max_ (log ε) 234 (0.62) nm; ECD (1.2 × 10^−5^ M, MeOH) λ (Δ ε) 274 (− 15.13), 326 (− 0.08); for ^1^H NMR and ^13^C NMR spectroscopic data, see Table 3; HR-ESI–MS (positive): *m/z* 433.3103 [M + H]^+^ (calcd for C_30_H_41_O_2_, 433.3101).

Xylanin J (**10**): white powder; ${[\alpha]^{20}_{D}}$ –124.76 (c 0.3 CH_2_Cl_2_); UV (MeOH) λ_max_ (log ε) 241 (1.14) nm; ECD (1.2 × 10^−5^ M, MeOH) λ (Δ ε) 236 (− 53.56), 277 (− 3.90); for ^1^H NMR and ^13^C NMR spectroscopic data, see Table 3; HR-ESI–MS (positive): *m/z* 463.2840 [M + H]^+^ (calcd for C_30_H_39_O_4_, 463.2843).

Xylanin K (**11**): yellow oil; ${[\alpha]^{20}_{D}}$ + 88.4 (c 0.3 CH_2_Cl_2_); UV (MeOH) λ_max_ (log ε) 248 (0.63) nm; ECD (1.2 × 10^−5^ M, MeOH) λ (Δ ε) 232 (− 6.21), 275 (+ 17.35), 311 (− 13.32); for ^1^H NMR and ^13^C NMR spectroscopic data, see Table 3; HR-ESI–MS (positive): *m/z* 455.2916 [M + Na]^+^ (calcd for C_30_H_40_O_2_Na, 455.2921).

Xylanin L (**12**): colorless oil; ${[\alpha]^{20}_{D}}$ + 82.8 (c 0.3 CH_2_Cl_2_); UV (MeOH) λ_max_ (log ε) 296 (2.51) nm; ECD (1.2 × 10^−5^ M, MeOH) λ (Δ ε) 229 (− 16.30), 258 (+ 5.78), 290 (− 14.7), 328 (+ 21.88); for ^1^H NMR and ^13^C NMR spectroscopic data, see Table 3; HR-ESI–MS (positive): *m/z* 543.2720 [M + Na]^+^ (calcd for C_32_H_40_O_6_Na, 543.2717).

Xylanin M (**13**): white amorphous powder; ${[\alpha]^{20}_{D}}$ –84.1 (c 0.3 CH_2_Cl_2_); UV (MeOH) λ_max_ (log ε) 237 (0.84) nm; ECD (1.2 × 10^−5^ M, MeOH) λ (Δ ε) 227 (+ 2.81), 299 (− 15.83); for ^1^H NMR and ^13^C NMR spectroscopic data, see Table 4; HR-ESI–MS (positive): *m/z* 437.3404 [M + H]^+^ (calcd for C_30_H_45_O_2_, 437.3414).

Xylanin N (**14**): white amorphous powder; ${[\alpha]^{20}_{D}}$ –51.6 (c 0.3 CH_2_Cl_2_); UV (MeOH) λ_max_ (log ε) 236 (0.19) nm; ECD (1.2 × 10^−5^ M, MeOH) λ (Δ ε) 231 (+ 3.91), 300 (− 7.61); for ^1^H NMR and ^13^C NMR spectroscopic data, see Table 4; HR-ESI–MS (positive): *m/z* 457.3077 [M + Na]^+^ (calcd for C_30_H_42_O_2_Na, 457.3077).

Xylanin O (**15**): white powder; ${[\alpha]^{20}_{D}}$ –69.6 (c 0.3 CH_2_Cl_2_); UV (MeOH) λ_max_ (log ε) 237 (0.26) nm; ECD (1.2 × 10^−5^ M, MeOH) λ (Δ ε) 216 (− 3.35), 248 (+ 1.38), 298 (− 8.21); for ^1^H NMR and ^13^C NMR spectroscopic data, see Table 4; HR-ESI–MS (positive): *m/z* 459.3225 [M + Na]^+^ (calcd for C_30_H_44_O_2_Na, 459.3234).

Xylanin P (**16**): yellow powder; ${[\alpha]^{20}_{D}}$ –87.9 (c 0.3 CH_2_Cl_2_); UV (MeOH) λ_max_ (log ε) 228 (0.61) nm; ECD (1.2 × 10^−5^ M, MeOH) λ (Δ ε) 211 (+ 2.66), 226 (+ 6.08), 278 (− 17.73); for ^1^H NMR and ^13^C NMR spectroscopic data, see Table 4; HR-ESI–MS (positive): *m/z* 457.3076 [M + Na]^+^ (calcd for C_30_H_42_O_2_Na, 457.3077).

### X-ray crystallographic analysis of compounds 1 and 2

A suitable crystal was selected and then performed on a SuperNova, Dual, Cu at home/near, EosS2 diffractometer. The crystal was kept at 294.5 K during data collection. The structure was then solved utilizing the ShelXS structure solution program in conjunction with direct methods, and further refined using the ShelXL refinement package through least squares minimization.

Crystal data for compound **1**: C_30_H_42_O_4_, M = 466.64, a = 16.6944(3) Å, b = 17.4380(3) Å, c = 19.0842(2) Å, *α* = 90^◦^, *β* = 90^◦^, *γ* = 90^◦^, V = 5555.75(14) Å3, T = 295 K, space group P21, Z = 4, μ (Cu K*α*) = 0.567 mm^−1^, 53,317 reflections collected, 10,784 independent reflections (Rint = 0.0412). The goodness of fit on F^2^ was 1.035. Flack parameter = −0.15(12). The final R_1_ values were 0.0463 (I > 2σ(I)). The final wR (F^2^) values were 0.1220 (I > 2σ(I)). The final R_1_ values were 0.0582 (all data). The final wR (F^2^) values were 0.1297 (all data). CCDC deposition number was 2267636.

Crystal data for compound **2**: C_31_H_44_O_4_, M = 480.66, a = 10.06326(10) Å, b = 16.21942(18) Å, c = 17.23224(18) Å, *α* = 90^◦^, *β* = 90^◦^, *γ* = 90^◦^, V = 2812.65(5) Å^3^, T = 180 K, space group P21, Z = 4, μ (Cu K*α*) = 0.573 mm^−1^, 52,884 reflections collected, 5507 independent reflections (Rint = 0.0897). The goodness of fit on F^2^ was 1.026. Flack parameter = −0.04(10). The final R_1_ values were 0.0392 (I > 2σ(I)). The final wR (F^2^) values were 0.1051 (I > 2σ(I)). The final R_1_ values were 0.0401 (all data). The final wR (F^2^) values were 0.1063 (all data). CCDC deposition number was 2267637.

### ECD calculations

Firstly, we needed to search random conformation by SYBYL X 2.0 program using MMFF94s molecular force field, with an energy cutoff of 10 kcal mol^−1^ to the global minima [14]. Subsequently, the geometric structure and frequency analysis of the molecular conformation were optimized using density functional theory (DFT) under the B3LYP/6–31 + G(d,p) method. Next, the electron circular dichroism (ECD) of compounds was calculated using time dependent density functional theory (TDDFT) at the same group level. Finally, the ECD spectra calculated by quantum chemistry were compared with those measured by experiment, and the absolute configurations of the compounds were determined [15–17].

### Cytotoxicity assay

The details of the cytotoxicity assay on two human tumor cell lines (PANC-1 and PC-3) were described below. Briefly, 1 × 10^4^ cells/mL were seeded in 96 well plates and incubated for 24 h. Then, cells were treated with different concentrations of the tested compounds in the growth medium for 48 h. After that, 10 μL of cell counting kit-8 (CCK-8, Targetmol) was added to each well and incubation was conducted 90 min at 37 ℃. The absorbance was measured using Synergy H4 Hybrid Microplate Reader (BioTek) at 450 nm. The absorbance of cells treated with 0.1% DMSO was considered as 100% and the IC_50_ represented the concentration that inhibited cell proliferation by 50%. Taxol was used as the positive control [18–20].

### Cell apoptosis

Apoptosis was determined by flow cytometric analysis of Annexin V-FITC/PI staining [21]. Briefly, the PANC-1 cells were cultured for 24 h in 6 well plates. When the cells grew to a density of 2 × 10^5^ cells/mL, compound **8** (0.5, 1 and 2 μM) was added for 48 h. The cells were washed 1–2 times with phosphate buffered saline (PBS) and then centrifuged at 3400 r/min for 5 min. The cells were suspended with 195 μL Annexin V-FITC binding solution, 5 μL Annexin V-FITC, and 10 μL PI staining solution, and mixed gently. After that, the cells were incubated at room temperature for 20 min under light protection and detected by flow cytometer [22, 23].

### Cell cycle

Different phases of the cell cycle distribution were determined by the PI Flow Cytometry Kit, based on the manufacturer’s protocol. Briefly, after incubated with compound **8** (0.5, 1 and 2 μM) for 48 h, cells were washed twice with PBS, and fixed with ice-cold 70% ethanol. After rinsed with PBS, cells were further treated with RNase and PI at room temperature for 30 min in the dark. Then, the flow cytometer was used to analyze samples [24, 25].

### Transwell assay

Transwell assay was used to evaluate the ability of compound **8** to prevent PANC-1 cell invasion in vitro. Cells were inoculated on 12 well plates with 1 × 105 cells/mL per well. After 48 h treatment with 0.5, 1 and 2 μM compound **8**, the cells were digested and transferred to the upper compartment of the transwell cell coated with matrix glue. The medium containing 20% fetal bovine serum was added to the lower chamber, and it was discarded after 24 h culture. Afterwards, the invaded cells were added with 4% paraformaldehyde for fixation for 15 min, 0.1% crystal violet for staining for 30 min at room temperature and washed with PBS buffer for 3 times. The cells were observed under the optical microscope [26, 27].

### Immunofluorescence analysis

Compound **8**-treated PANC-1 cells were washed twice with cold PBS, fixed with 4% cold paraformaldehyde for 15 min, and permeated with 0.1% Triton X-100 for 15 min. Next, the cells were blocked with 5% BSA for 1 h and incubated with primary antibody overnight at 4 ℃. Alexa-conjugated secondary antibodies were applied and incubated at room temperature for 1 h. Cell nuclei were stained with 4,6-diamino-2-phenyl indole (DAPI, Cell Signaling Technology, MA, USA) for 10 min. Finally, the cell images were analyzed by ImageXpress^®^ Micro Confocal [11, 28].

### Statistical analysis

All the experiments were carried out in triplicate and the data were carefully analyzed with the statistical software GraphPad Prism. The data were statistically analyzed with one-way ANOVA followed by the Dunnett's posthoc test, and the level of statistical significance was considered to be *P* < 0.05. The data were expressed as mean ± SD.

## Conclusion

Sesquiterpenoids and their dimers were reported to exhibit diverse pharmacological activities, including antitumor and anti-inflammatory effects, and they were a group of effective and low-toxicity natural small molecules [29–31]. Guaiane-type dimers were primarily isolated from plants of the *Inula* and *Artemisia* genera [32]. Lavandiolide H, isolated from *Artemisia atrovirens*, exerted anti-hepatoma effects via inhibition of cell migration and invasion, and induction of G2/M phase cell cycle arrest and apoptosis. The molecular mechanism involved the downregulation of BCL-2 and PARP-1 expression, and PARP-1 activation, leading to the accumulation of cleaved-PARP-1 [33]. Pharmacological studies revealed that lineariifolianoid A, isolated from *Inula lineariifolia*, inhibited breast cancer proliferation by targeting the p53-independent NFAT1-MDM2 pathway. It also displayed modulatory effects on the expression of key proteins involved in cell cycle progression, apoptosis, and DNA damage [34, 35]. Inulanolide A, isolated from *Inula japonica*, was also identified as a dual inhibitor of the NFAT1-MDM2 pathway. It demonstrated potent anti-proliferative and anti-metastatic activities against breast and prostate cancer in both in vitro and in vivo models [36]. Due to their potential anti-tumor activity and complex structures, guaiane-type dimers attracted considerable research interest.

In this study, twenty-two dimeric guaianes including sixteen new compounds were isolated from branches and leaves of *X. vielana* and identified by NMR spectroscopic data, HR-ESI–MS data, X-ray diffraction analyses, and ECD spectra. Sixteen new compounds were tested for their cytotoxic activities and five of them showed good cytotoxic activities. Compound **8** had a relatively stronger cytotoxic effect against PANC-1 and PC-3 cell lines with IC_50_ values of 1.06 and 9.1 μM, respectively.

In apoptosis assay, compound **8** could induce PANC-1 cell apoptosis. Next, we found that the cell cycle of PANC-1 cells was arrested at S phase by the treatment of compound **8**. By the invasion test, compound **8** was found to restrain the invasion of PANC-1 cells. In autophagy assay, we observed increased LC3 by immunofluorescence in the compound **8**-treated group. In conclusion, compound **8** had a relatively good cytotoxic effect on PANC-1 cells and could be considered as a potential candidate compound.

The structure–activity relationships (SAR) were analyzed based on the cytotoxicity data of the compounds. The activity of compound **7**, compared to the inactive compound **1**, underscored the necessity of the double bond in unit B for cytotoxic activity. The contrast between the active compound **9** and the inactive compound **3** established the absolute configuration of unit B as a critical determinant of activity. Furthermore, the activity of compound **12** relative to compound **10** demonstrated the central role of the C-2' acetoxy group in suppressing the proliferation of PANC-1 and PC-3 cell lines. The analysis of the structure–activity relationships provides a foundation for the enhancement of the activity of such dimers through structural modification. In summary, the guaiane-type dimers represent a class of promising antitumor lead compounds.

## Supplementary Information

Supplementary material 1.

## Data Availability

Data will be made available on request.
