# Supplementary material for: Xylanins A–P, sixteen new guaiane-type dimers from the branches and leaves of Xylopia vielana with anti-proliferative activity against PANC-1 cell line
Source: Nat Prod Bioprospect. 2026 Jan 11;16(1):19. doi: 10.1007/s13659-025-00574-z (PMC12790548; doi:10.1007/s13659-025-00574-z)
Supplement: Supplementary file 1 — Supplementary material 1. [file 13659_2025_574_MOESM1_ESM.docx]

**Xylanins A–P, sixteen new guaiane-type dimers from the branches and leaves of *Xylopia vielana*** **with anti-proliferative activity against PANC-1 cell line**

Xianglian Jiang, Ting Zhang, Fancheng Meng, Min Chen, Guowei Wang*

College of Pharmaceutical Sciences, Southwest University, Chongqing, China

* Corresponding author at: College of Pharmaceutical Sciences, Southwest University, Chongqing, China.

E-mail address: wangguowei@swu.edu.cn

Table of content

Figure S1. ^1^H NMR spectrum of **1**. 5

Figure S2. ^13^C NMR spectrum of **1**. 5

Figure S3. DEPT spectrum of **1**. 6

Figure S4. ^1^H-^1^H COSY spectrum of **1**. 6

Figure S5. HSQC spectrum of **1**. 7

Figure S6. HMBC spectrum of **1**. 7

Figure S7. NOESY spectrum of **1**. 8

Figure S8. HR-ESI-MS spectrum of **1**. 8

Figure S9. ^1^H NMR spectrum of **2**. 9

Figure S10. ^13^C NMR spectrum of **2**. 9

Figure S11. DEPT spectrum of **2**. 10

Figure S12. ^1^H-^1^H COSY spectrum of **2**. 10

Figure S13. HSQC spectrum of **2**. 11

Figure S14. HMBC spectrum of **2**. 11

Figure S15. NOESY spectrum of **2**. 12

Figure S16. HR-ESI-MS spectrum of **2**. 12

Figure S17. ^1^H NMR spectrum of **3**. 13

Figure S18. ^13^C NMR spectrum of **3**. 13

Figure S19. DEPT spectrum of **3**. 14

Figure S20. ^1^H-^1^H COSY spectrum of **3**. 14

Figure S21. HSQC spectrum of **3**. 15

Figure S22. HMBC spectrum of **3**. 15

Figure S23. NOESY spectrum of **3**. 16

Figure S24. HR-ESI-MS spectrum of **3**. 16

Figure S25. ^1^H NMR spectrum of **4**. 17

Figure S26. ^13^C NMR spectrum of **4**. 17

Figure S27. DEPT spectrum of **4**. 18

Figure S28. ^1^H-^1^H COSY spectrum of **4**. 18

Figure S29. HSQC spectrum of **4**. 19

Figure S30. HMBC spectrum of **4**. 19

Figure S31. NOESY spectrum of **4**. 20

Figure S32. HR-ESI-MS spectrum of **4**. 20

Figure S33. ^1^H NMR spectrum of **5**. 21

Figure S34. ^13^C NMR spectrum of **5**. 21

Figure S35. DEPT spectrum of **5**. 22

Figure S36. ^1^H-^1^H COSY spectrum of **5**. 22

Figure S37. HSQC spectrum of **5**. 23

Figure S38. HMBC spectrum of **5**. 23

Figure S39. NOESY spectrum of **5**. 24

Figure S40. HR-ESI-MS spectrum of **5**. 24

Figure S41. ^1^H NMR spectrum of **6**. 25

Figure S42. ^13^C NMR spectrum of **6**. 25

Figure S43. DEPT spectrum of **6**. 26

Figure S44. ^1^H-^1^H COSY spectrum of **6**. 26

Figure S45. HSQC spectrum of **6**. 27

Figure S46. HMBC spectrum of **6**. 27

Figure S47. NOESY spectrum of **6**. 28

Figure S48. HR-ESI-MS spectrum of **6**. 28

Figure S49. ^1^H NMR spectrum of **7**. 29

Figure S50. ^13^C NMR spectrum of **7**. 29

Figure S51. DEPT spectrum of **7**. 30

Figure S52. ^1^H-^1^H COSY spectrum of **7**. 30

Figure S53. HSQC spectrum of **7**. 31

Figure S54. HMBC spectrum of **7**. 31

Figure S55. NOESY spectrum of **7**. 32

Figure S56. HR-ESI-MS spectrum of **7**. 32

Figure S57. ^1^H NMR spectrum of **8**. 33

Figure S58. ^13^C NMR spectrum of **8**. 33

Figure S59. DEPT spectrum of **8**. 34

Figure S60. ^1^H-^1^H COSY spectrum of **8**. 34

Figure S61. HSQC spectrum of **8**. 35

Figure S62. HMBC spectrum of **8**. 35

Figure S63. NOESY spectrum of **8**. 36

Figure S64. HR-ESI-MS spectrum of **8**. 36

Figure S65. ^1^H NMR spectrum of **9**. 37

Figure S66. ^13^C NMR spectrum of **9**. 37

Figure S67. DEPT spectrum of **9**. 38

Figure S68. ^1^H-^1^H COSY spectrum of **9**. 38

Figure S69. HSQC spectrum of **9**. 39

Figure S70. HMBC spectrum of **9**. 39

Figure S71. NOESY spectrum of **9**. 40

Figure S72. HR-ESI-MS spectrum of **9**. 40

Figure S73. ^1^H NMR spectrum of **10**. 41

Figure S74. ^13^C NMR spectrum of **10**. 41

Figure S75. DEPT spectrum of **10**. 42

Figure S76. ^1^H-^1^H COSY spectrum of **10**. 42

Figure S77. HSQC spectrum of **10**. 43

Figure S78. HMBC spectrum of **10**. 43

Figure S79. NOESY spectrum of **10**. 44

Figure S80. HR-ESI-MS spectrum of **10**. 44

Figure S81. ^1^H NMR spectrum of **11**. 45

Figure S82. ^13^C NMR spectrum of **11**. 45

Figure S83. DEPT spectrum of **11**. 46

Figure S84. ^1^H-^1^H COSY spectrum of **11**. 46

Figure S85. HSQC spectrum of **11**. 47

Figure S86. HMBC spectrum of **11**. 47

Figure S87. NOESY spectrum of **11**. 48

Figure S88. HR-ESI-MS spectrum of **11**. 48

Figure S89. ^1^H NMR spectrum of **12**. 49

Figure S90. ^13^C NMR spectrum of **12**. 49

Figure S91. DEPT spectrum of **12**. 50

Figure S92. ^1^H-^1^H COSY spectrum of **12**. 50

Figure S93. HSQC spectrum of **12**. 51

Figure S94. HMBC spectrum of **12**. 51

Figure S95. NOESY spectrum of **12**. 52

Figure S96. HR-ESI-MS spectrum of **12**. 52

Figure S97. ^1^H NMR spectrum of **13**. 53

Figure S98. ^13^C NMR spectrum of **13**. 53

Figure S99. DEPT spectrum of **13**. 54

Figure S100. ^1^H-^1^H COSY spectrum of **13**. 54

Figure S101. HSQC spectrum of **13**. 55

Figure S102. HMBC spectrum of **13**. 55

Figure S103. NOESY spectrum of **13**. 56

Figure S104. HR-ESI-MS spectrum of **13**. 56

Figure S105. ^1^H NMR spectrum of **14**. 57

Figure S106. ^13^C NMR spectrum of **14**. 57

Figure S107. DEPT spectrum of **14**. 58

Figure S108. ^1^H-^1^H COSY spectrum of **14**. 58

Figure S109. HSQC spectrum of **14**. 59

Figure S110. HMBC spectrum of **14**. 59

Figure S111. NOESY spectrum of **14**. 60

Figure S112. HR-ESI-MS spectrum of **14**. 60

Figure S113. ^1^H NMR spectrum of **15**. 61

Figure S114. ^13^C NMR spectrum of **15**. 61

Figure S115. DEPT spectrum of **15**. 62

Figure S116. ^1^H-^1^H COSY spectrum of **15**. 62

Figure S117. HSQC spectrum of **15**. 63

Figure S118. HMBC spectrum of **15**. 63

Figure S119. NOESY spectrum of **15**. 64

Figure S120. HR-ESI-MS spectrum of **15**. 64

Figure S121. ^1^H NMR spectrum of **16**. 65

Figure S122. ^13^C NMR spectrum of **16**. 65

Figure S123. DEPT spectrum of **16**. 66

Figure S124. ^1^H-^1^H COSY spectrum of **16**. 66

Figure S125. HSQC spectrum of **16**. 67

Figure S126. HMBC spectrum of **16**. 67

Figure S127. NOESY spectrum of **16**. 68

Figure S128. HR-ESI-MS spectrum of **16**. 68

Figure S1. ^1^H NMR spectrum of **1**.


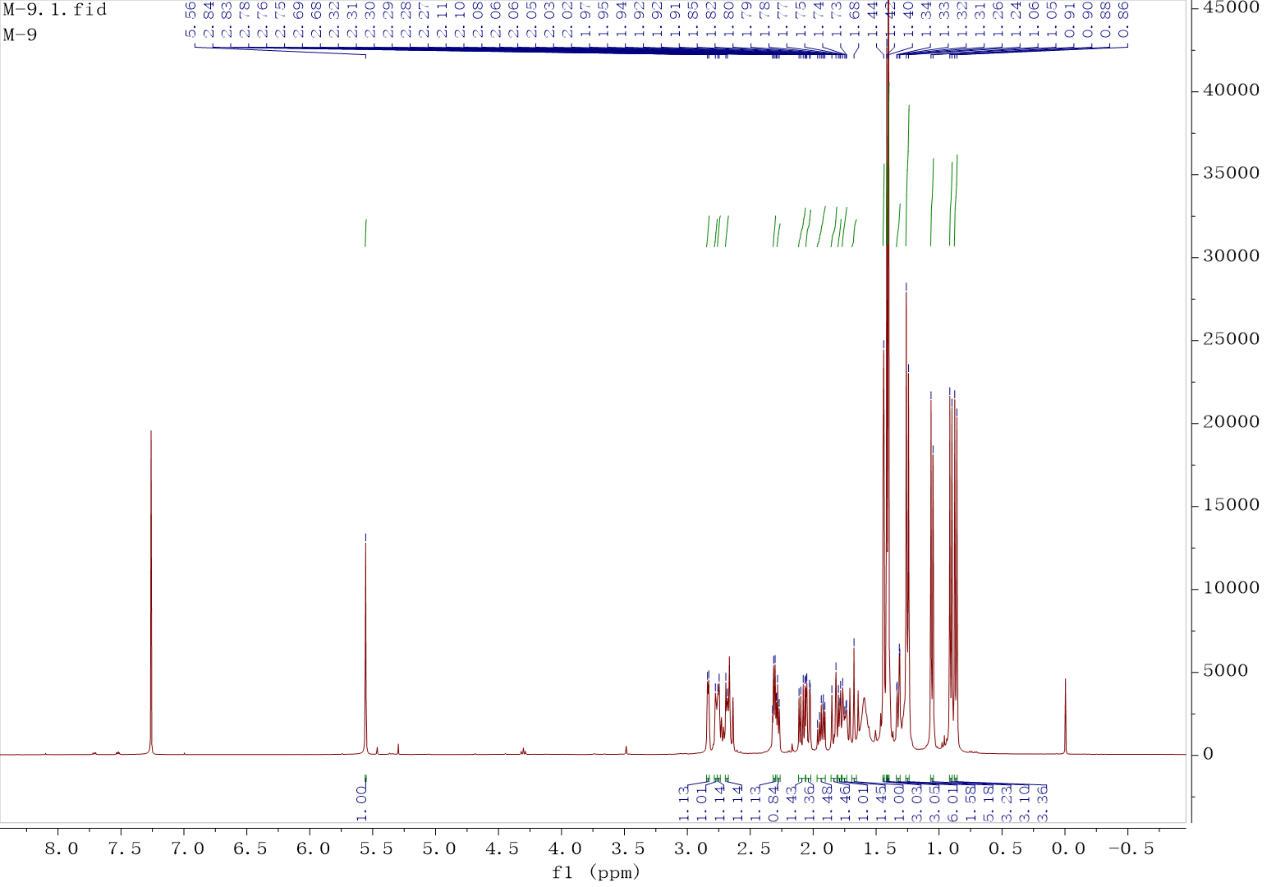


Figure S2. ^13^C NMR spectrum of **1**.


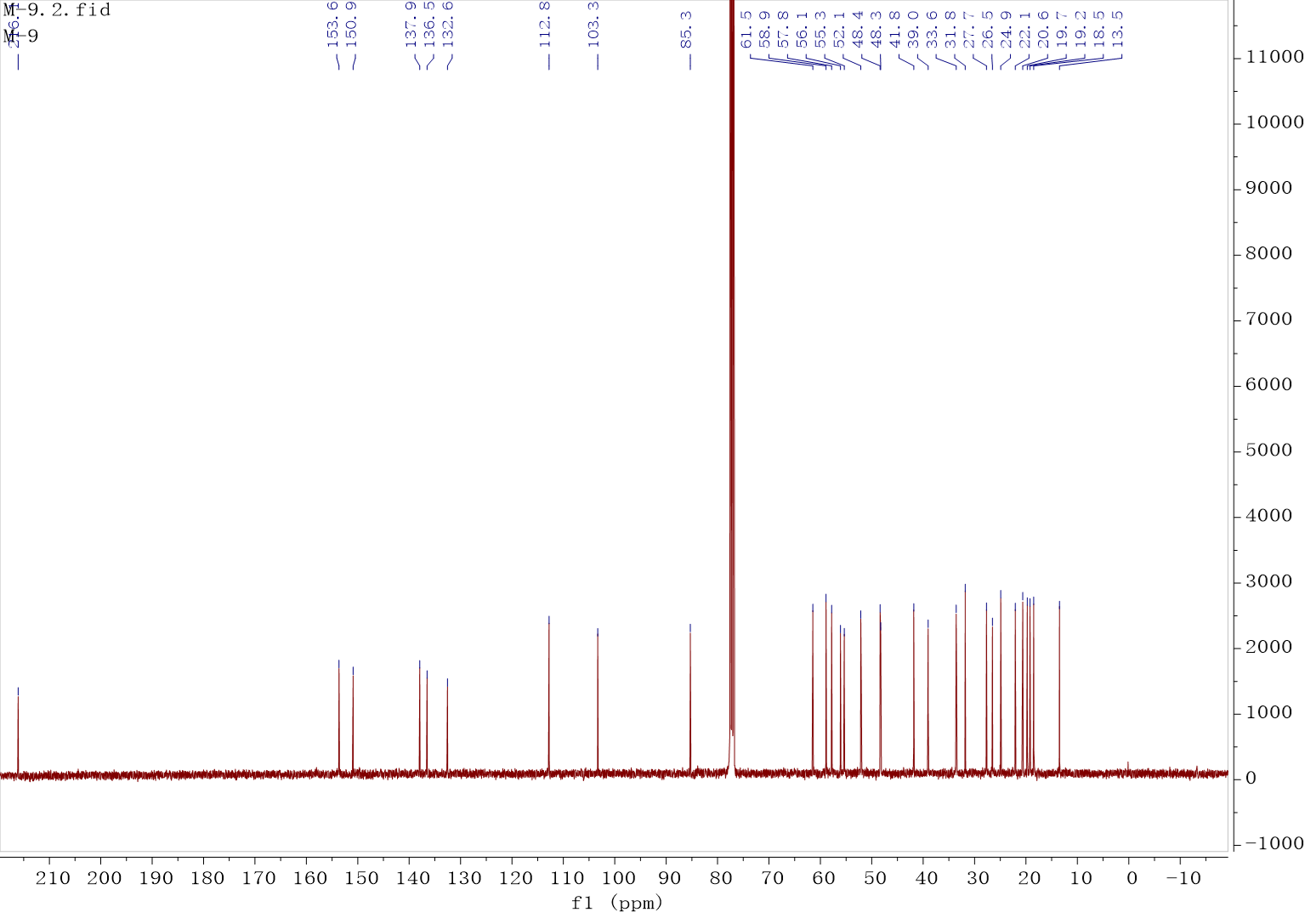


Figure S3. DEPT spectrum of **1**.


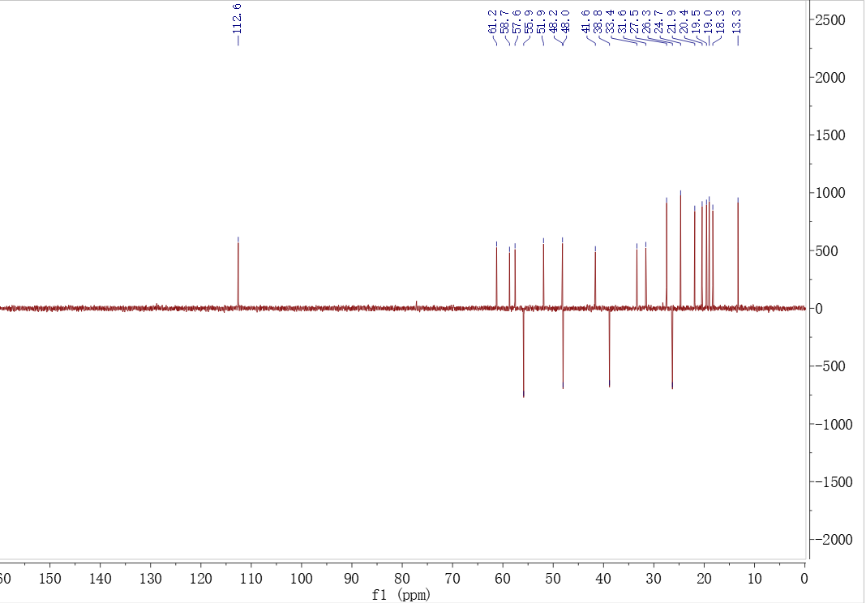


Figure S4. ^1^H-^1^H COSY spectrum of **1**.


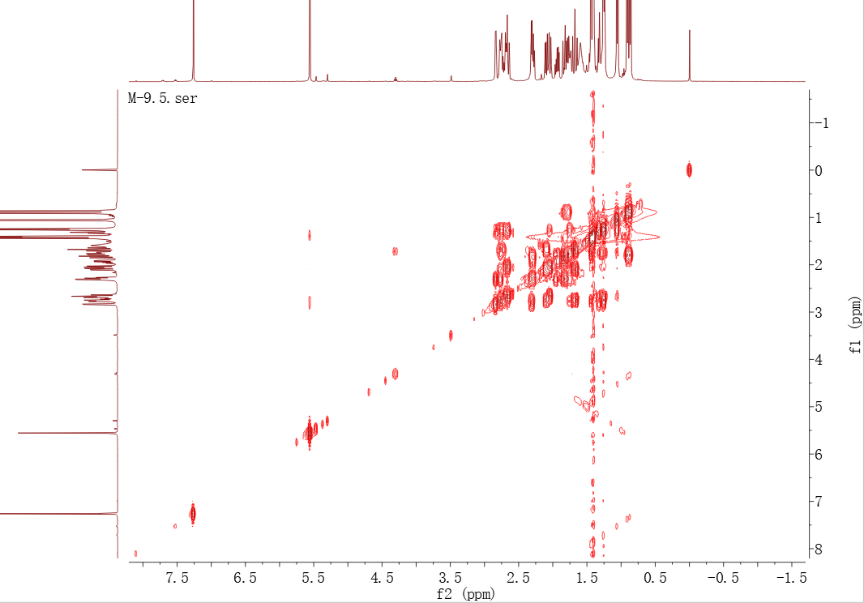


Figure S5. HSQC spectrum of **1**.


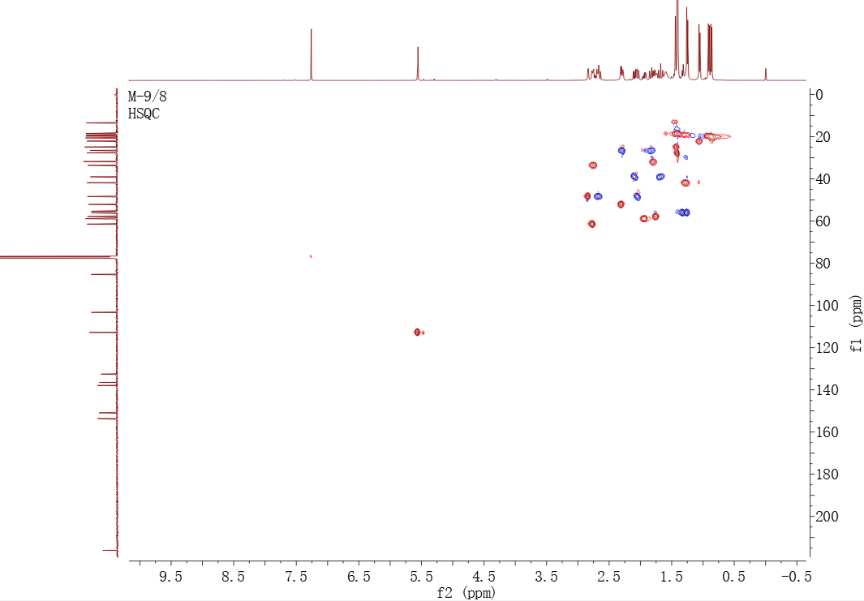


Figure S6. HMBC spectrum of **1**.


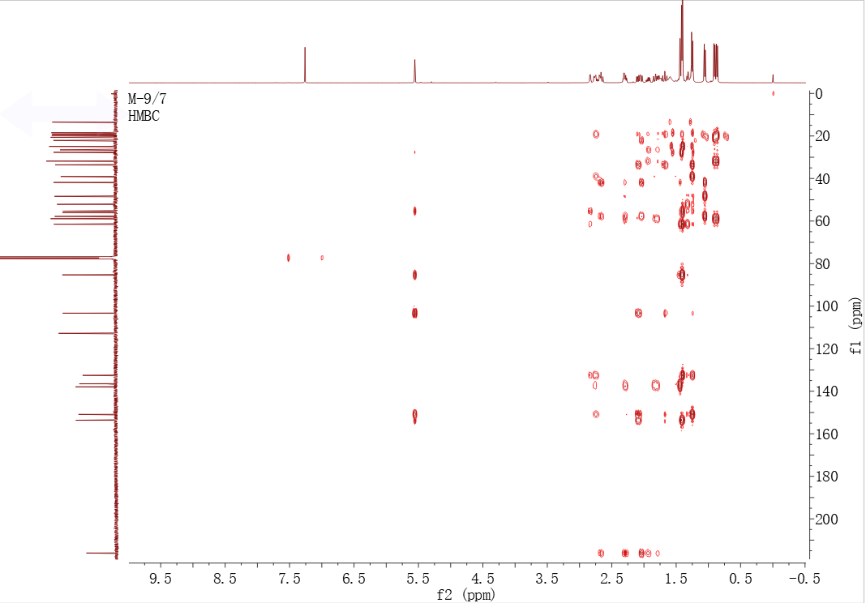


Figure S7. NOESY spectrum of **1**.


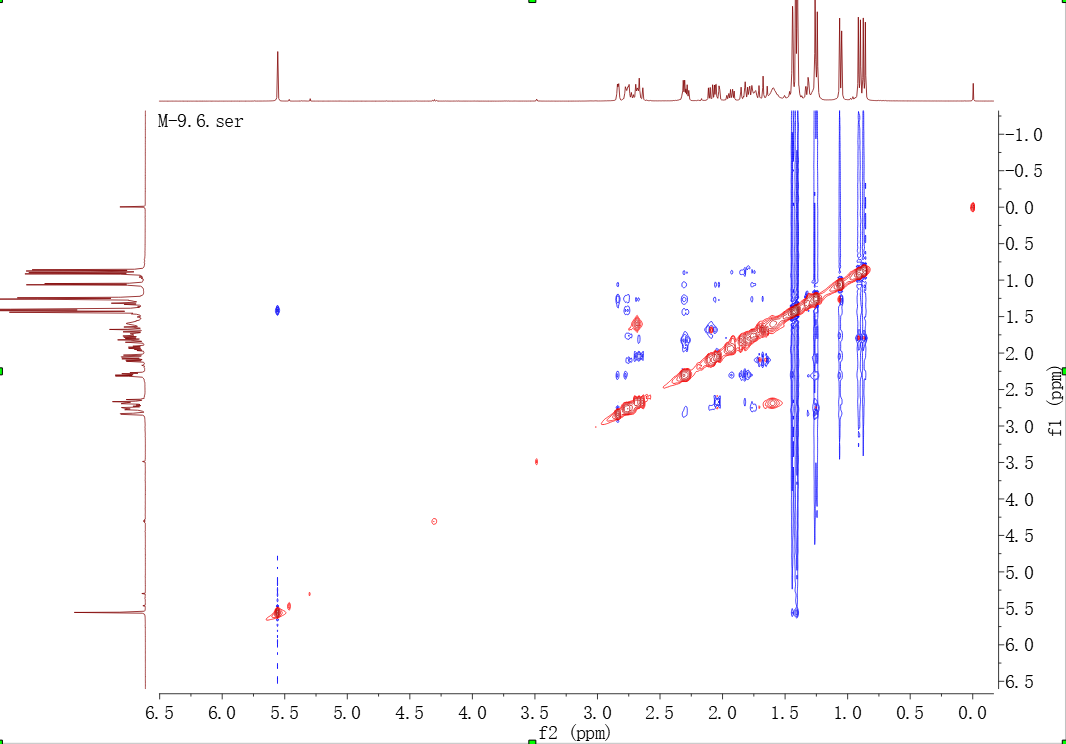


Figure S8. HR-ESI-MS spectrum of **1**.


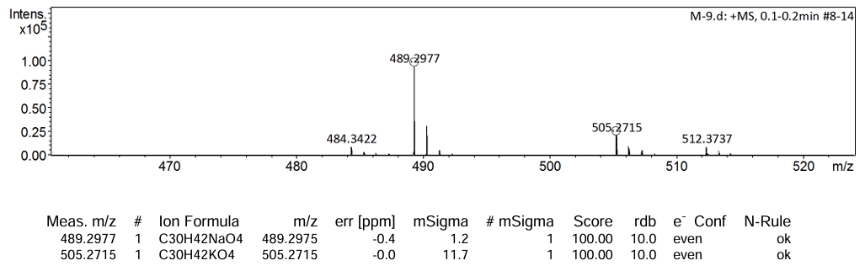


Figure S9. ^1^H NMR spectrum of **2**.


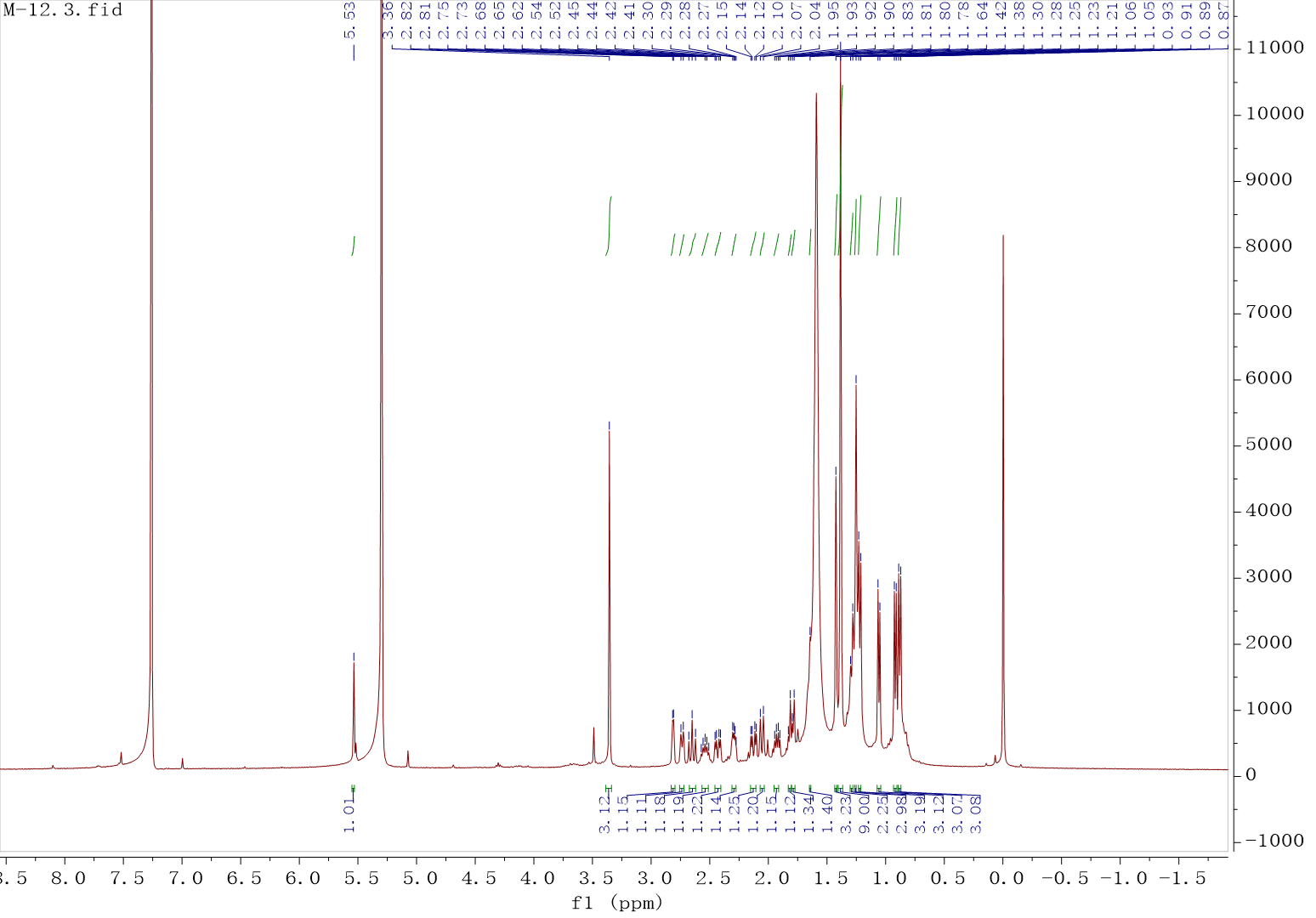


Figure S10. ^13^C NMR spectrum of **2**.


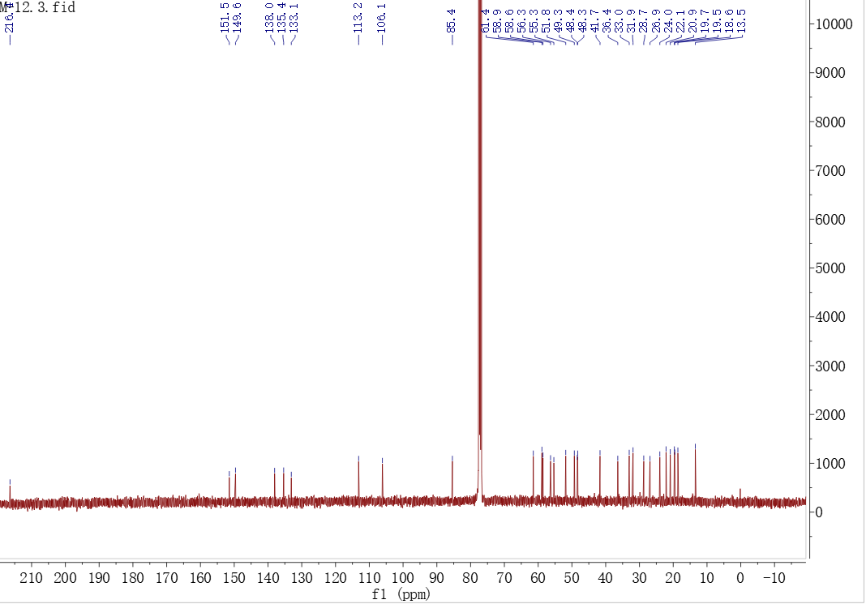


Figure S11. DEPT spectrum of **2**.


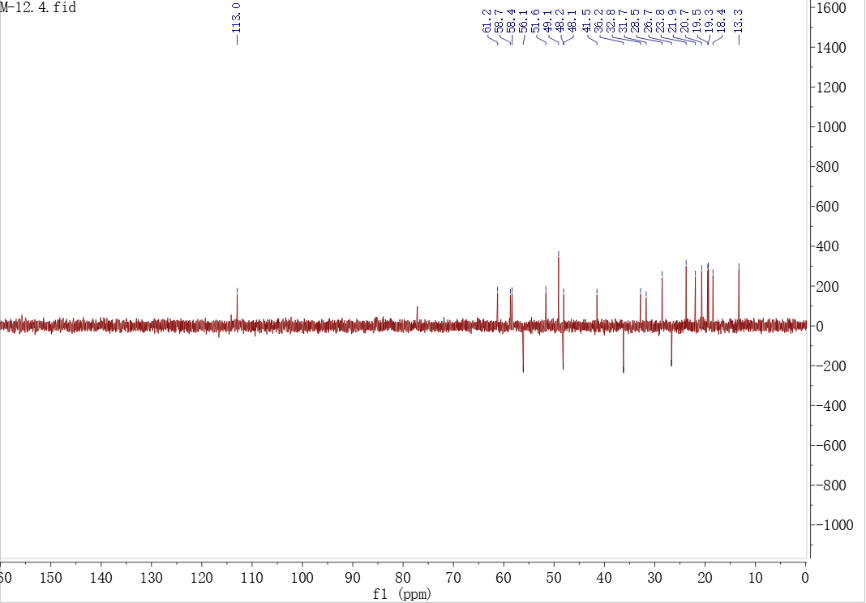


Figure S12. ^1^H-^1^H COSY spectrum of **2**.


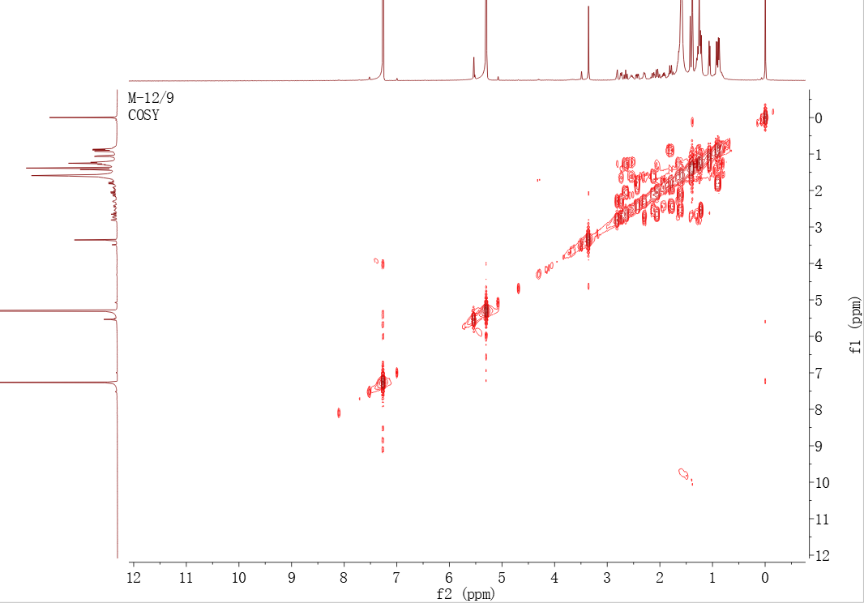


Figure S13. HSQC spectrum of **2**.


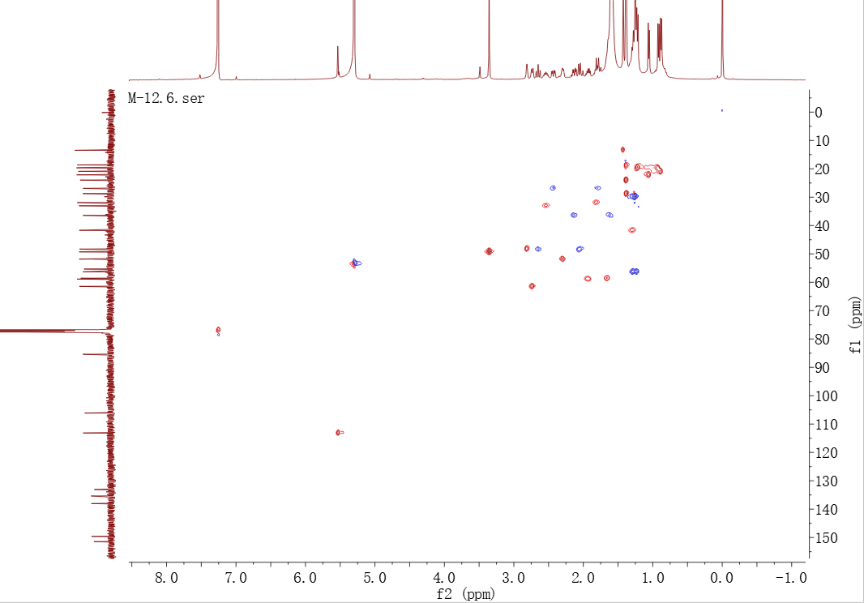


Figure S14. HMBC spectrum of **2**.


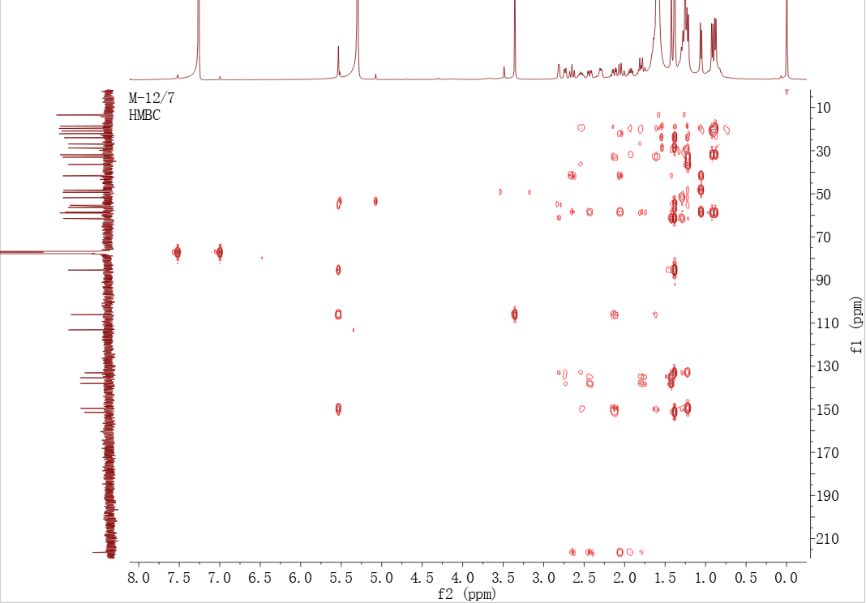


Figure S15. NOESY spectrum of **2**.


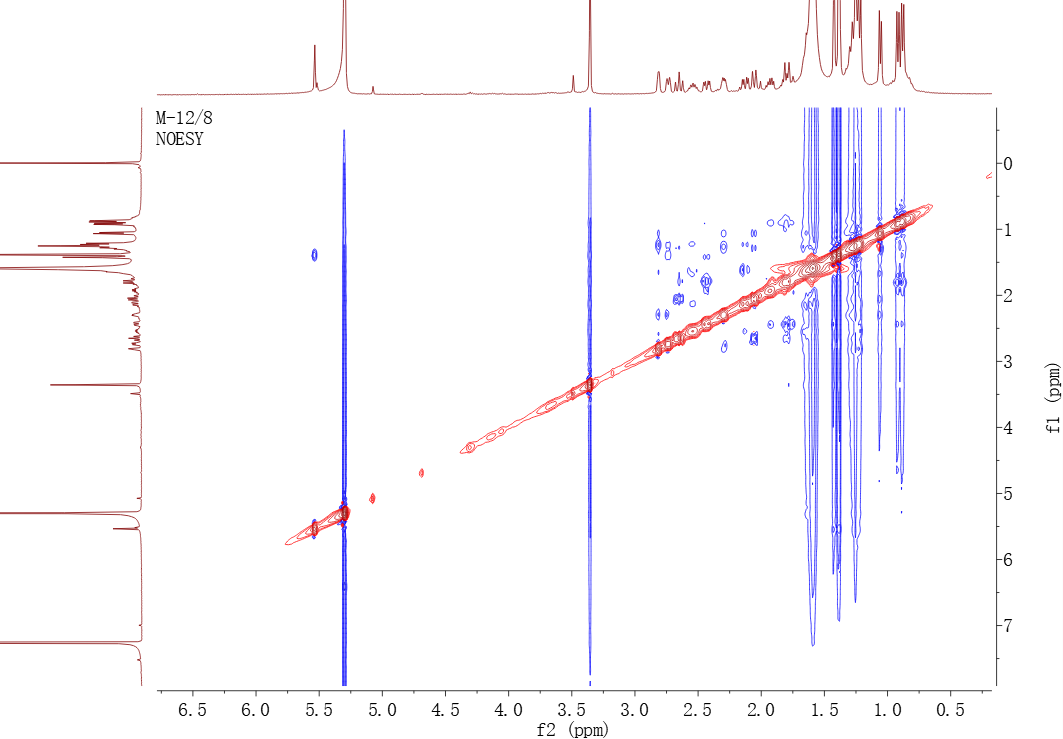


Figure S16. HR-ESI-MS spectrum of **2**.


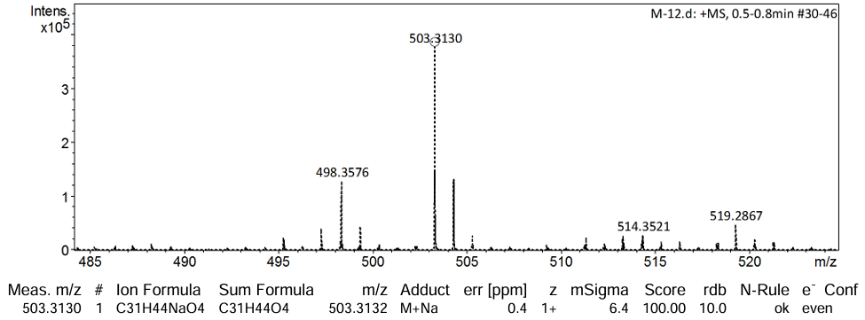


Figure S17. ^1^H NMR spectrum of **3**.


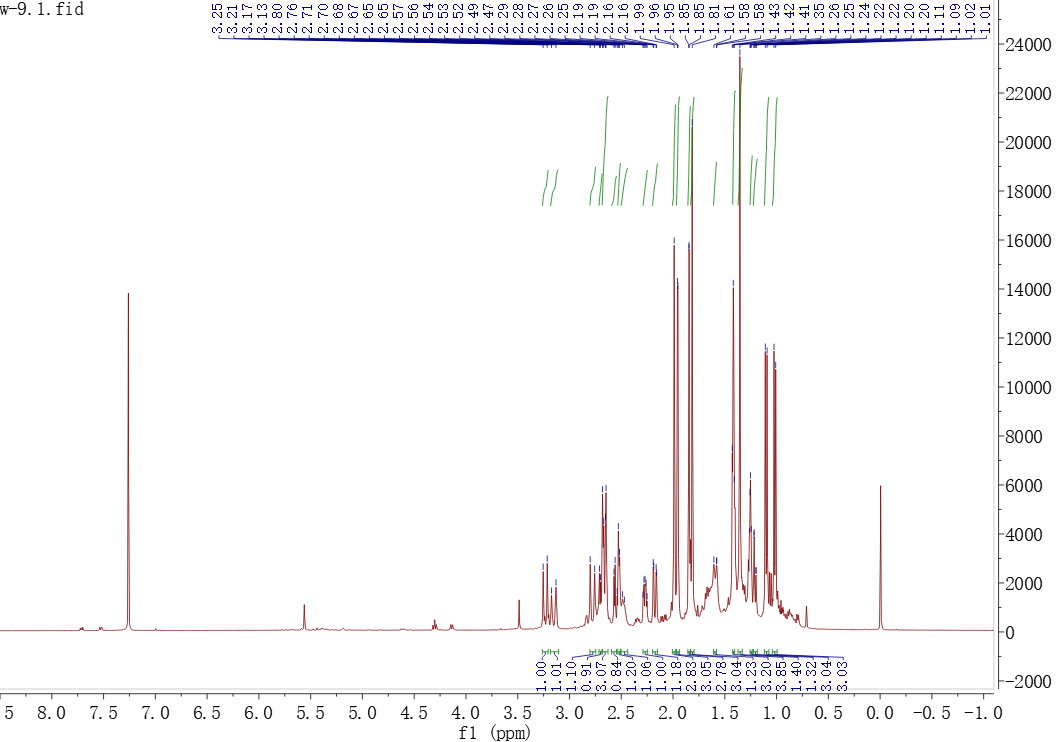


Figure S18. ^13^C NMR spectrum of **3**.


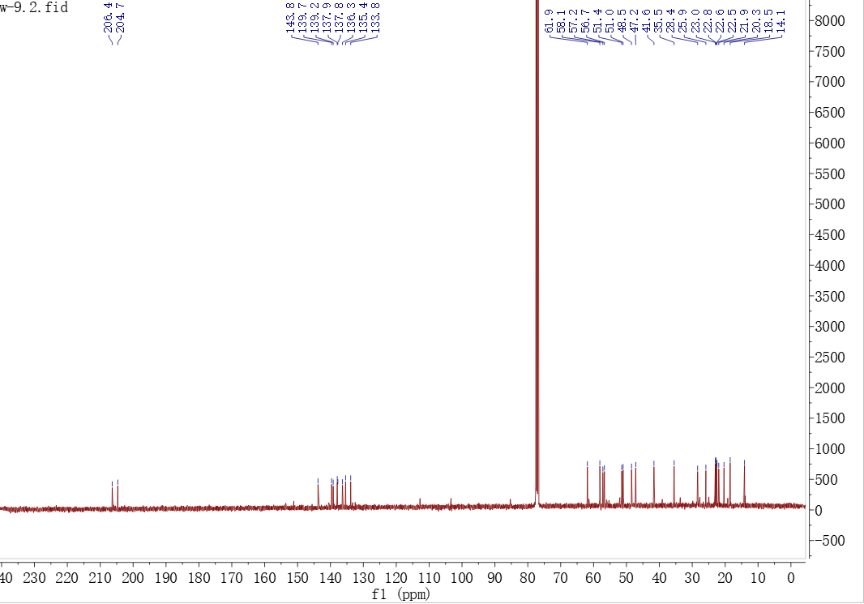


Figure S19. DEPT spectrum of **3**.


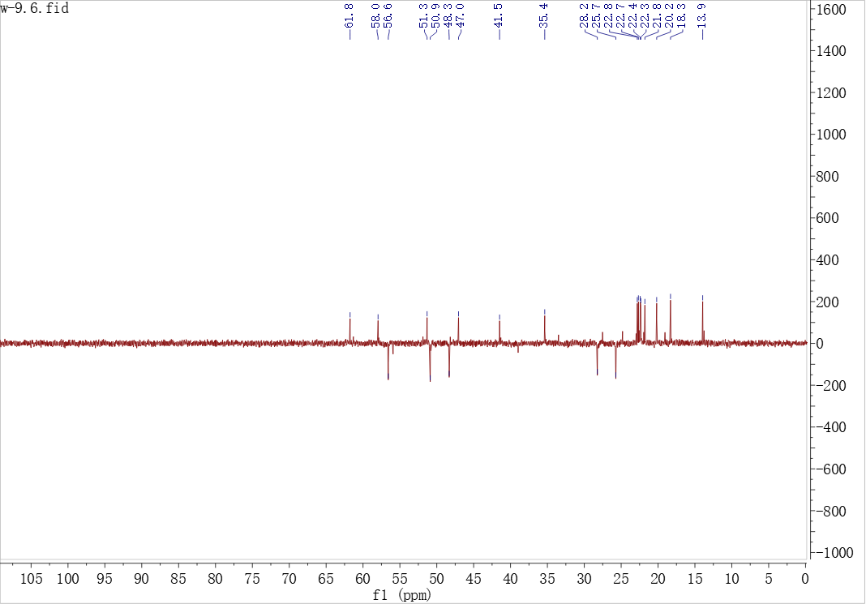


Figure S20. ^1^H-^1^H COSY spectrum of **3**.


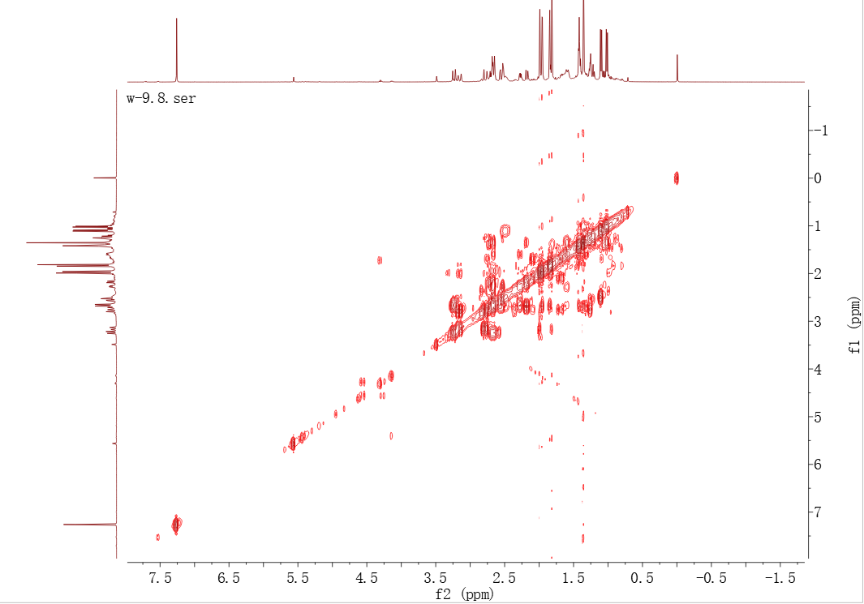


Figure S21. HSQC spectrum of **3**.


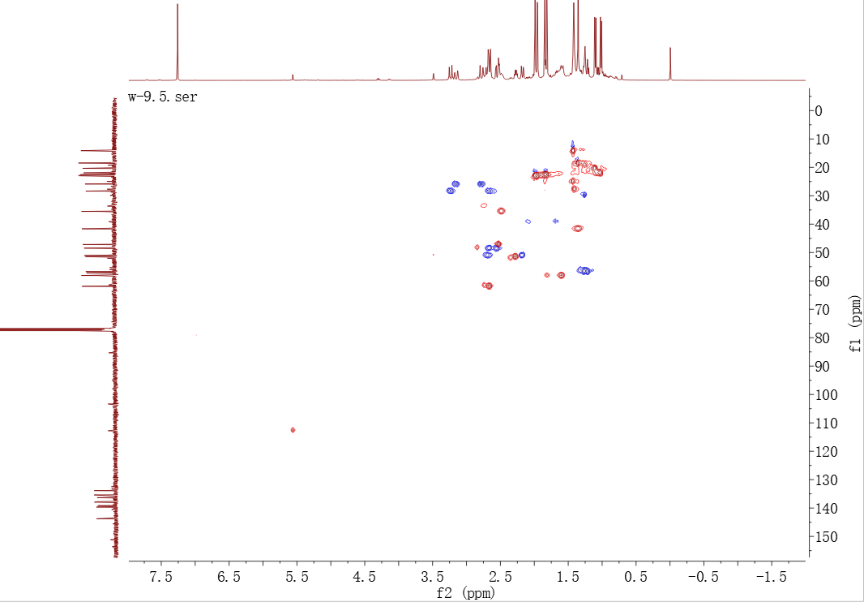


Figure S22. HMBC spectrum of **3**.


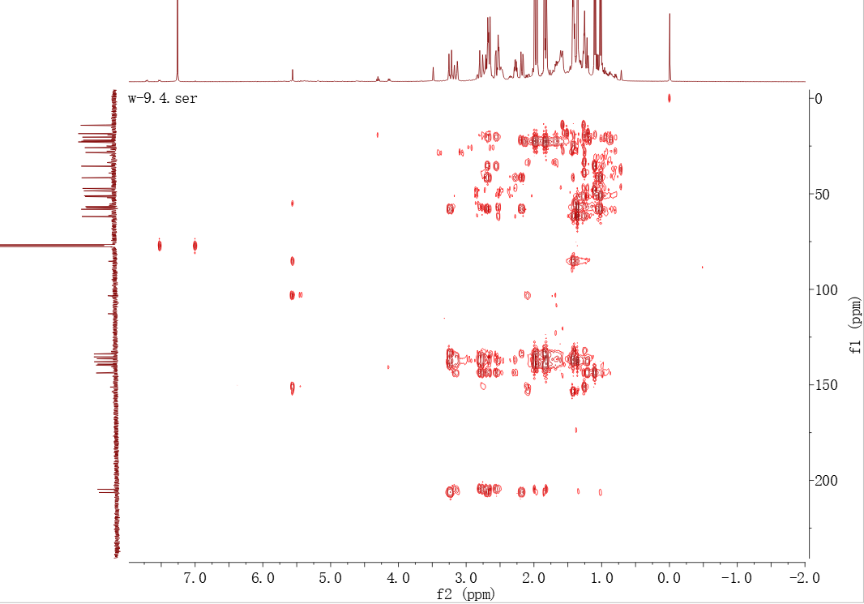


Figure S23. NOESY spectrum of **3**.


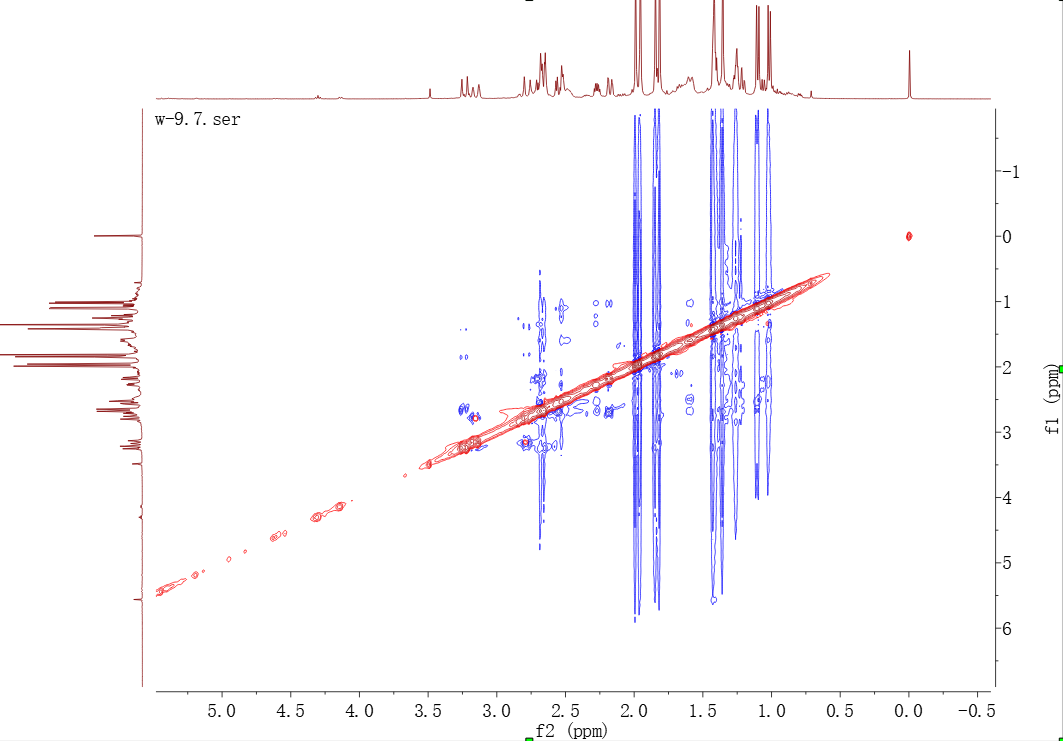


Figure S24. HR-ESI-MS spectrum of **3**.


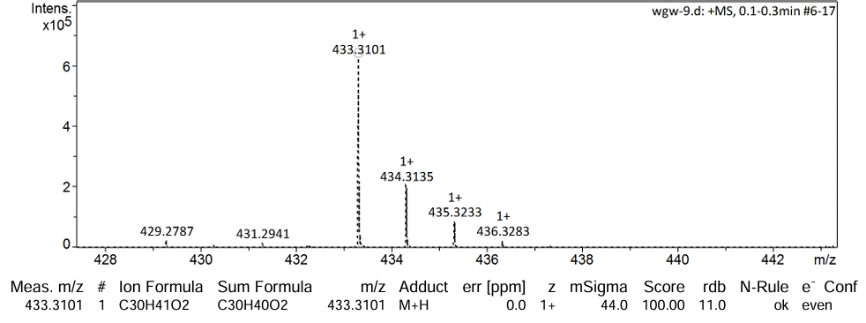


Figure S25. ^1^H NMR spectrum of **4**.


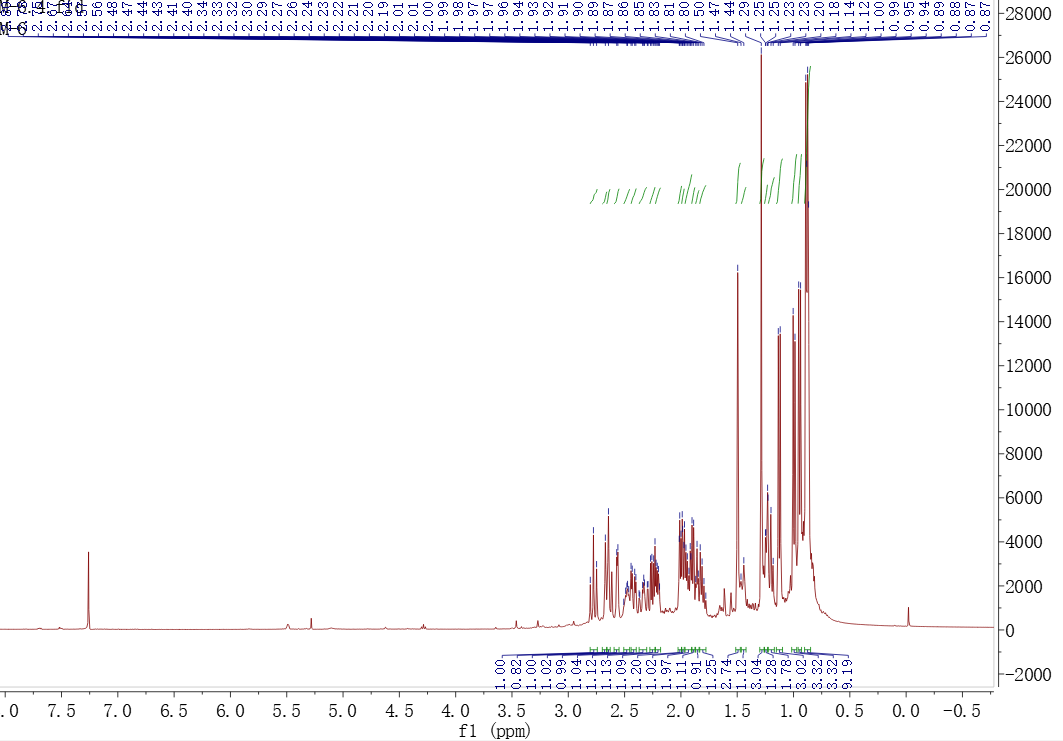


Figure S26. ^13^C NMR spectrum of **4**.


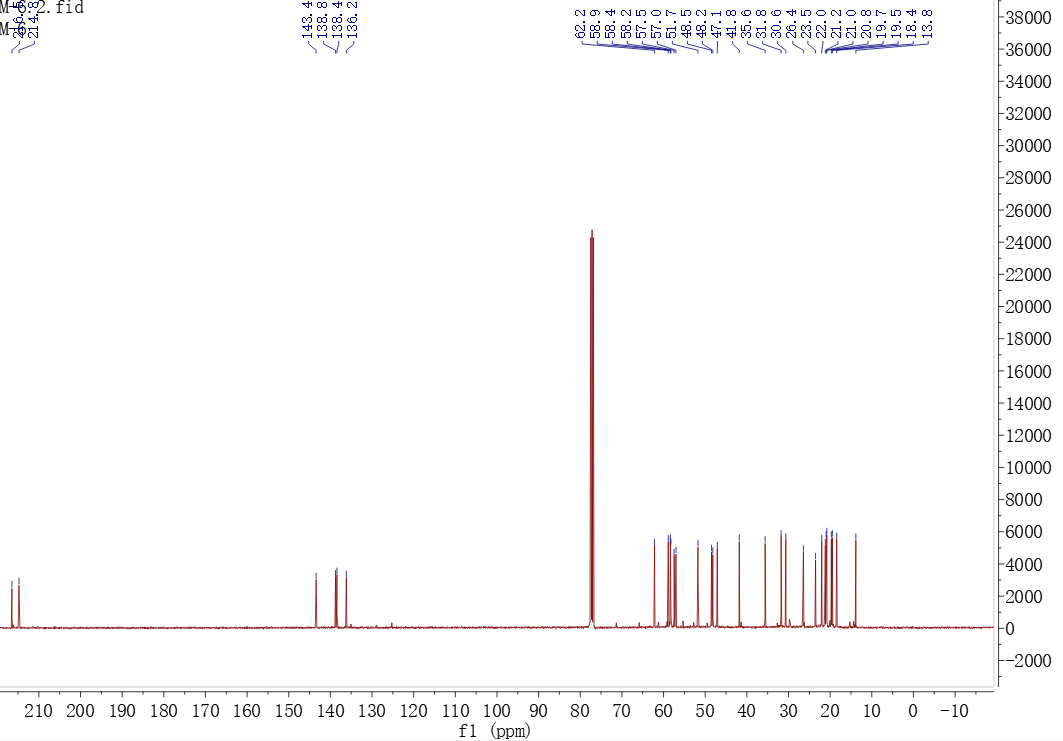


Figure S27. DEPT spectrum of **4**.


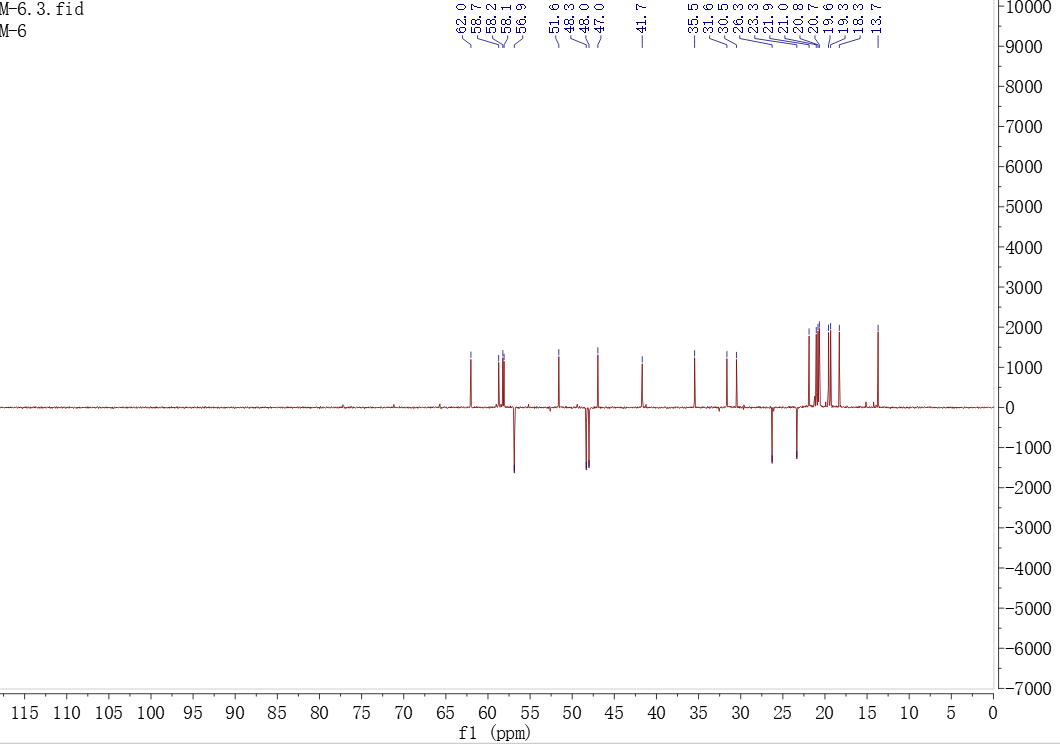


Figure S28. ^1^H-^1^H COSY spectrum of **4**.


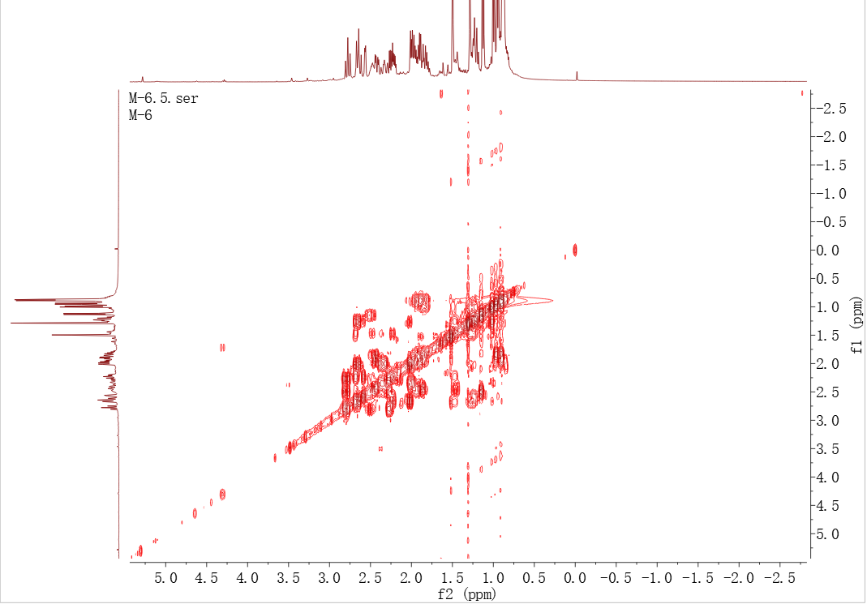


Figure S29. HSQC spectrum of **4**.


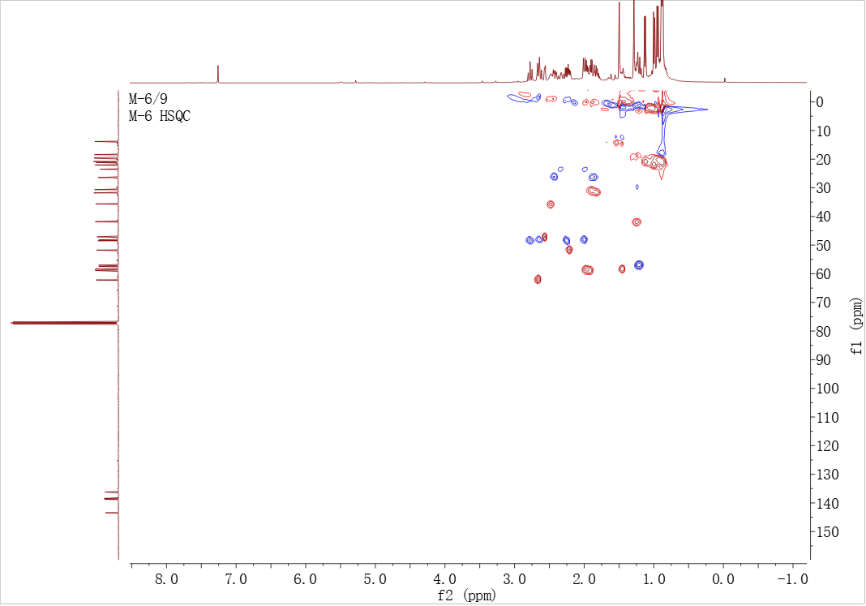


Figure S30. HMBC spectrum of **4**.


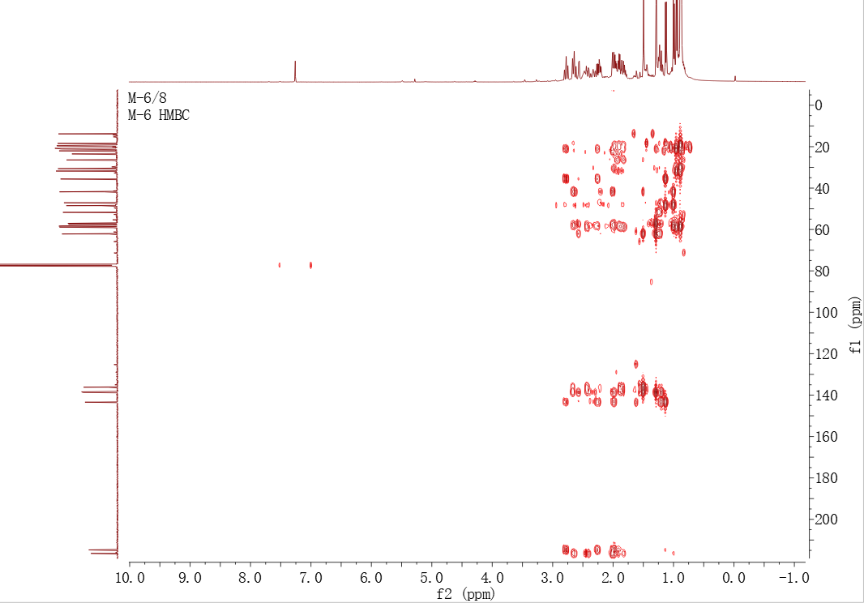


Figure S31. NOESY spectrum of **4**.


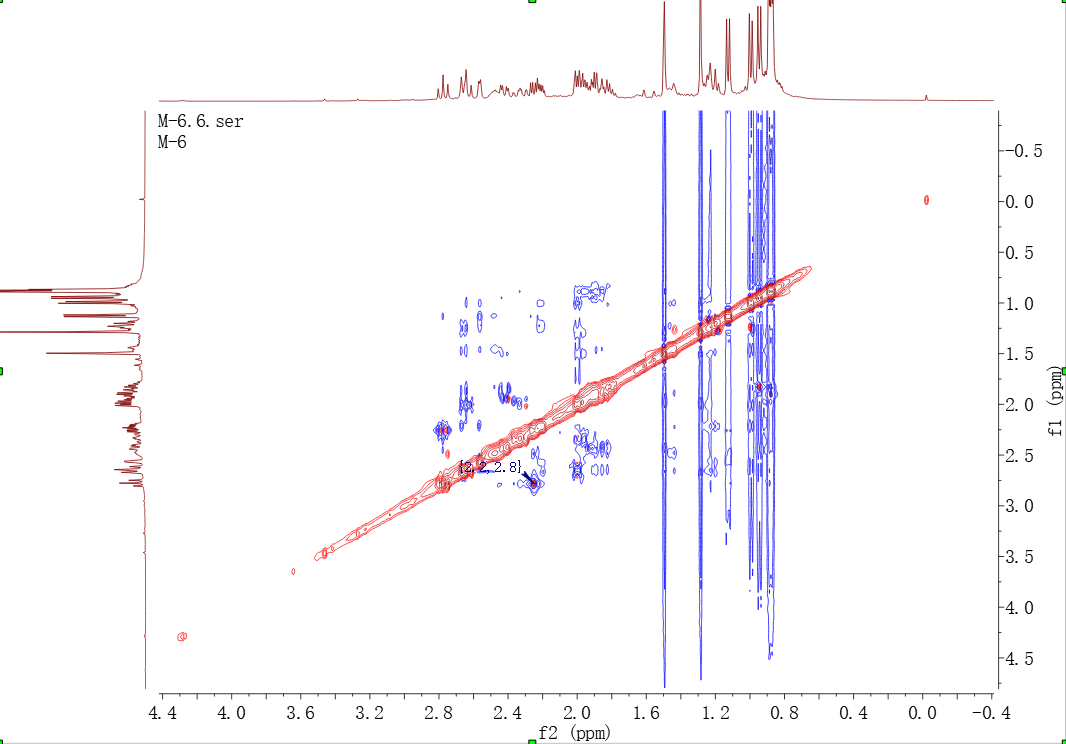


Figure S32. HR-ESI-MS spectrum of **4**.


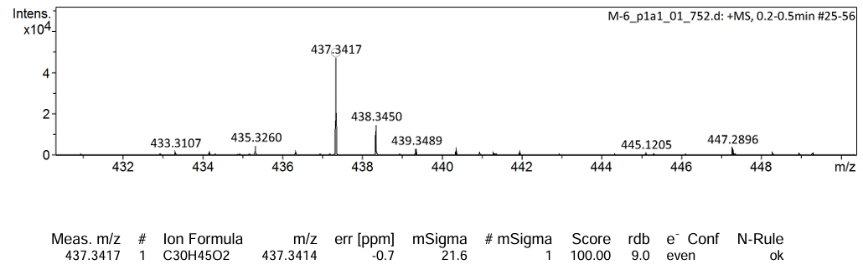


Figure S33. ^1^H NMR spectrum of **5**.


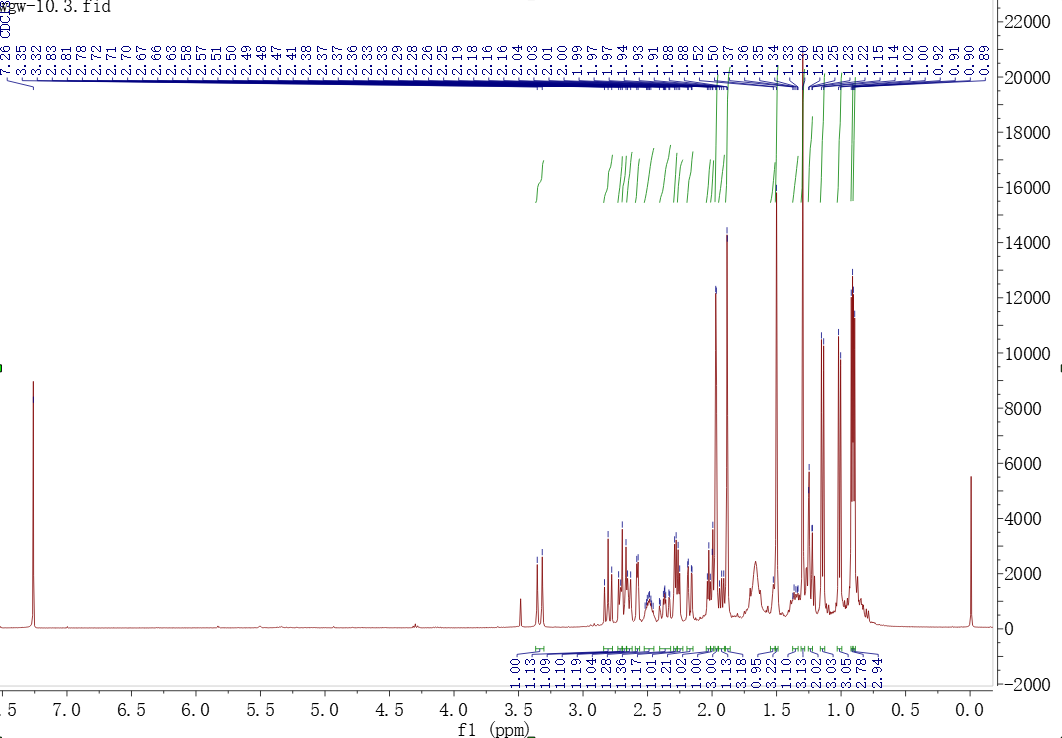


Figure S34. ^13^C NMR spectrum of **5**.


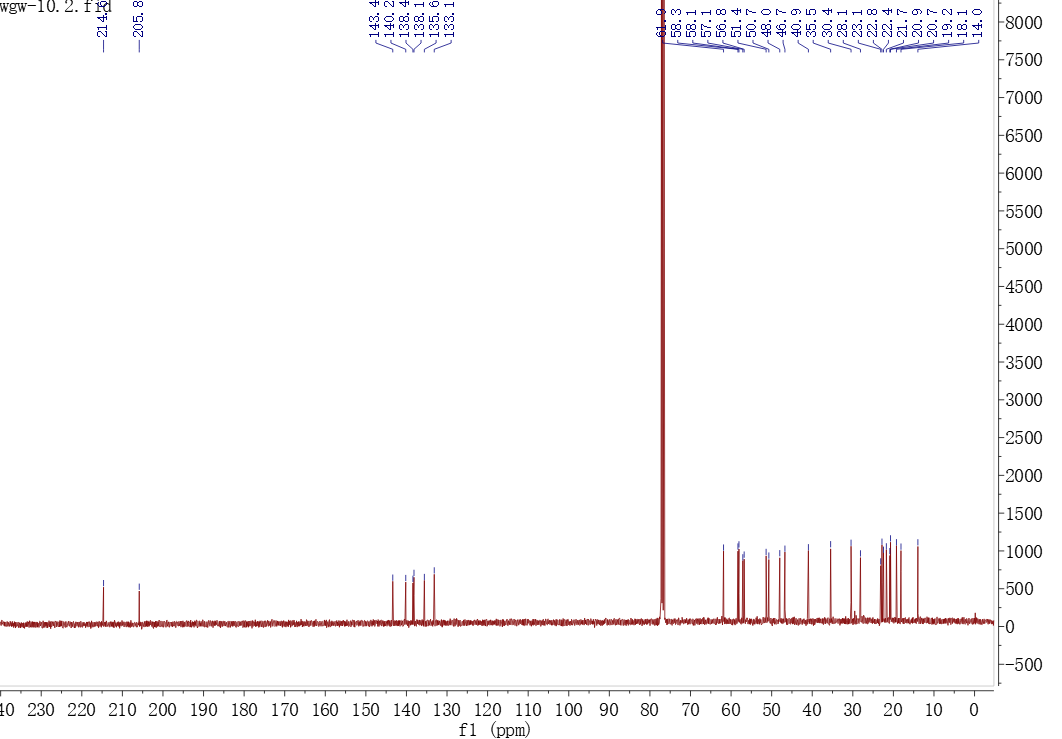


Figure S35. DEPT spectrum of **5**.


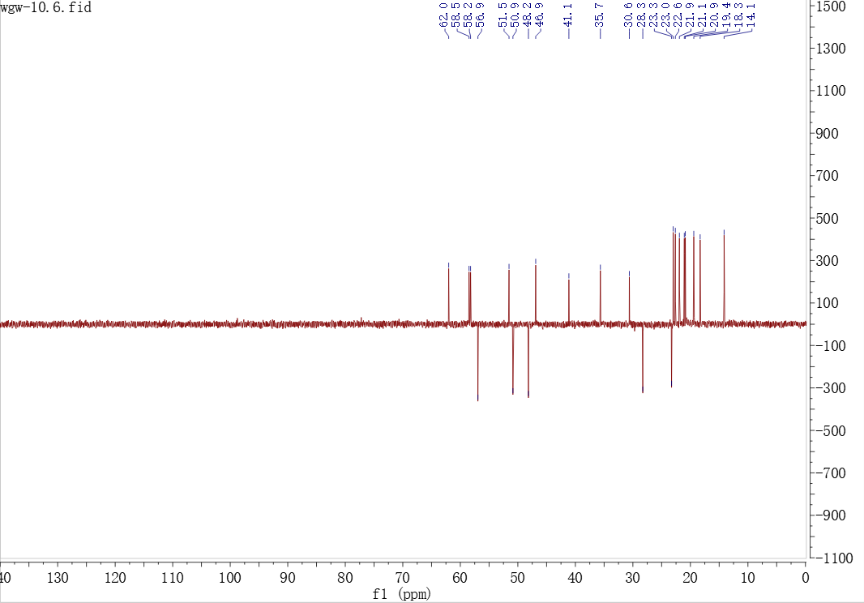


Figure S36. ^1^H-^1^H COSY spectrum of **5**.


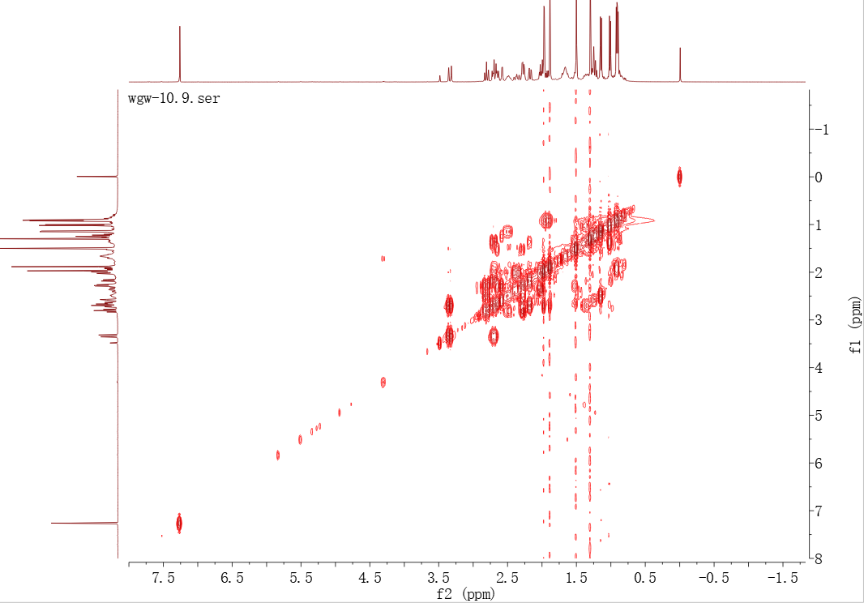


Figure S37. HSQC spectrum of **5**.


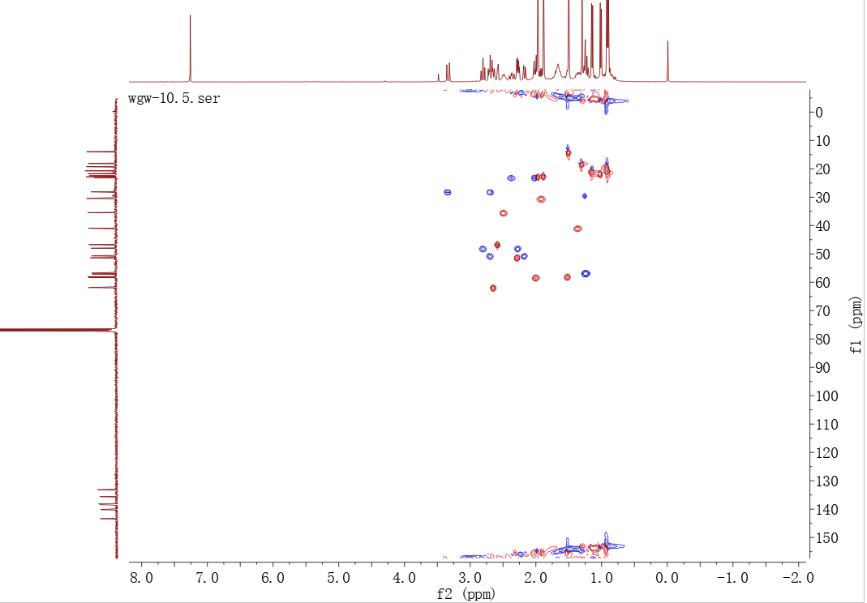


Figure S38. HMBC spectrum of **5**.


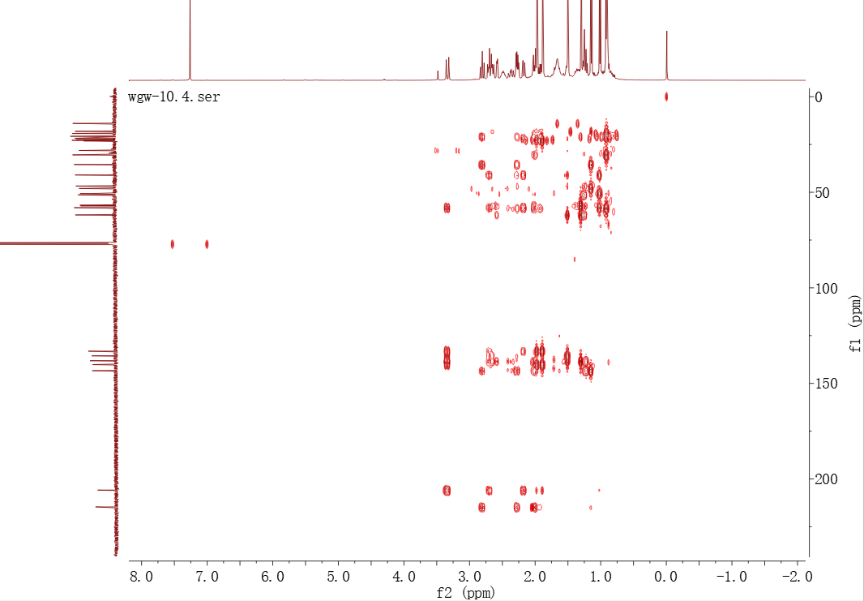


Figure S39. NOESY spectrum of **5**.


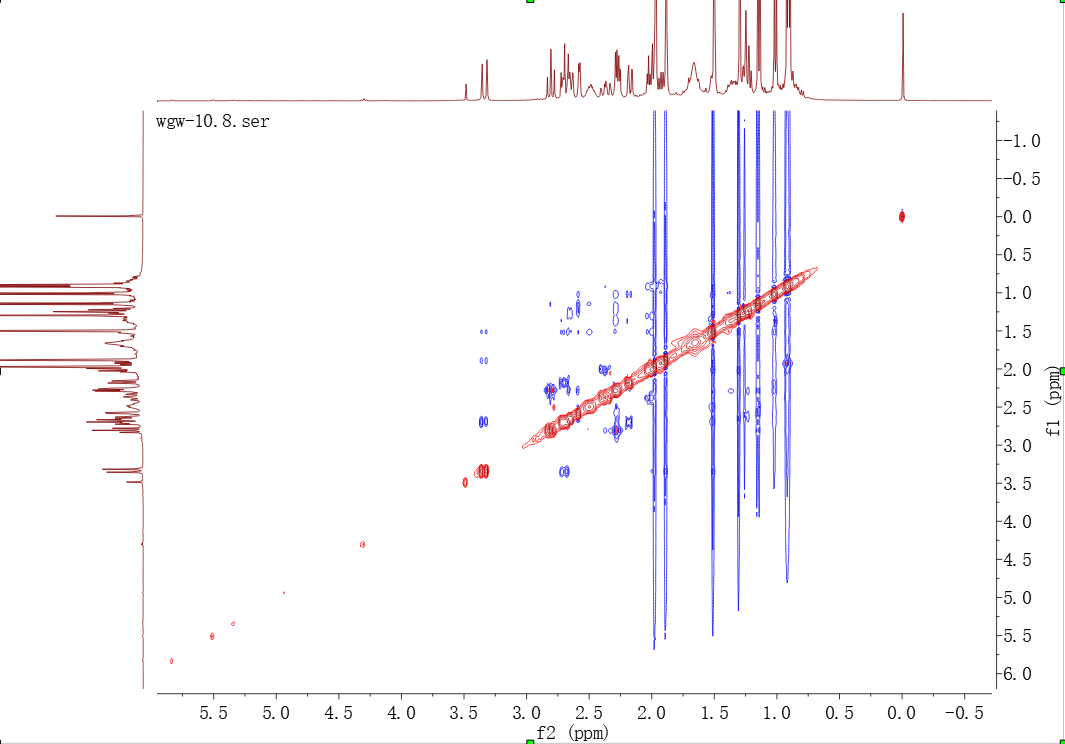


Figure S40. HR-ESI-MS spectrum of **5**.


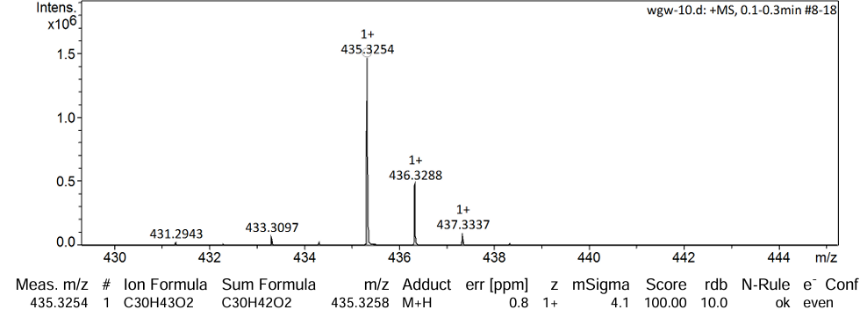


Figure S41. ^1^H NMR spectrum of **6**.


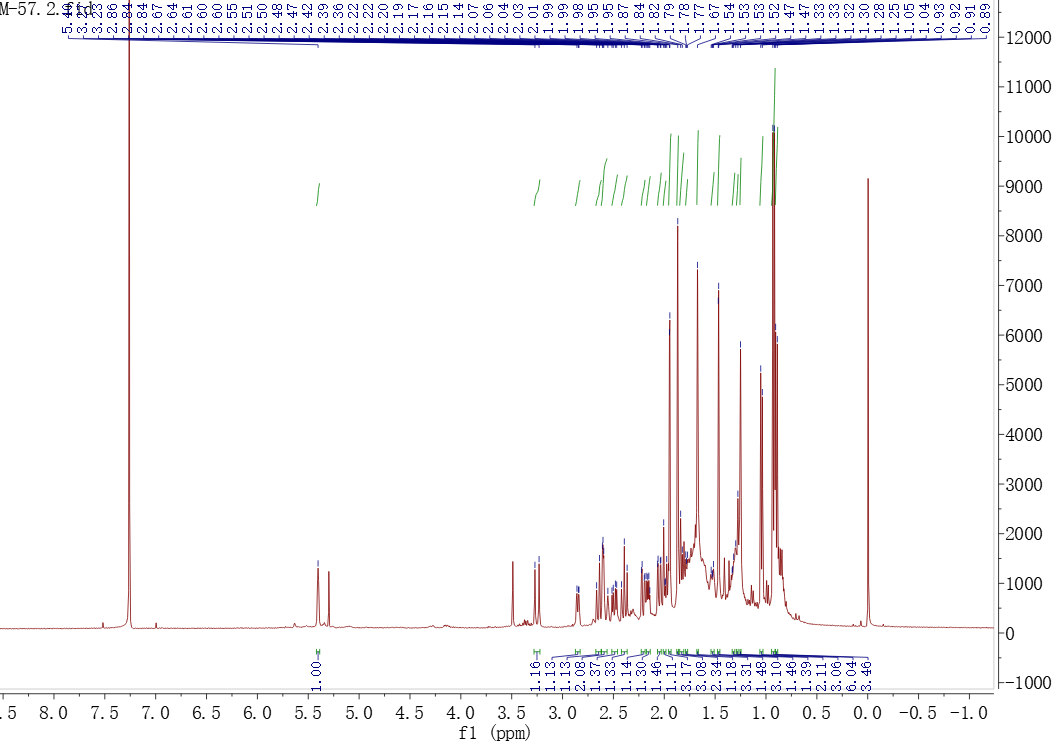


Figure S42. ^13^C NMR spectrum of **6**.


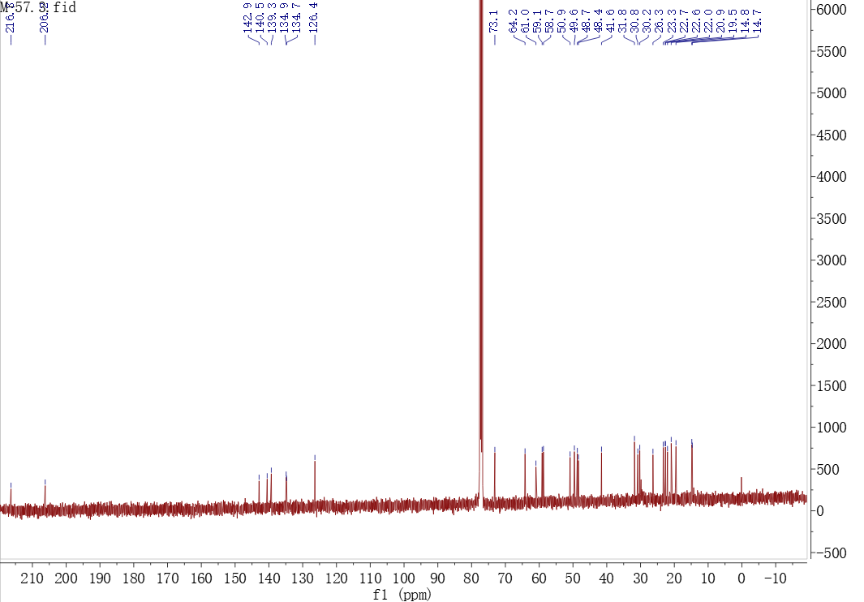


Figure S43. DEPT spectrum of **6**.


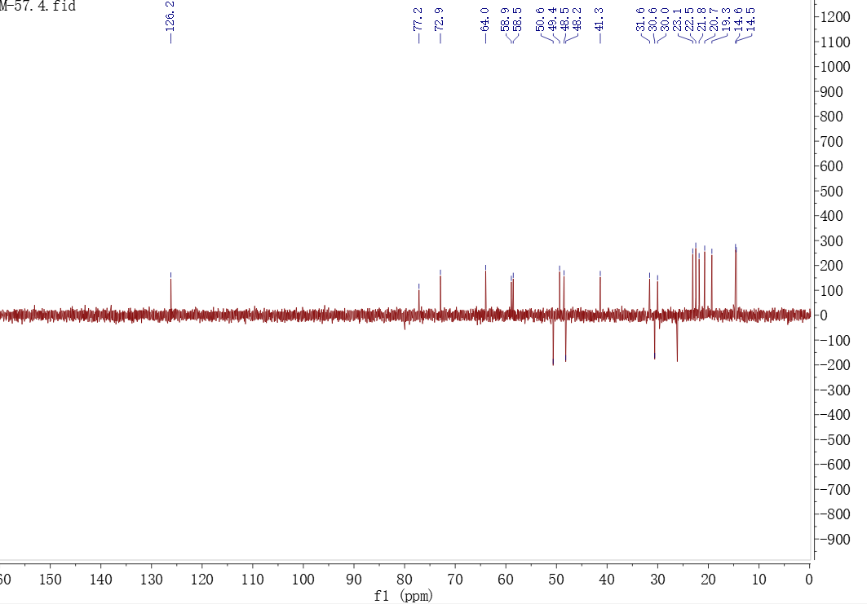


Figure S44. ^1^H-^1^H COSY spectrum of **6**.


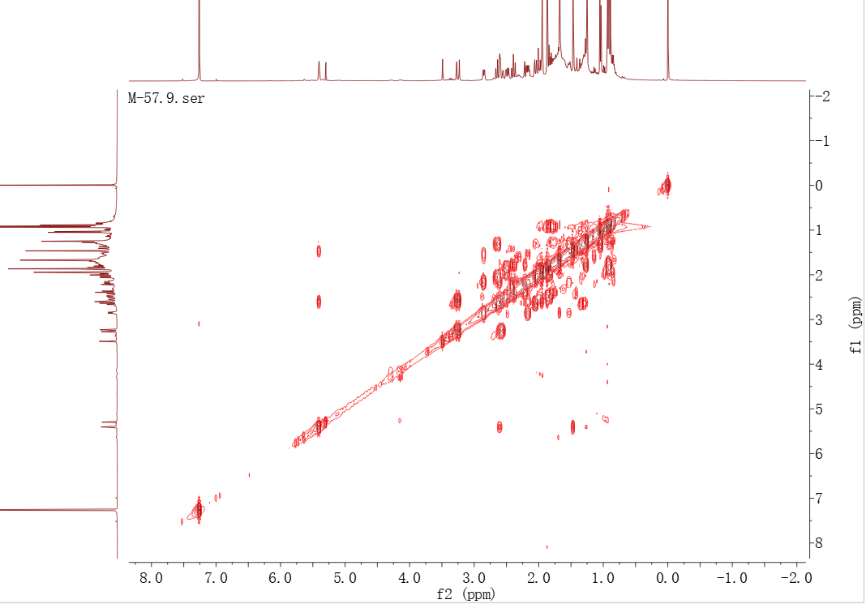


Figure S45. HSQC spectrum of **6**.


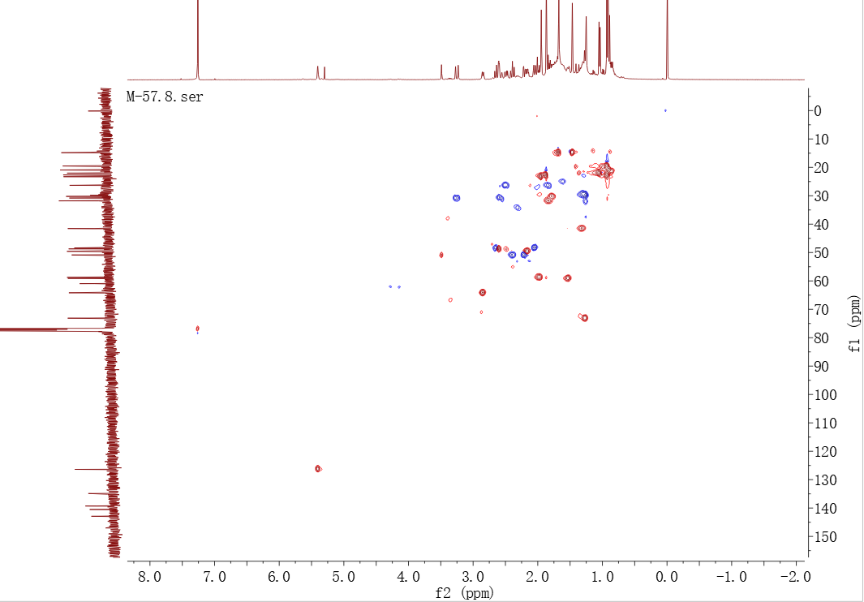


Figure S46. HMBC spectrum of **6**.


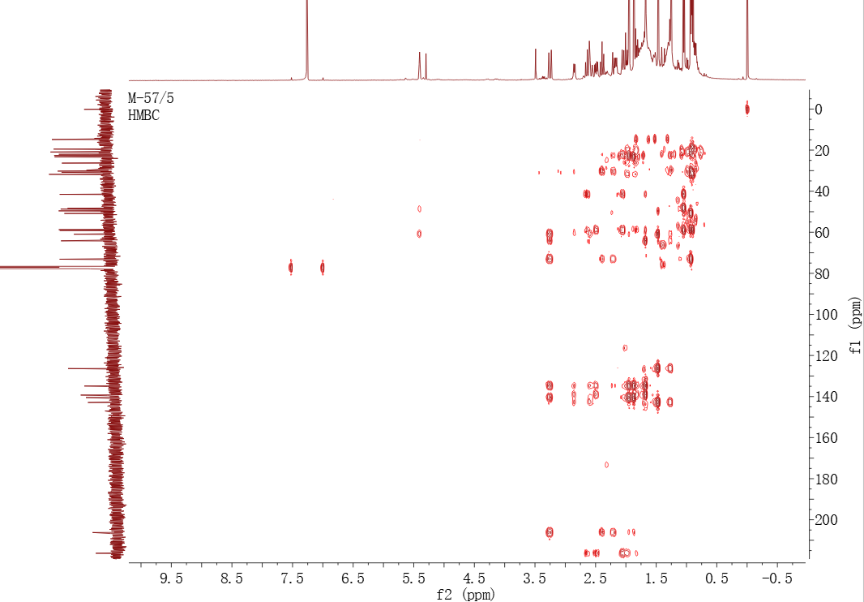


Figure S47. NOESY spectrum of **6**.


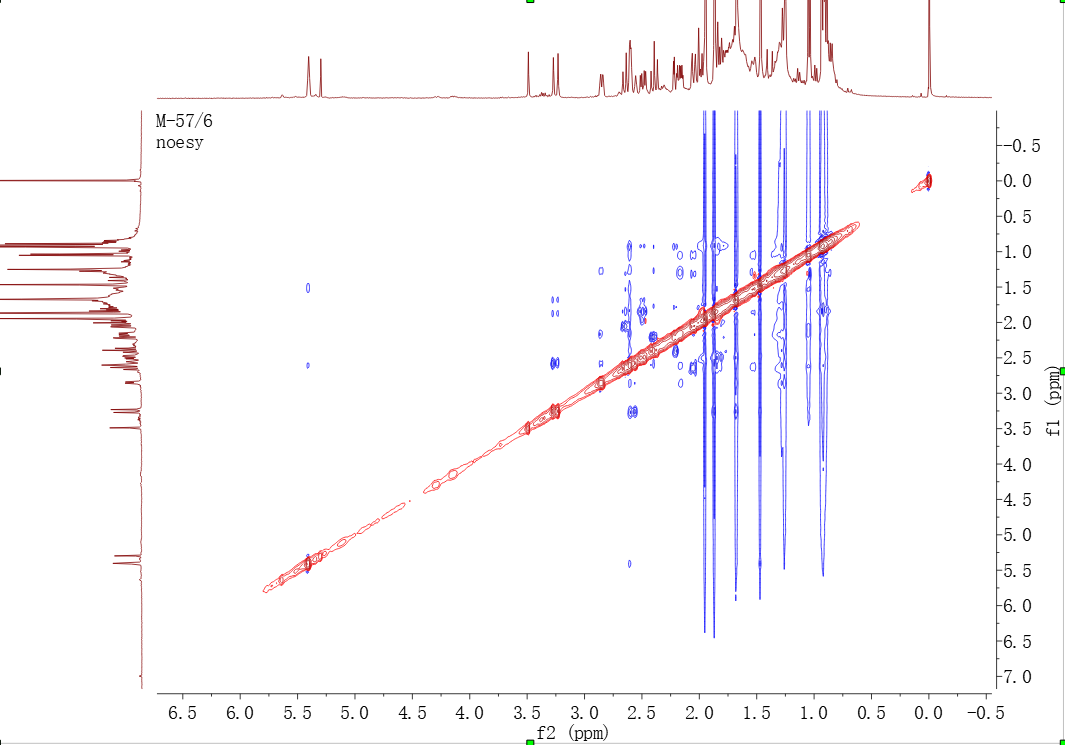


Figure S48. HR-ESI-MS spectrum of **6**.


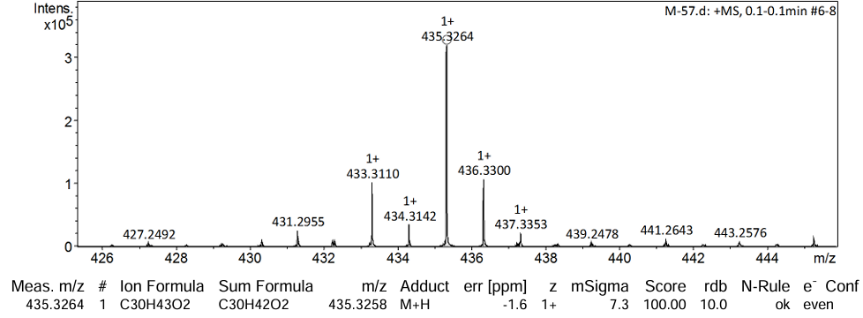


Figure S49. ^1^H NMR spectrum of **7**.


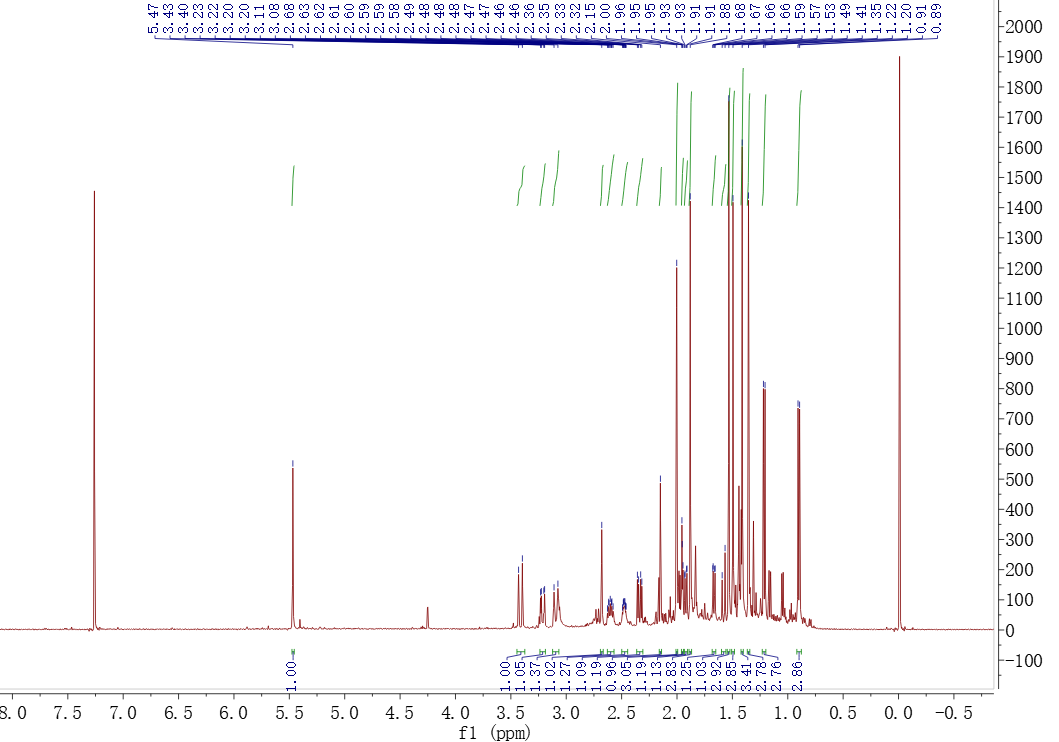


Figure S50. ^13^C NMR spectrum of **7**.


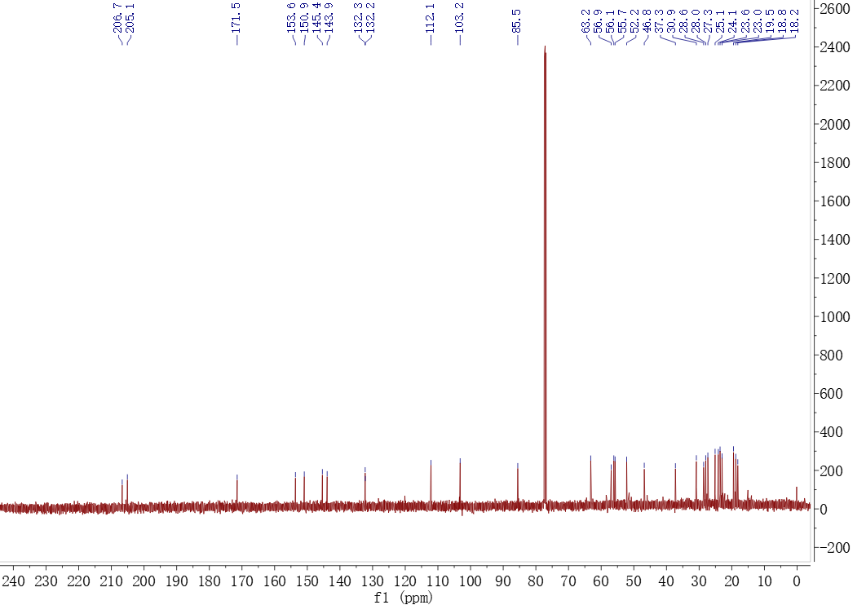


Figure S51. DEPT spectrum of **7**.


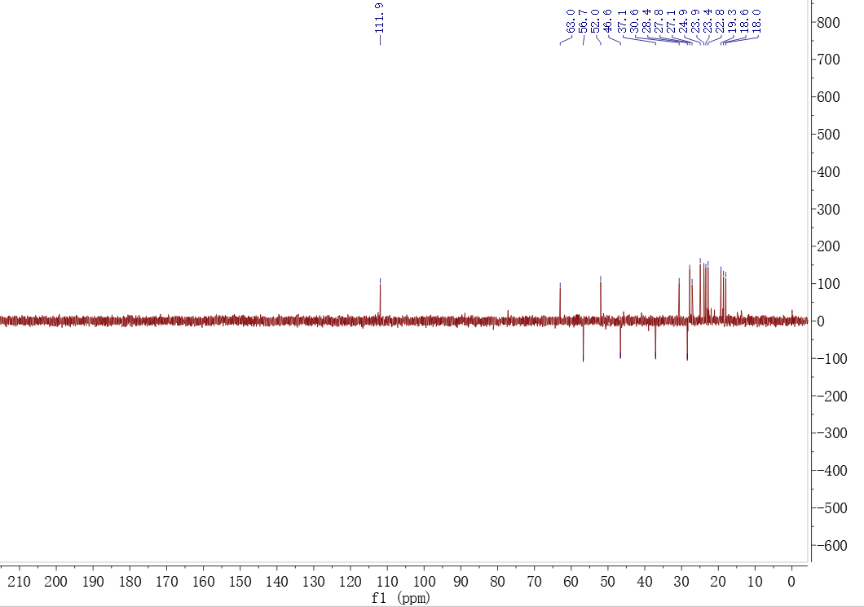


Figure S52. ^1^H-^1^H COSY spectrum of **7**.


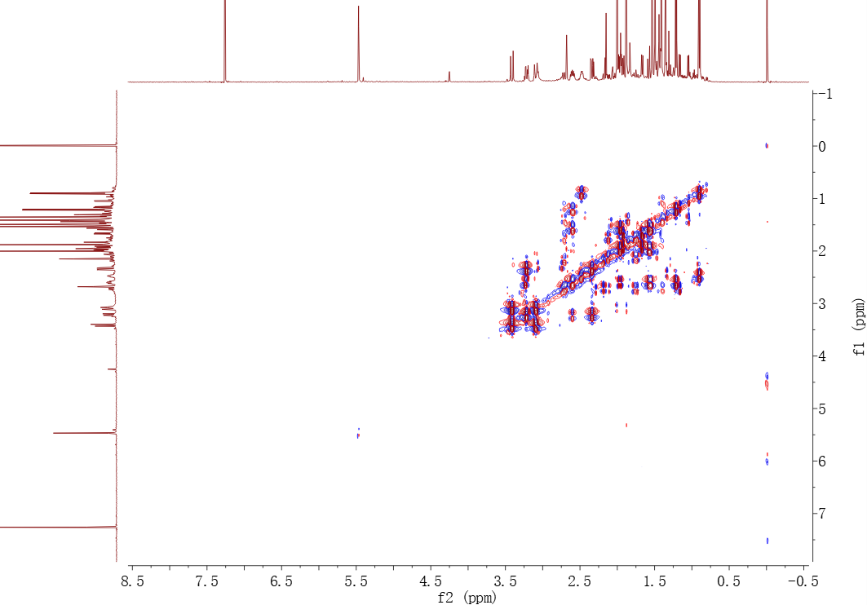


Figure S53. HSQC spectrum of **7**.


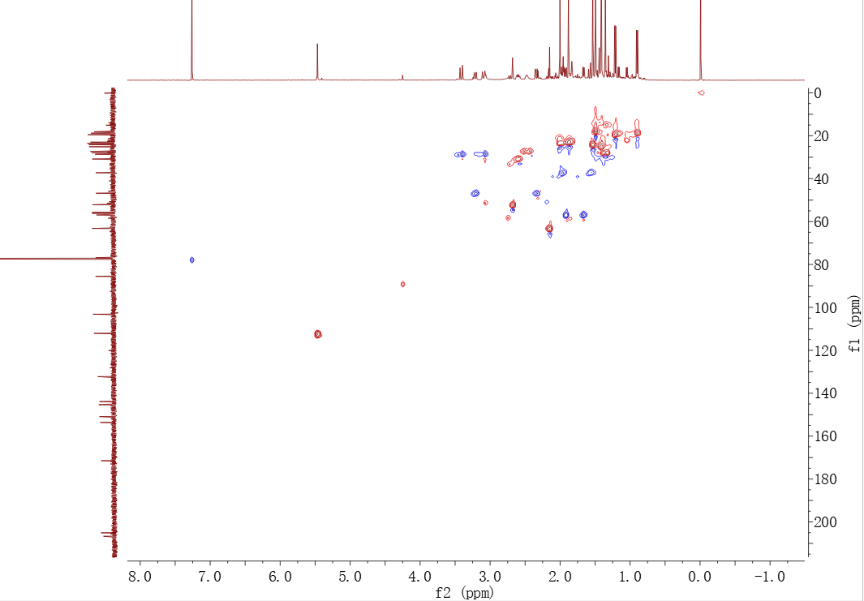


Figure S54. HMBC spectrum of **7**.


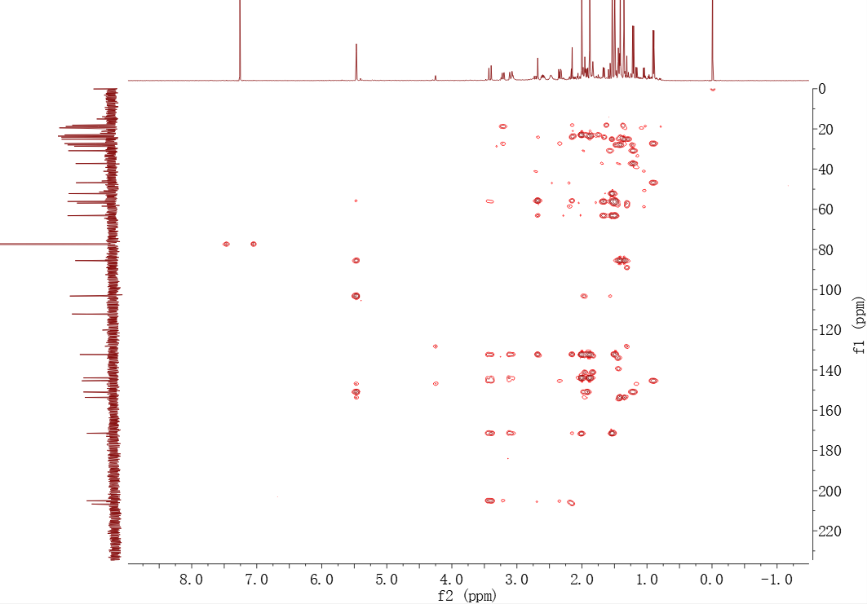


Figure S55. NOESY spectrum of **7**.


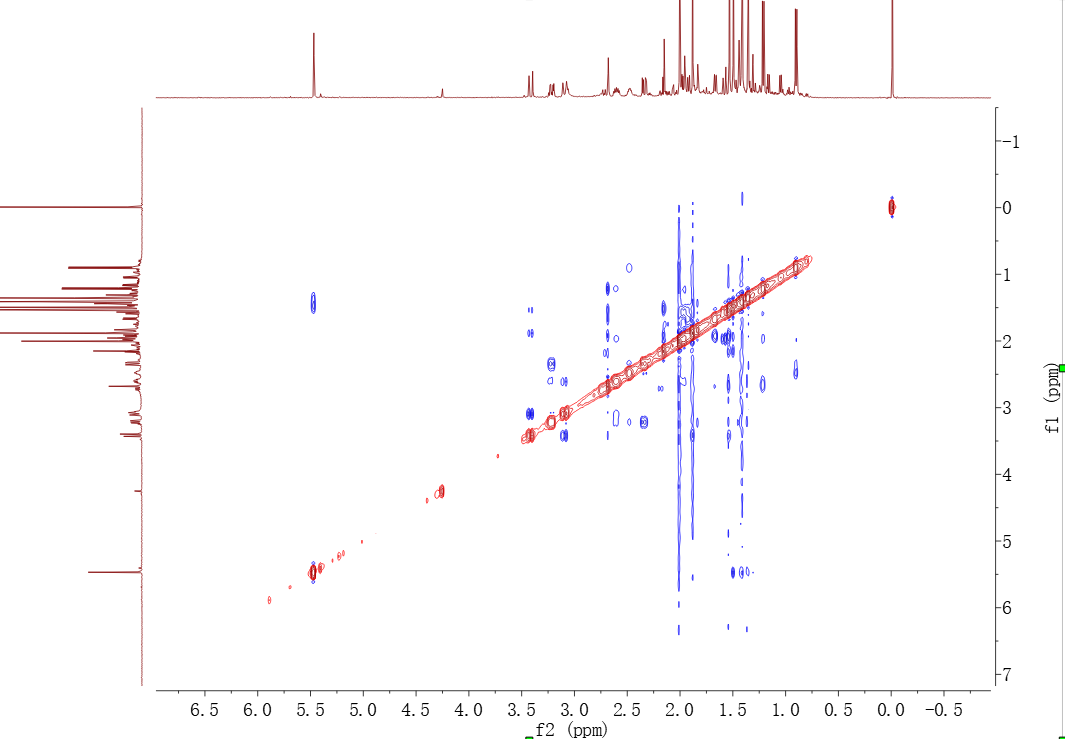


Figure S56. HR-ESI-MS spectrum of **7**.


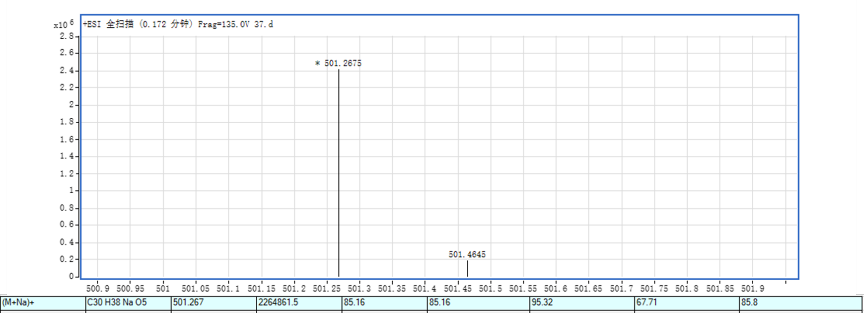


Figure S57. ^1^H NMR spectrum of **8**.


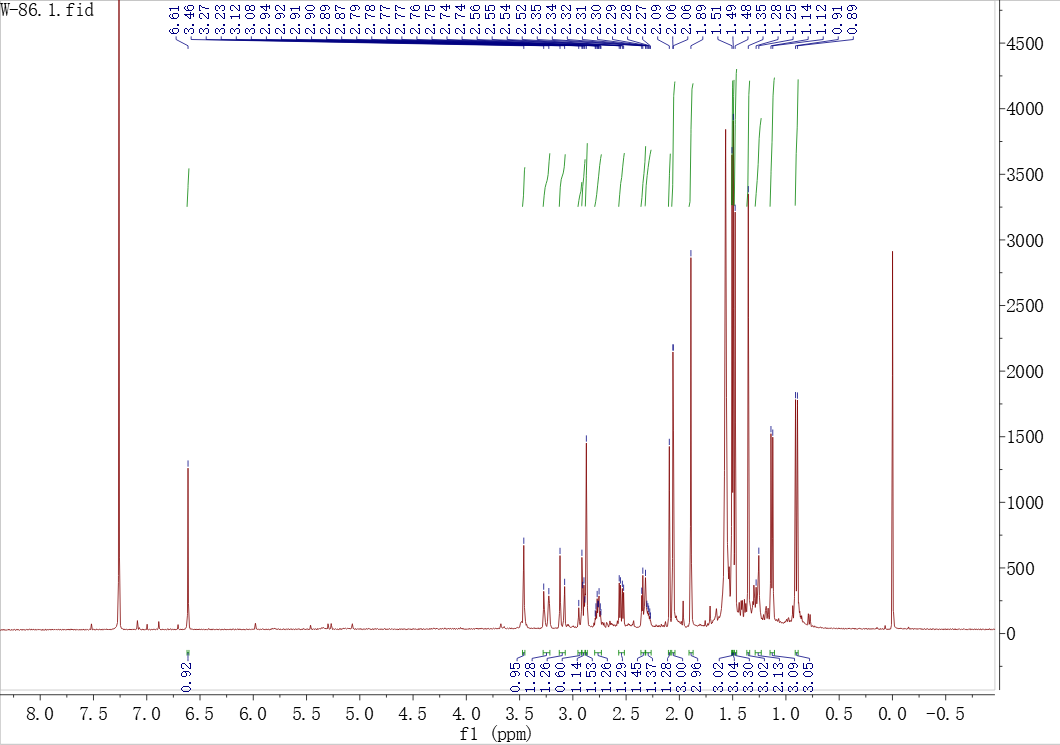


Figure S58. ^13^C NMR spectrum of **8**.


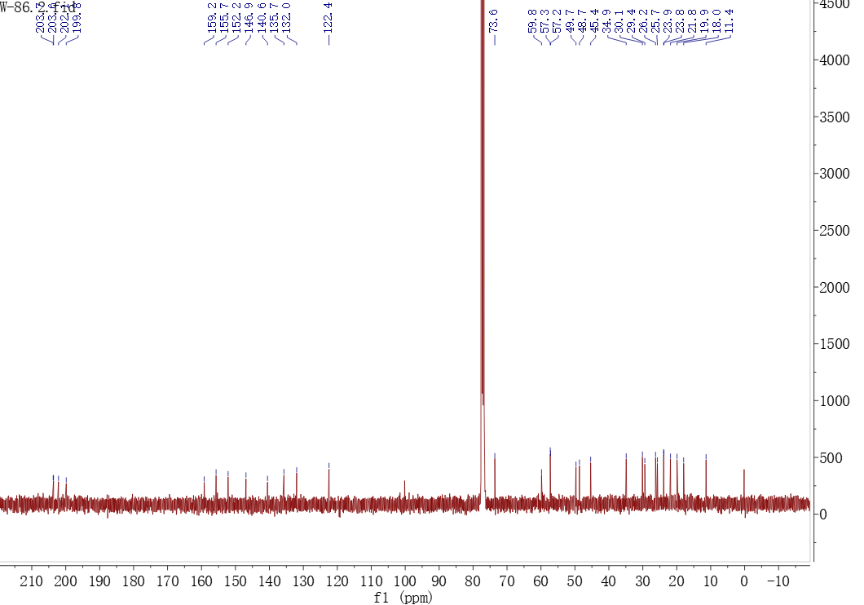


Figure S59. DEPT spectrum of **8**.


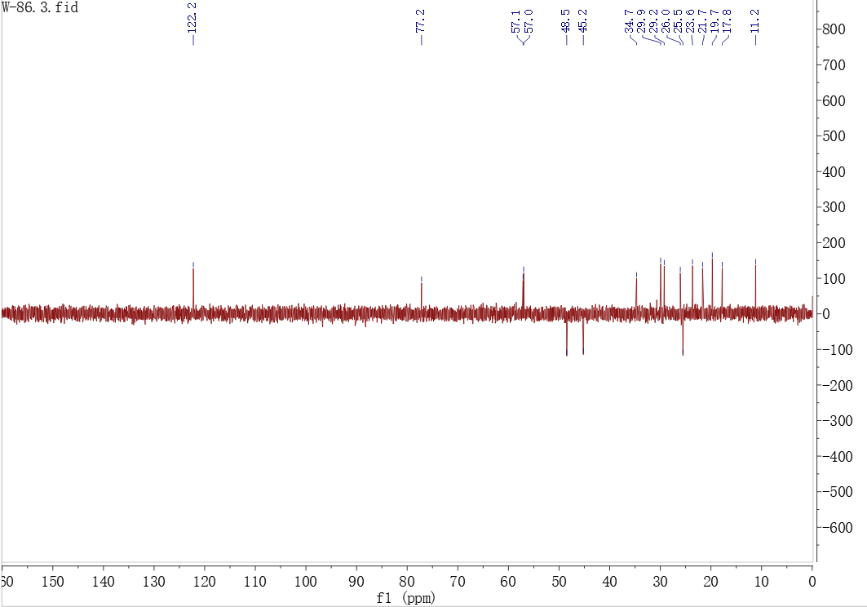


Figure S60. ^1^H-^1^H COSY spectrum of **8**.


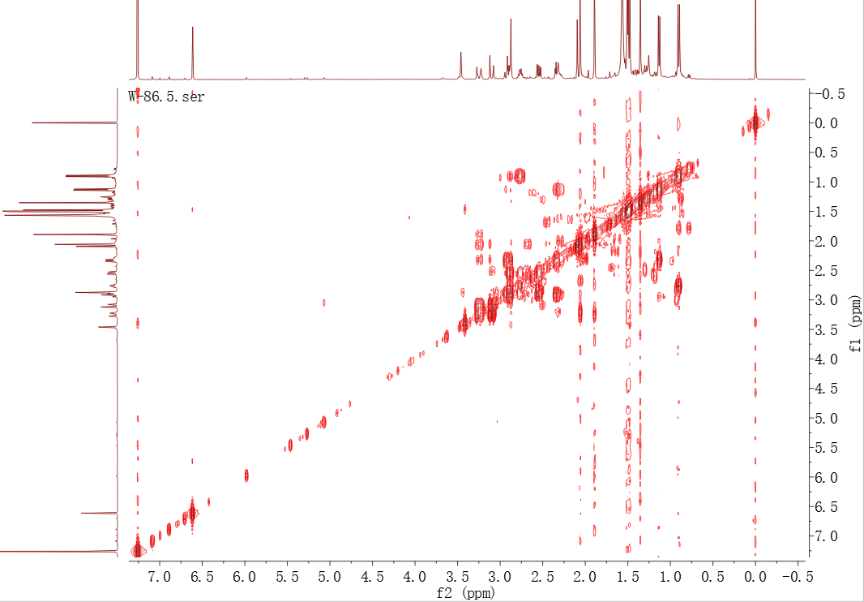


Figure S61. HSQC spectrum of **8**.


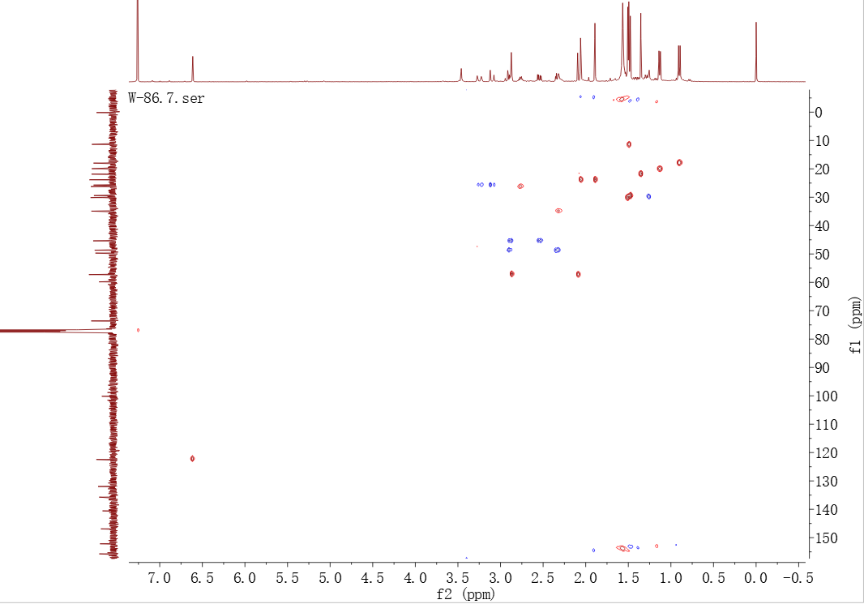


Figure S62. HMBC spectrum of **8**.


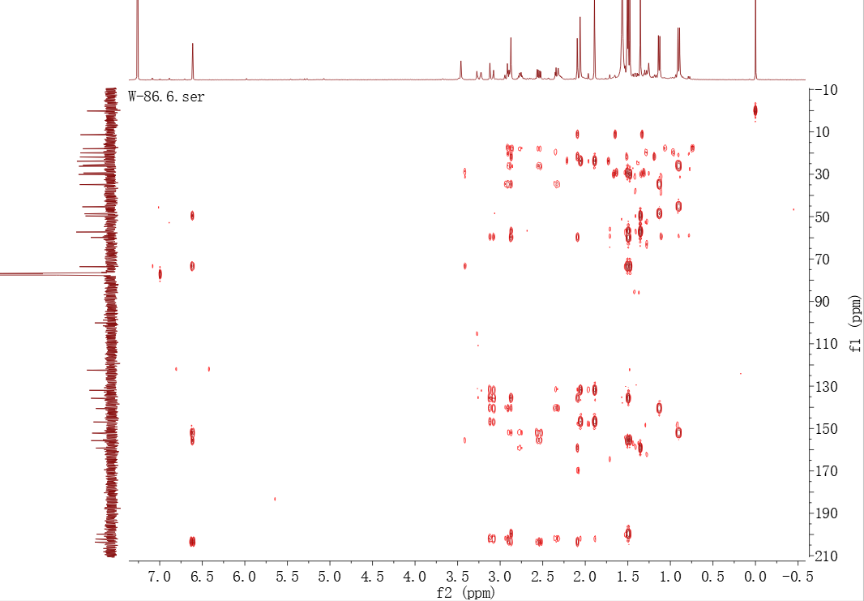


Figure S63. NOESY spectrum of **8**.


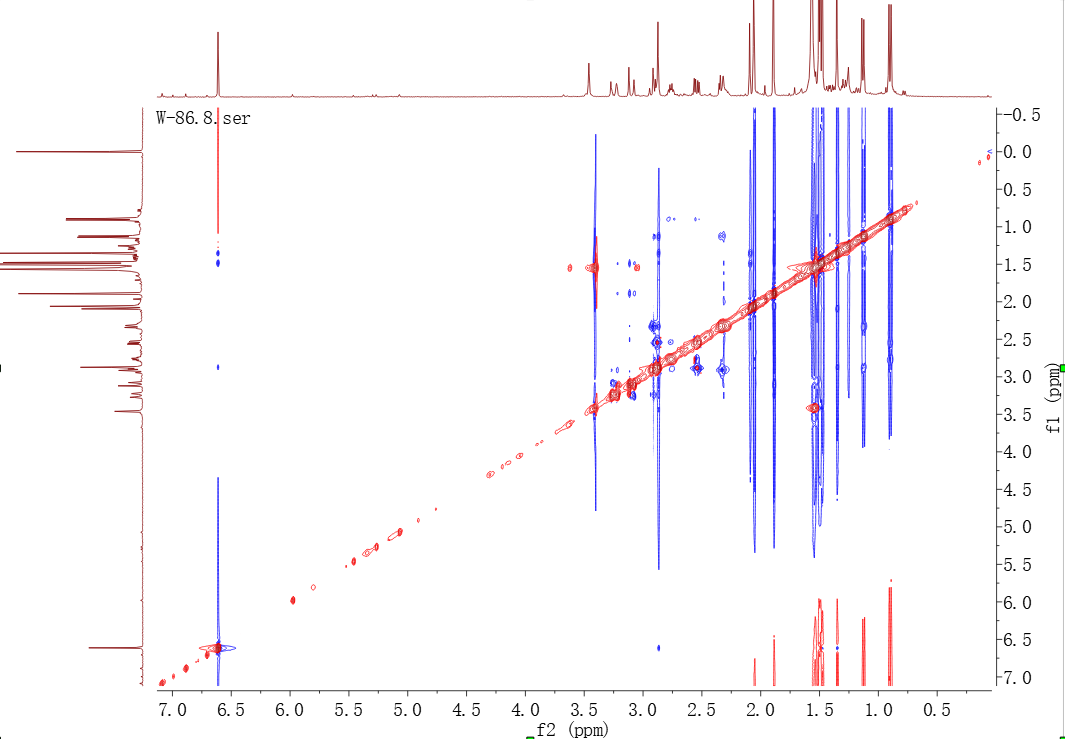


Figure S64. HR-ESI-MS spectrum of **8**.


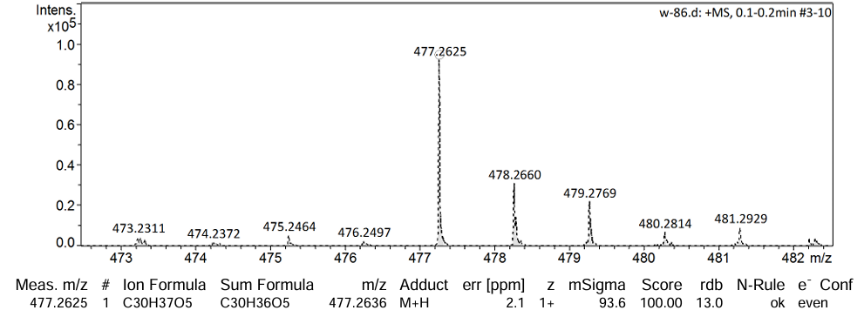


Figure S65. ^1^H NMR spectrum of **9**.


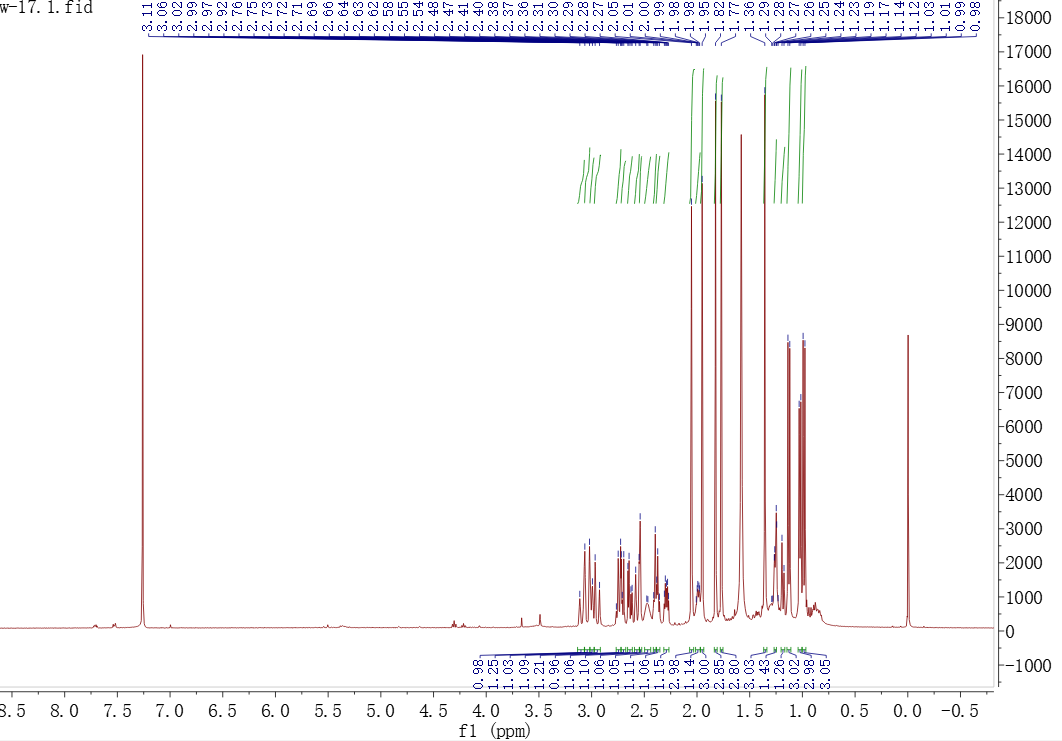


Figure S66. ^13^C NMR spectrum of **9**.


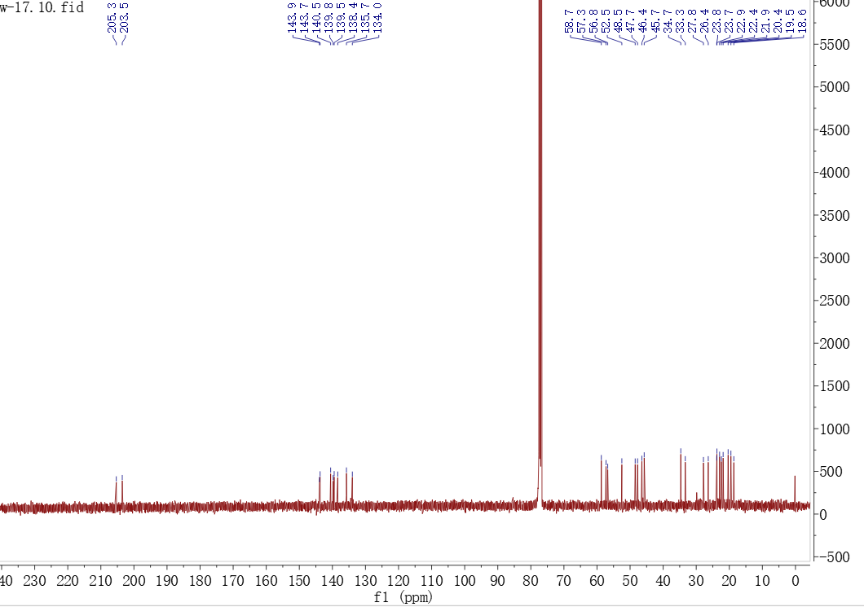


Figure S67. DEPT spectrum of **9**.


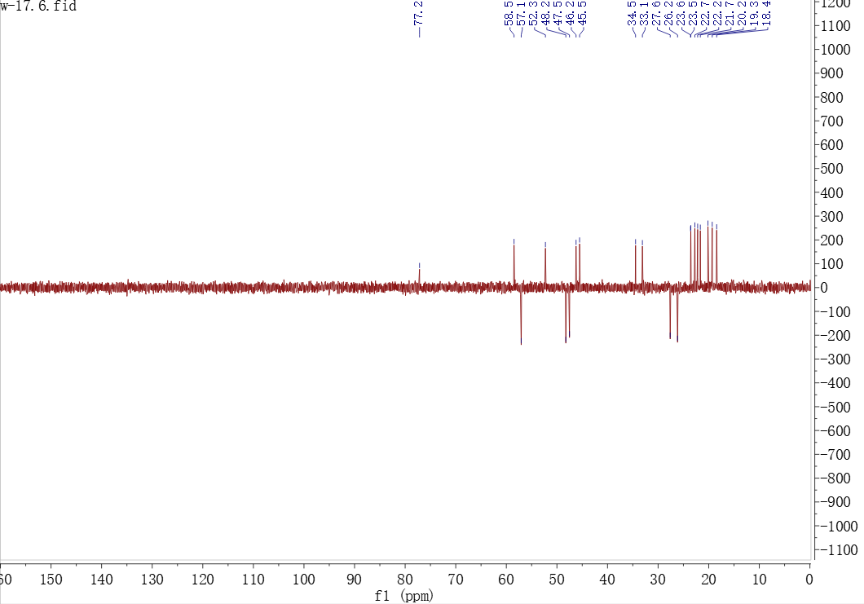


Figure S68. ^1^H-^1^H COSY spectrum of **9**.


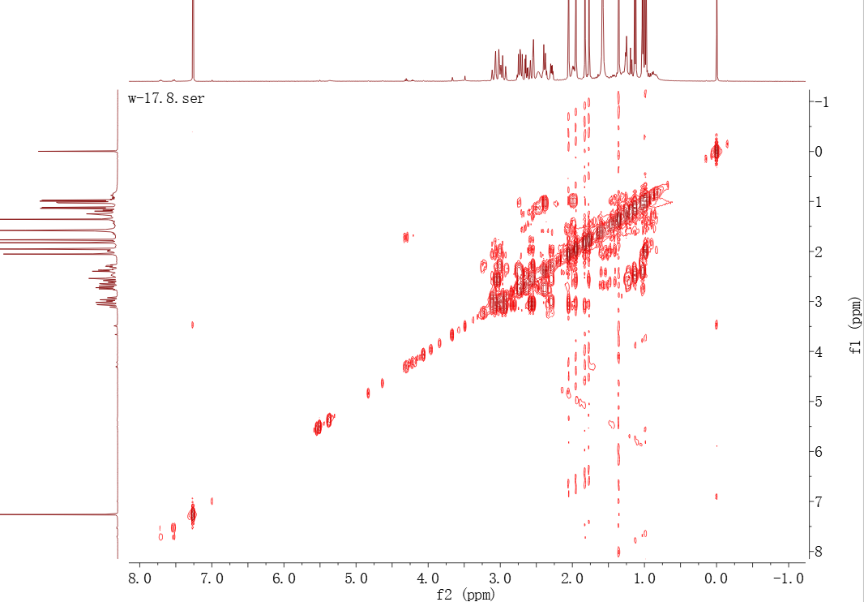


Figure S69. HSQC spectrum of **9**.


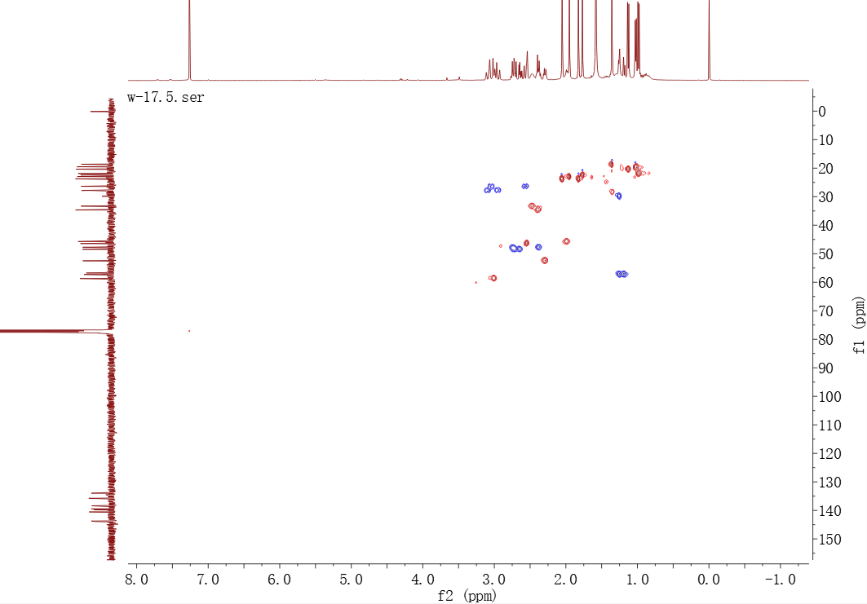


Figure S70. HMBC spectrum of **9**.


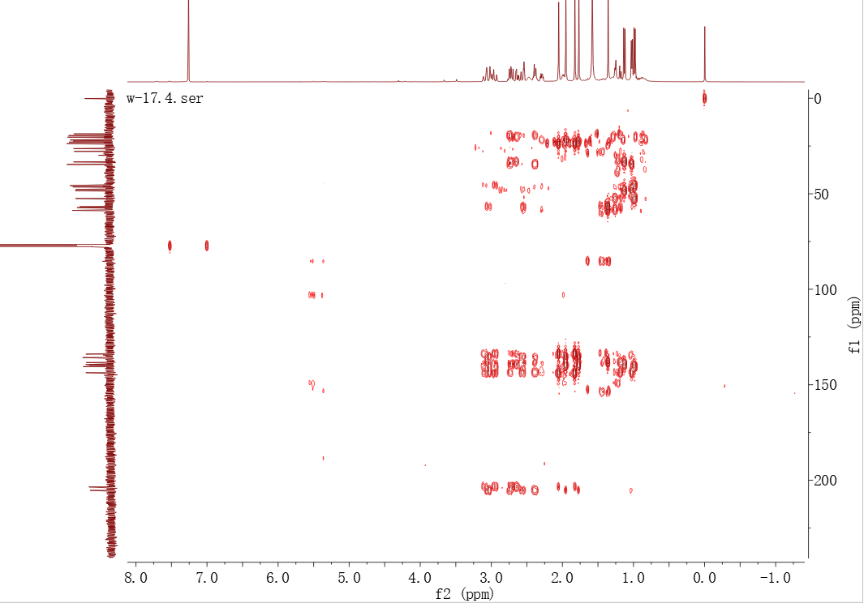


Figure S71. NOESY spectrum of **9**.


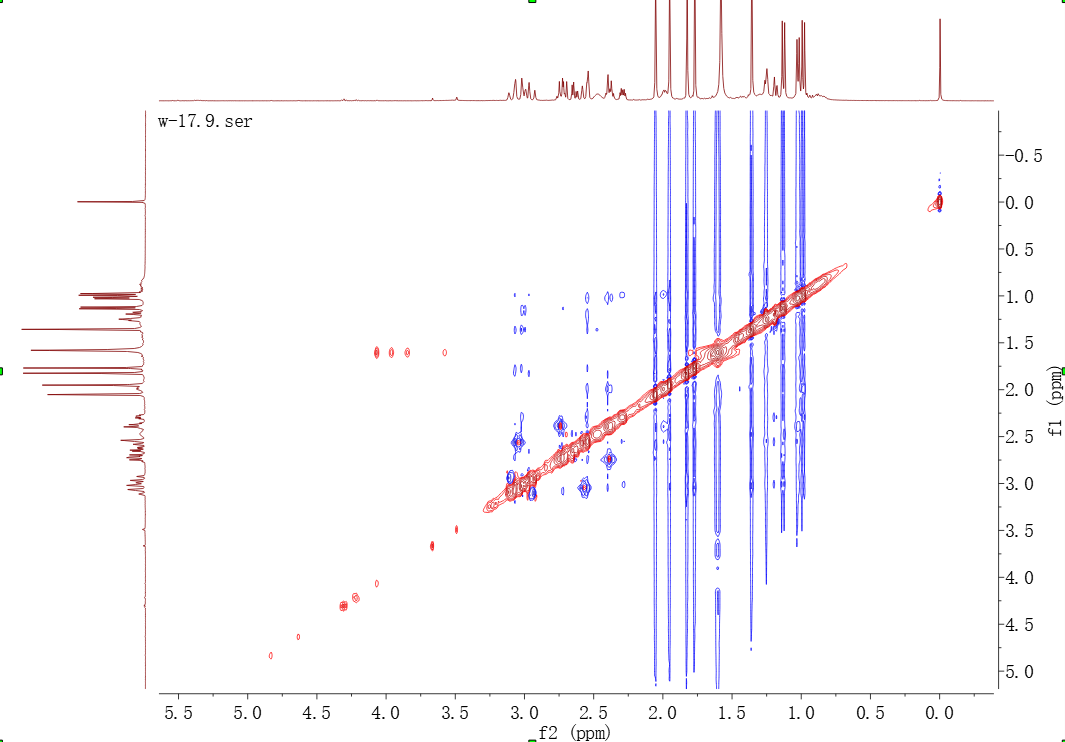


Figure S72. HR-ESI-MS spectrum of **9**.


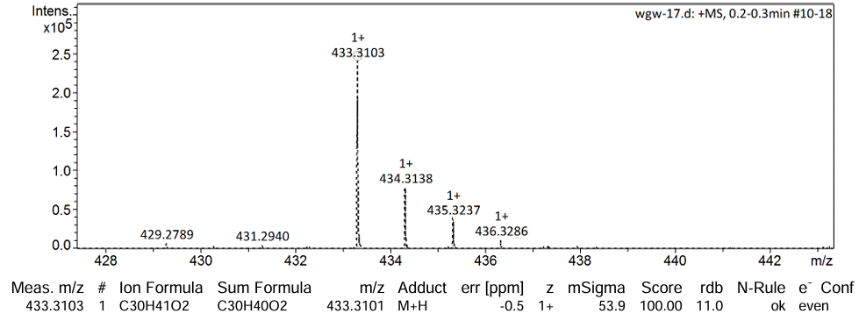


Figure S73. ^1^H NMR spectrum of **10**.


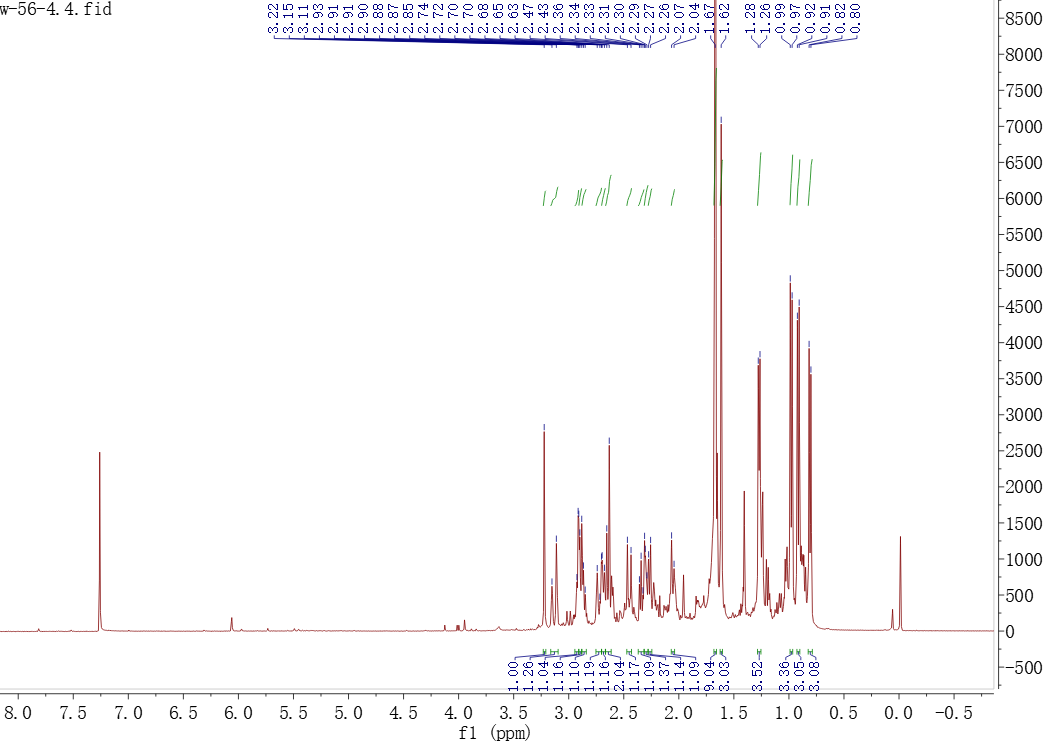


Figure S74. ^13^C NMR spectrum of **10**.


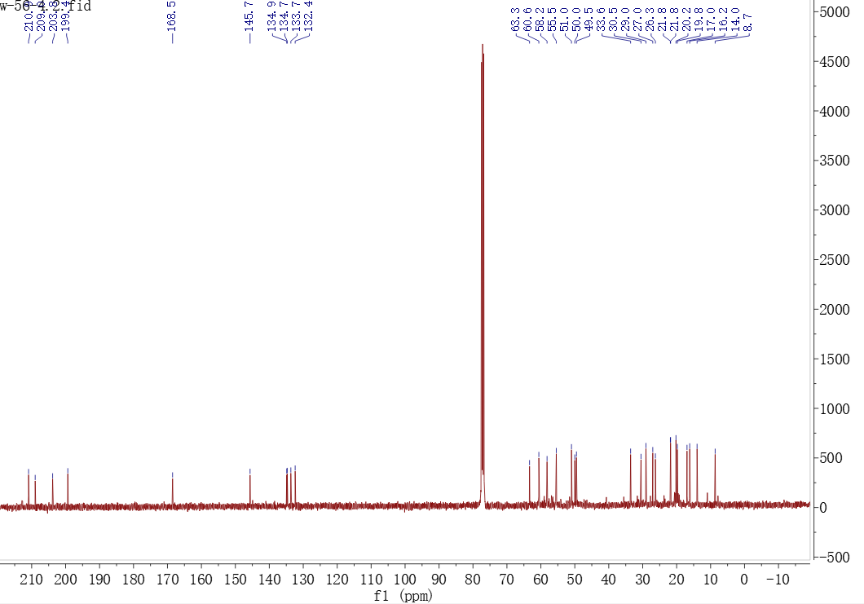


Figure S75. DEPT spectrum of **10**.


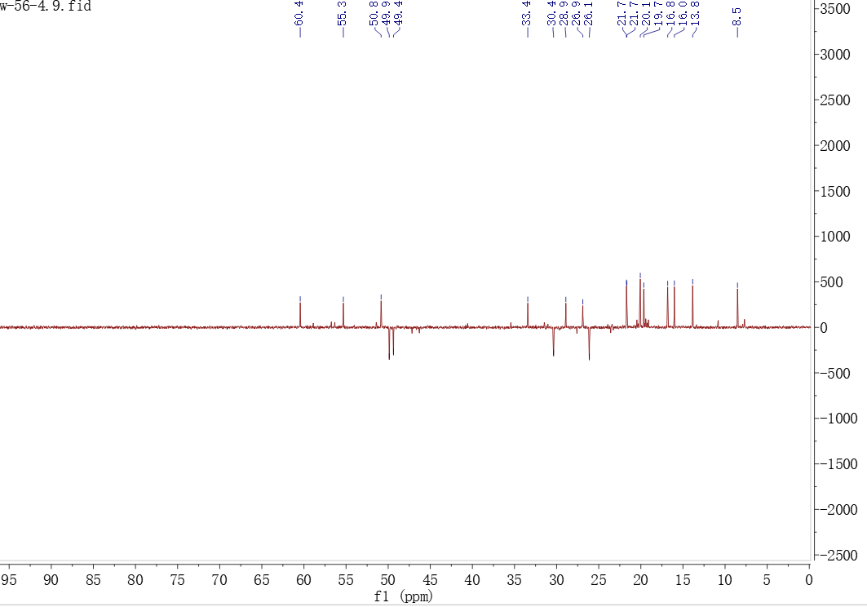


Figure S76. ^1^H-^1^H COSY spectrum of **10**.


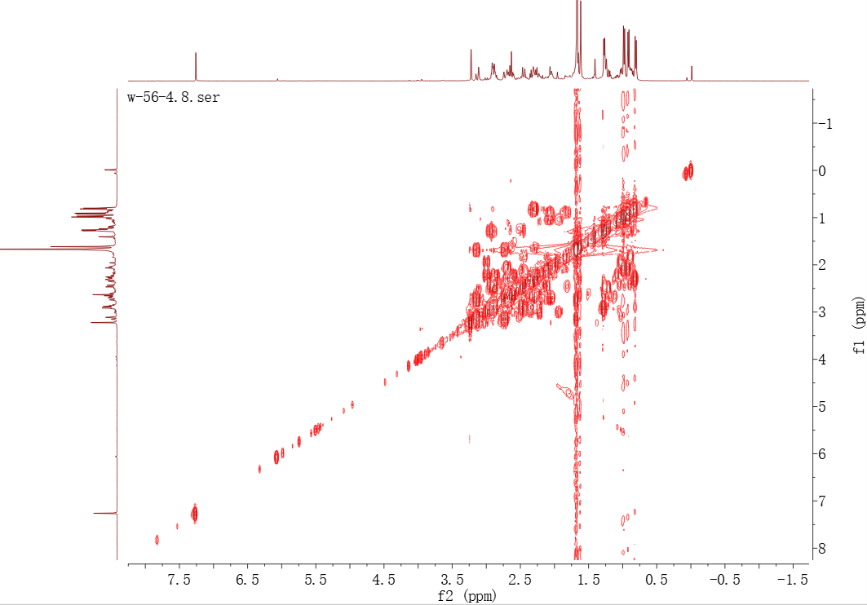


Figure S77. HSQC spectrum of **10**.


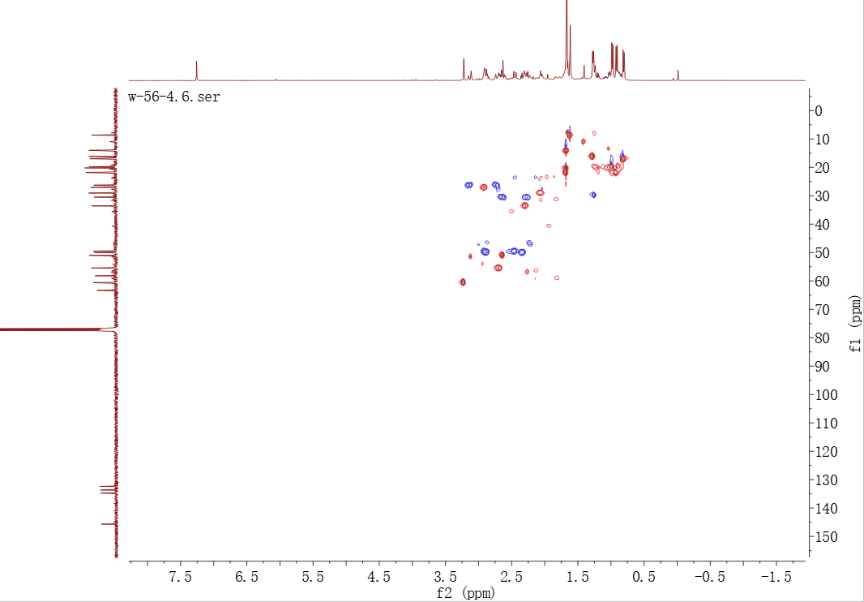


Figure S78. HMBC spectrum of **10**.


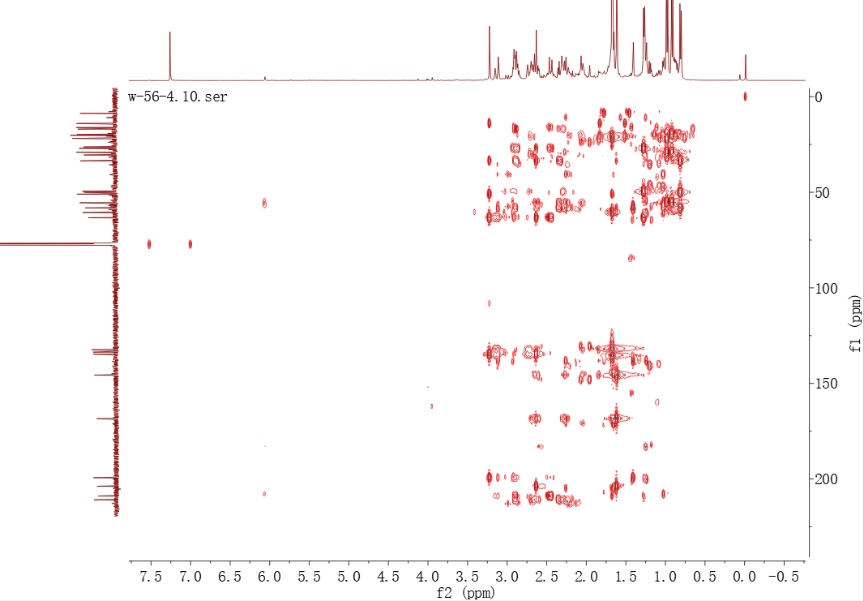


Figure S79. NOESY spectrum of **10**.


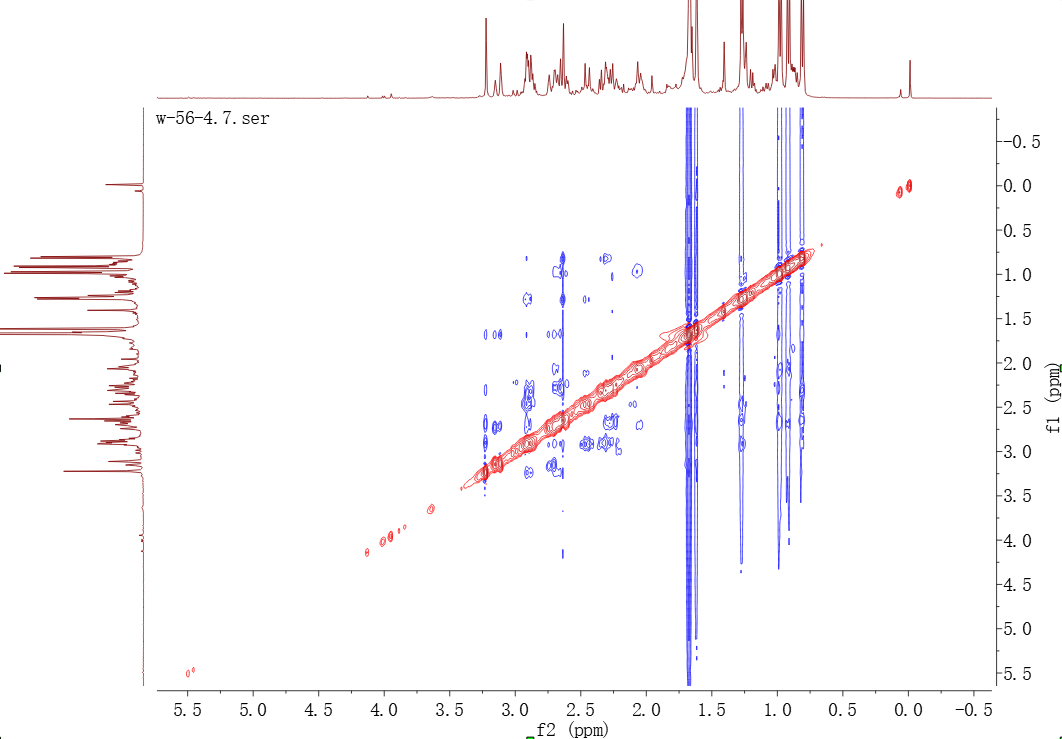


Figure S80. HR-ESI-MS spectrum of **10**.


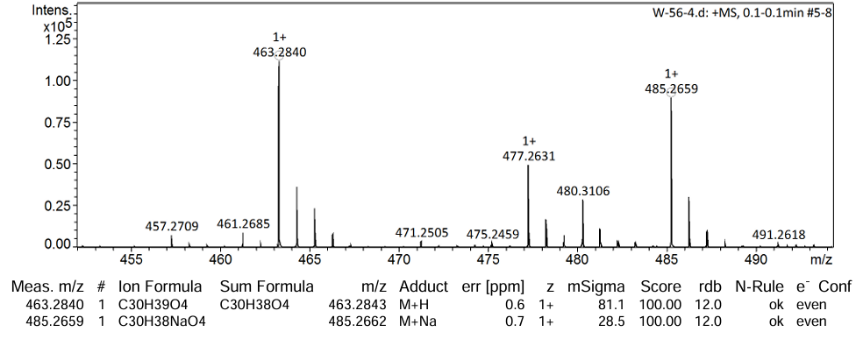


Figure S81. ^1^H NMR spectrum of **11**.


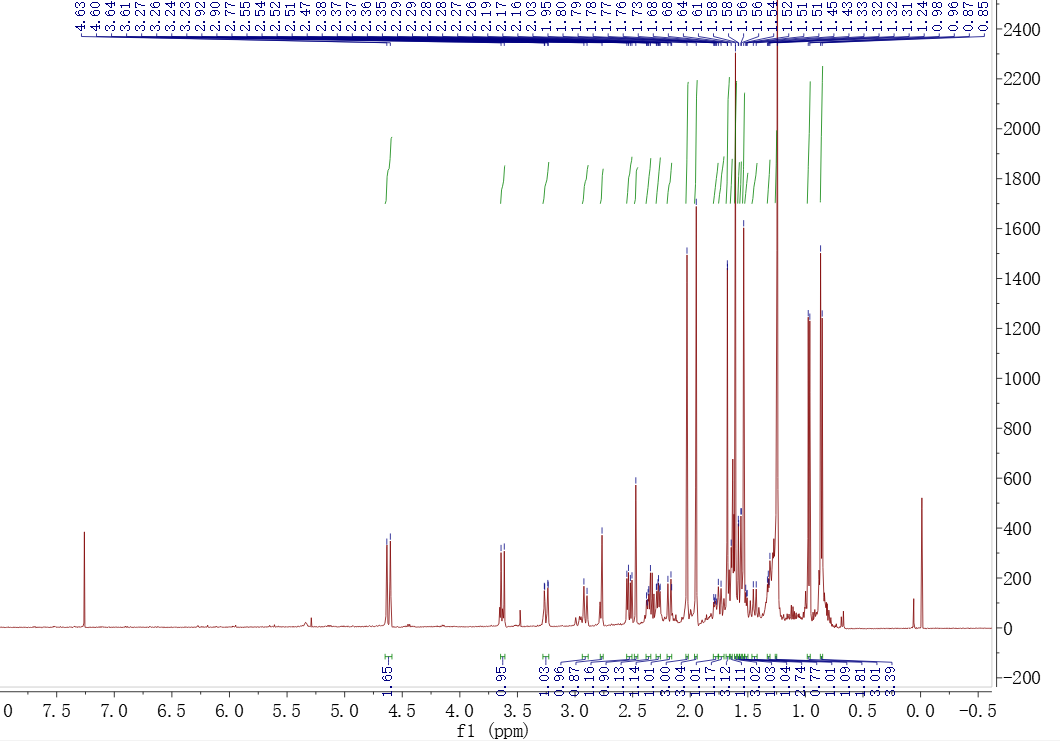


Figure S82. ^13^C NMR spectrum of **11**.


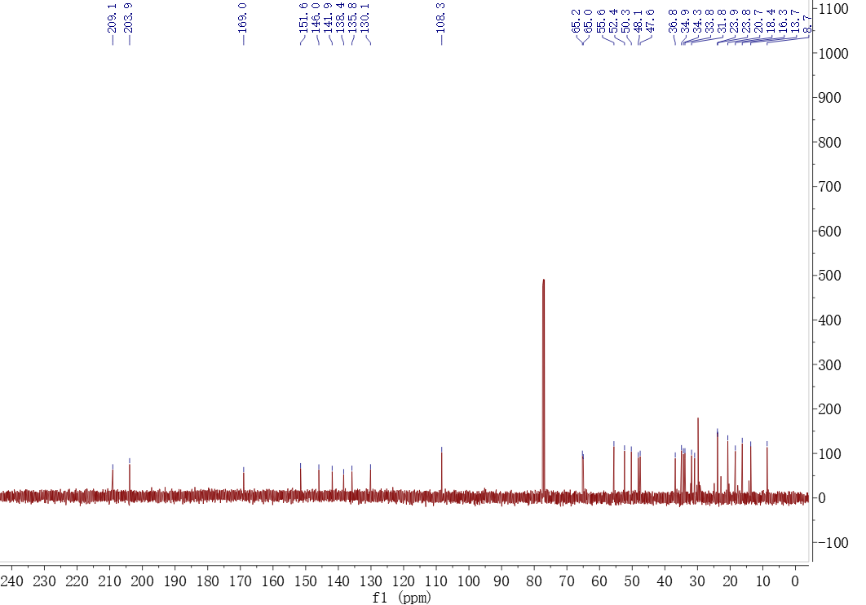


Figure S83. DEPT spectrum of **11**.


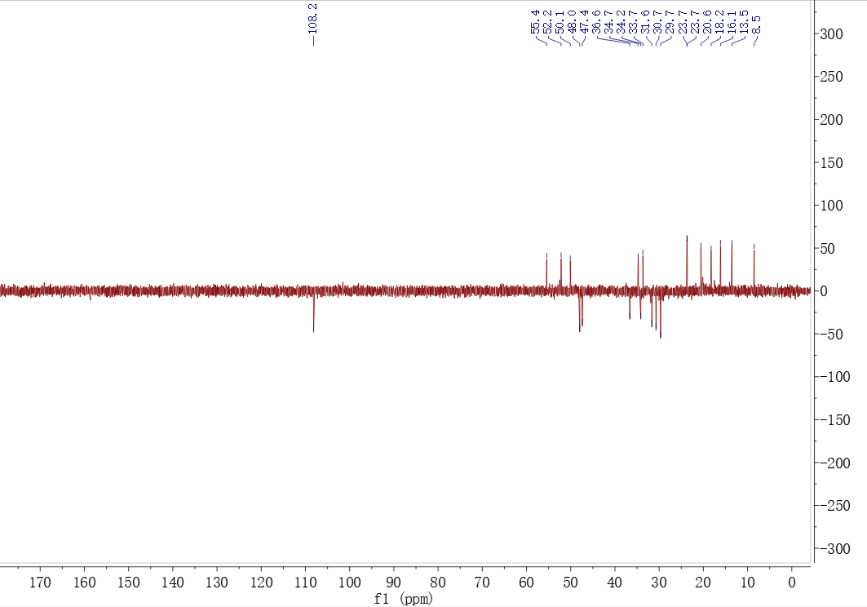


Figure S84. ^1^H-^1^H COSY spectrum of **11**.


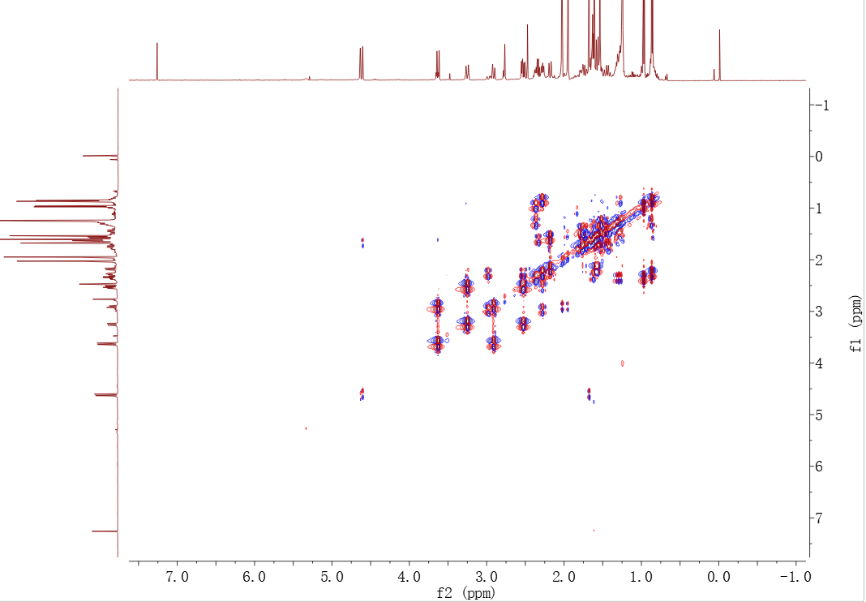


Figure S85. HSQC spectrum of **11**.


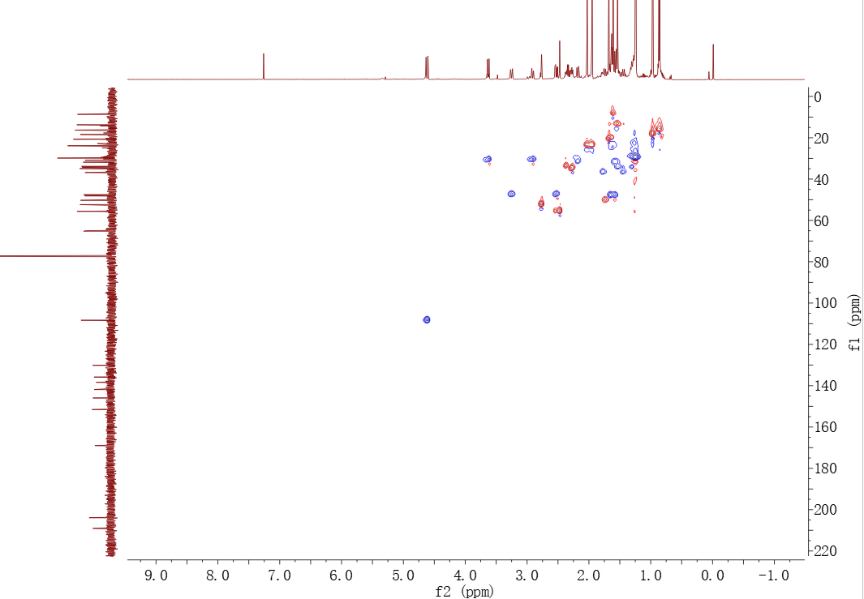


Figure S86. HMBC spectrum of **11**.


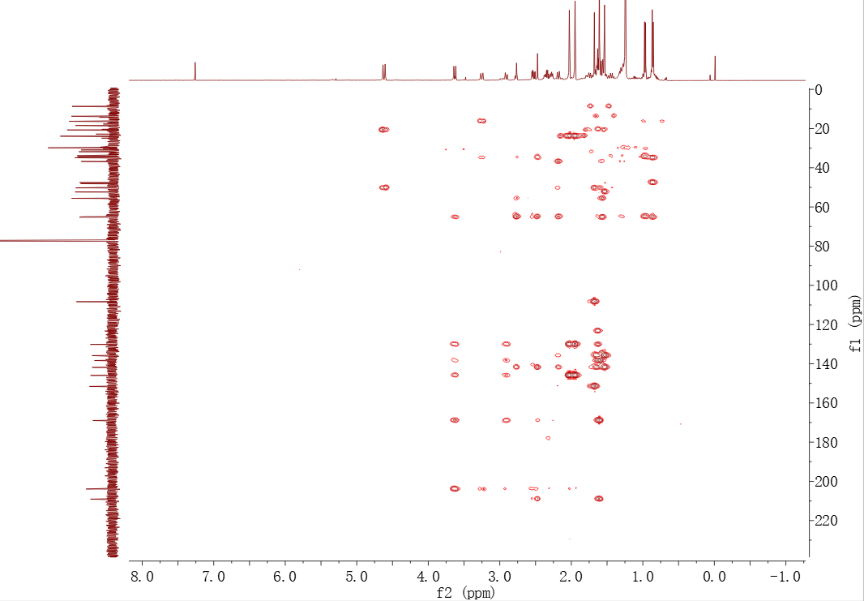


Figure S87. NOESY spectrum of **11**.


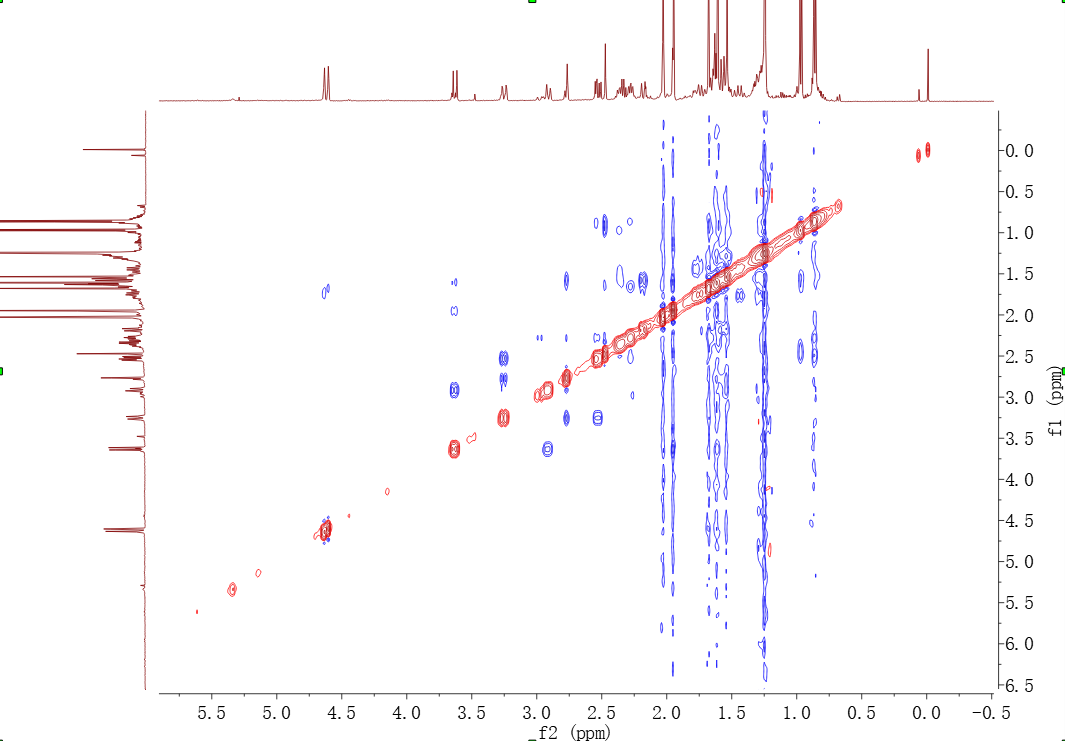


Figure S88. HR-ESI-MS spectrum of **11**.


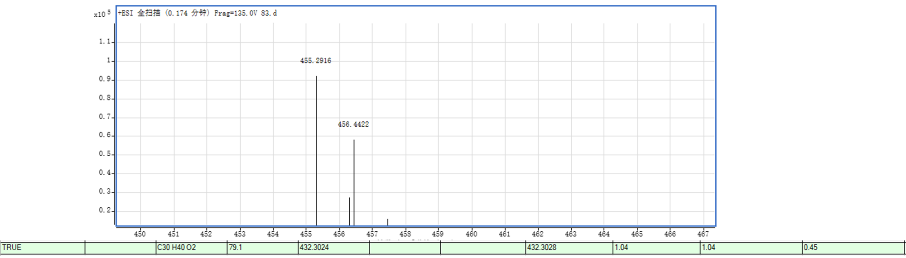


Figure S89. ^1^H NMR spectrum of **12**.


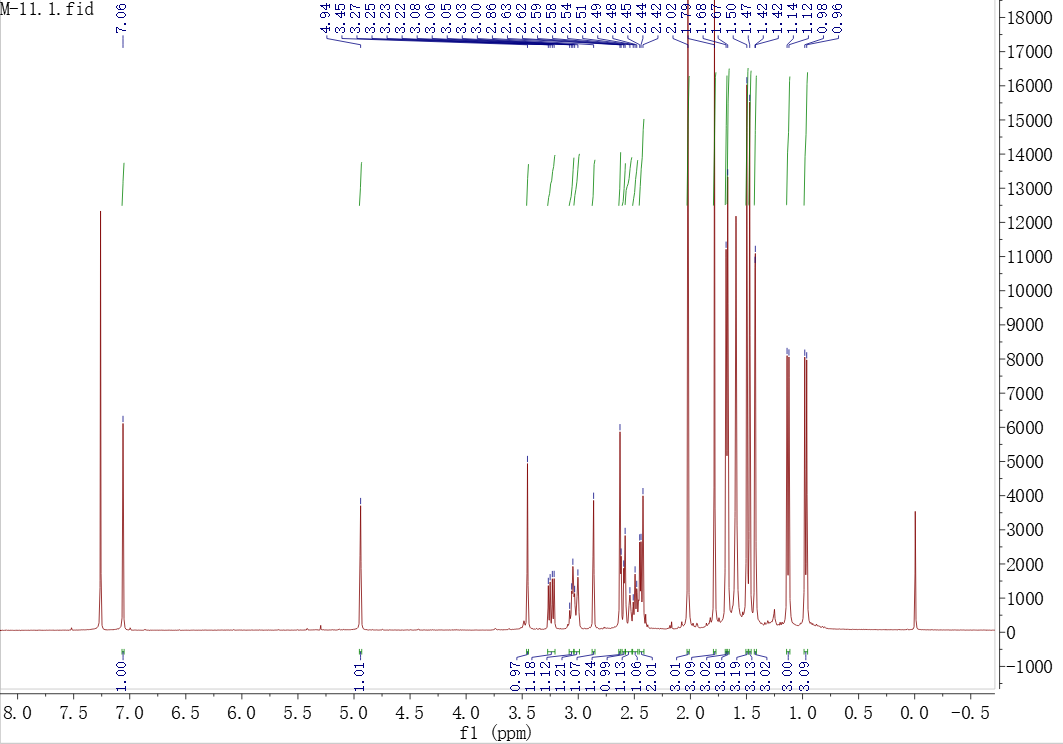


Figure S90. ^13^C NMR spectrum of **12**.


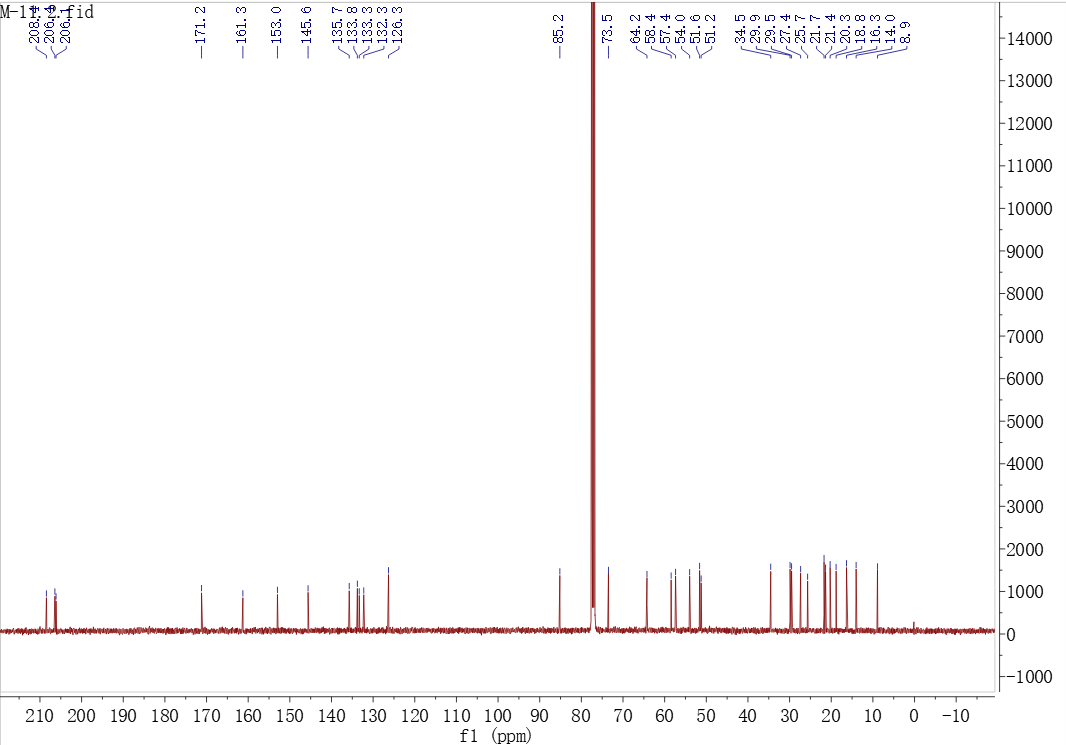


Figure S91. DEPT spectrum of **12**.


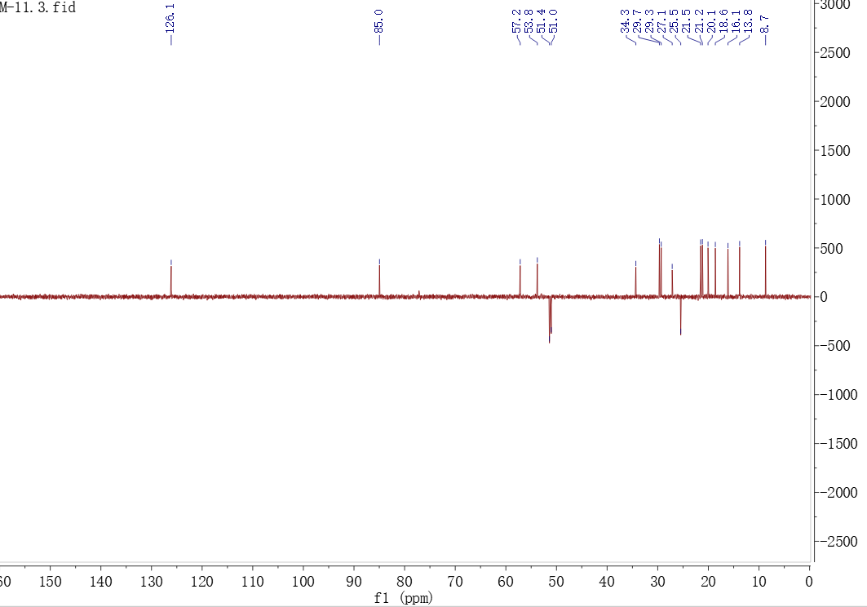


Figure S92. ^1^H-^1^H COSY spectrum of **12**.


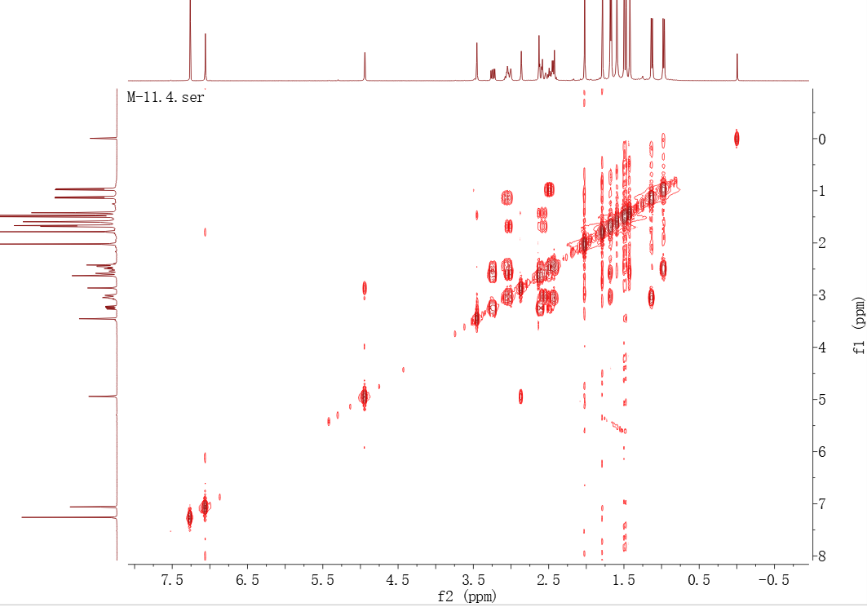


Figure S93. HSQC spectrum of **12**.


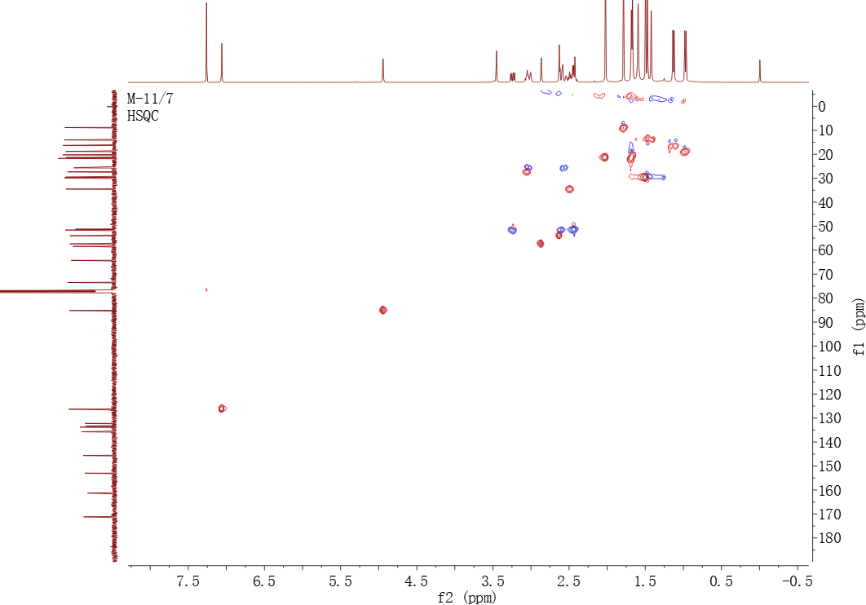


Figure S94. HMBC spectrum of **12**.


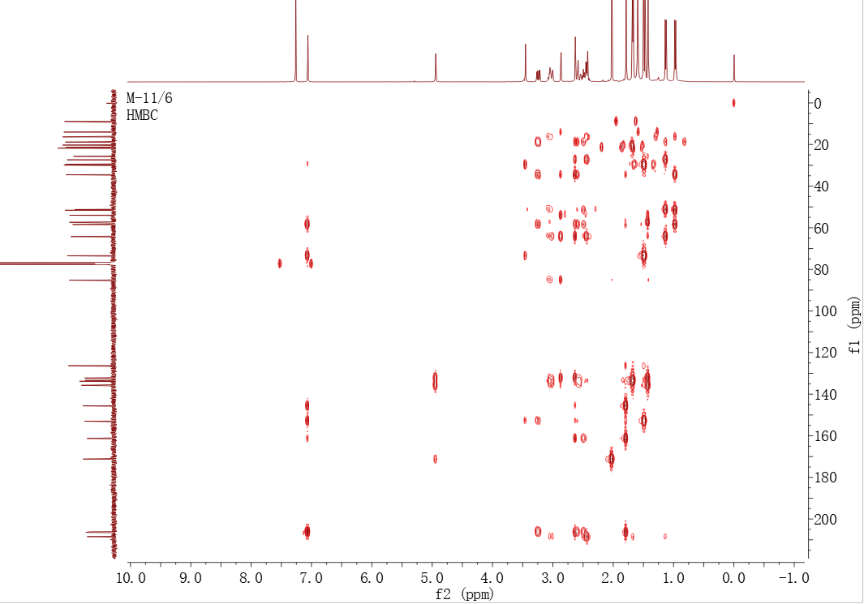


Figure S95. NOESY spectrum of **12**.


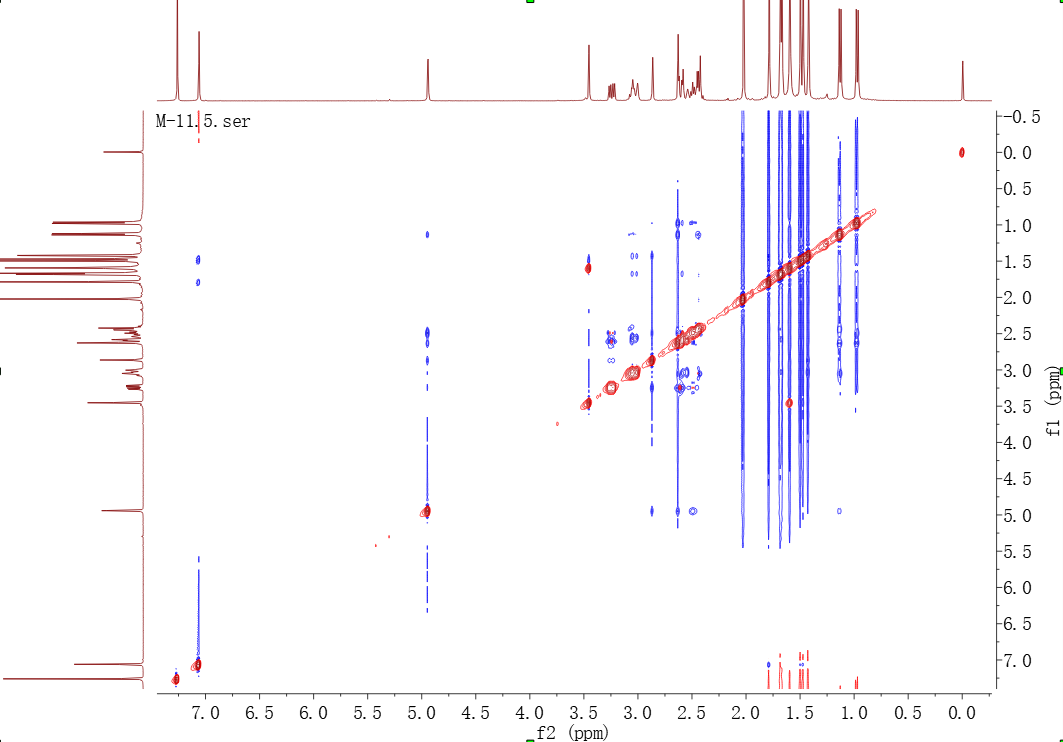


Figure S96. HR-ESI-MS spectrum of **12**.


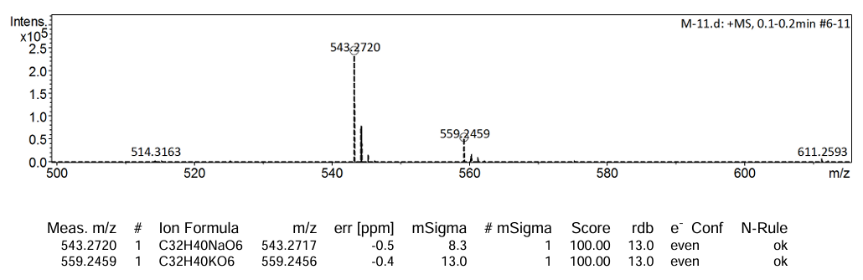


Figure S97. ^1^H NMR spectrum of **13**.


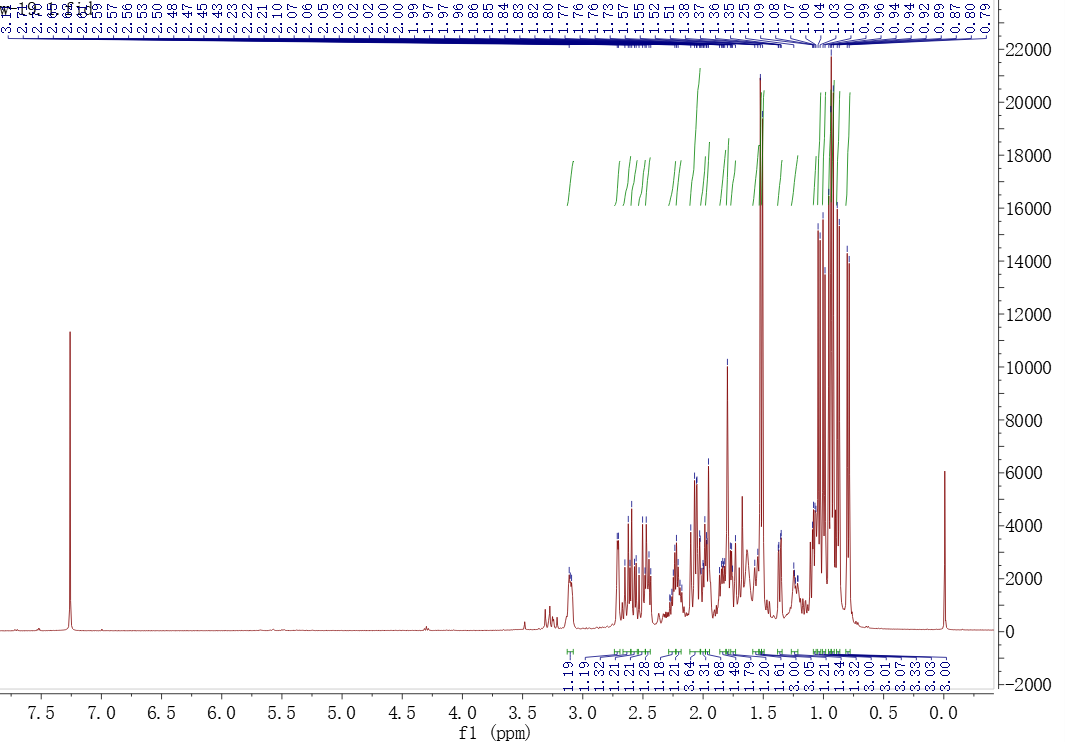


Figure S98. ^13^C NMR spectrum of **13**.


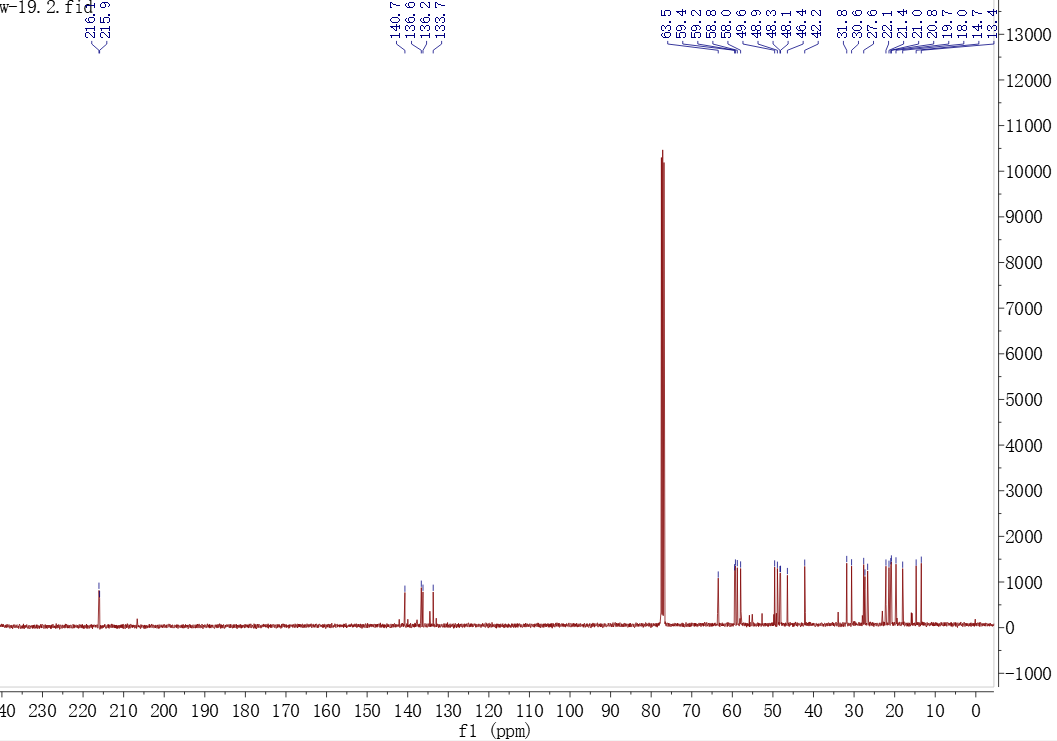


Figure S99. DEPT spectrum of **13**.


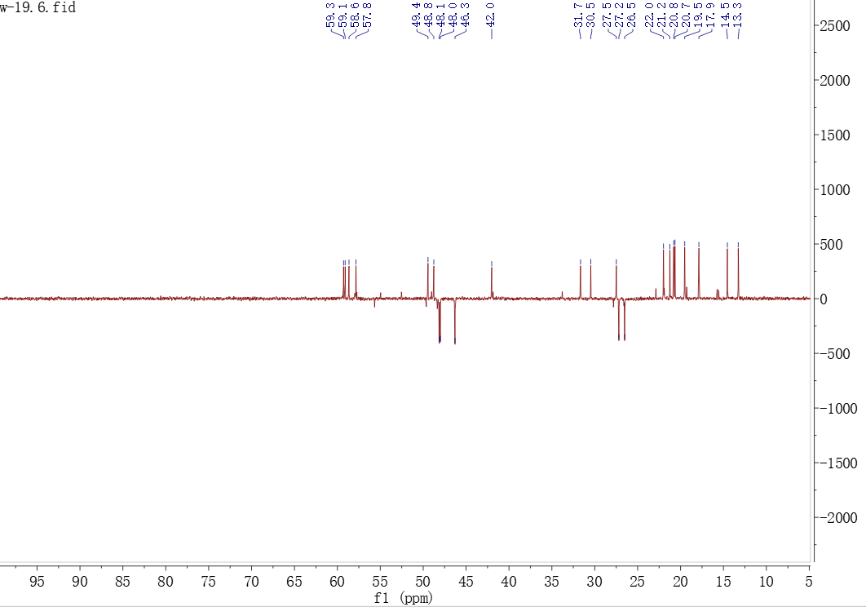


Figure S100. ^1^H-^1^H COSY spectrum of **13**.


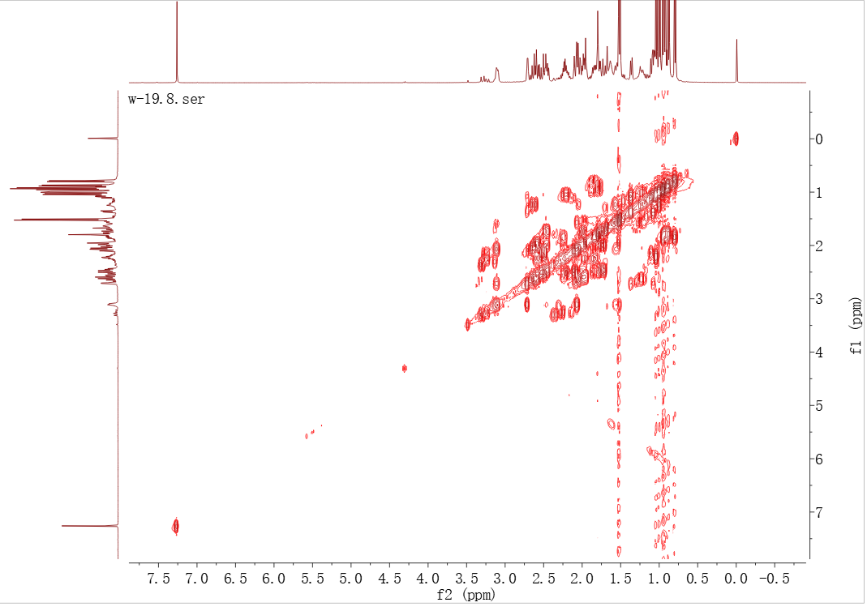


Figure S101. HSQC spectrum of **13**.

Figure S102. HMBC spectrum of **13**.

Figure S103. NOESY spectrum of **13**.

Figure S104. HR-ESI-MS spectrum of **13**.

Figure S105. ^1^H NMR spectrum of **14**.

Figure S106. ^13^C NMR spectrum of **14**.

Figure S107. DEPT spectrum of **14**.

Figure S108. ^1^H-^1^H COSY spectrum of **14**.

Figure S109. HSQC spectrum of **14**.

Figure S110. HMBC spectrum of **14**.

Figure S111. NOESY spectrum of **14**.

Figure S112. HR-ESI-MS spectrum of **14**.

Figure S113. ^1^H NMR spectrum of **15**.

Figure S114. ^13^C NMR spectrum of **15**.

Figure S115. DEPT spectrum of **15**.

Figure S116. ^1^H-^1^H COSY spectrum of **15**.

Figure S117. HSQC spectrum of **15**.

Figure S118. HMBC spectrum of **15**.

Figure S119. NOESY spectrum of **15**.

Figure S120. HR-ESI-MS spectrum of **15**.

Figure S121. ^1^H NMR spectrum of **16**.

Figure S122. ^13^C NMR spectrum of **16**.

Figure S123. DEPT spectrum of **16**.

Figure S124. ^1^H-^1^H COSY spectrum of **16**.

Figure S125. HSQC spectrum of **16**.

Figure S126. HMBC spectrum of **16**.

Figure S127. NOESY spectrum of **16**.

Figure S128. HR-ESI-MS spectrum of **16**.
